# Varicella‐Zoster virus ORF9 is an antagonist of the DNA sensor cGAS

**DOI:** 10.15252/embj.2021109217

**Published:** 2022-06-07

**Authors:** Jonny Hertzog, Wen Zhou, Gerissa Fowler, Rachel E Rigby, Anne Bridgeman, Henry TW Blest, Chiara Cursi, Lise Chauveau, Tamara Davenne, Benjamin E Warner, Paul R Kinchington, Philip J Kranzusch, Jan Rehwinkel

**Affiliations:** ^1^ MRC Human Immunology Unit MRC Weatherall Institute of Molecular Medicine Radcliffe Department of Medicine University of Oxford Oxford UK; ^2^ Department of Microbiology Harvard Medical School Boston MA USA; ^3^ Department of Cancer Immunology and Virology Dana‐Farber Cancer Institute Boston MA USA; ^4^ Department of Ophthalmology University of Pittsburgh Pittsburgh PA USA; ^5^ Department of Microbiology and Molecular Genetics University of Pittsburgh Pittsburgh PA USA; ^6^ Parker Institute for Cancer Immunotherapy Dana‐Farber Cancer Institute Boston MA USA; ^7^ Present address: Clinical Cooperation Unit Virotherapy German Cancer Research Center (DKFZ) Heidelberg Germany; ^8^ Present address: School of Life Sciences Southern University of Science and Technology Shenzhen China

**Keywords:** cGAS, DNA sensing, ORF9, phase separation, Varicella‐Zoster virus, Immunology, Microbiology, Virology & Host Pathogen Interaction

## Abstract

Varicella‐Zoster virus (VZV) causes chickenpox and shingles. Although the infection is associated with severe morbidity in some individuals, molecular mechanisms that determine innate immune responses remain poorly defined. We found that the cGAS/STING DNA sensing pathway was required for type I interferon (IFN) induction during VZV infection and that recognition of VZV by cGAS restricted its replication. Screening of a VZV ORF expression library identified the essential VZV tegument protein ORF9 as a cGAS antagonist. Ectopically or virally expressed ORF9 bound to endogenous cGAS leading to reduced type I IFN responses to transfected DNA. Confocal microscopy revealed co‐localisation of cGAS and ORF9. ORF9 and cGAS also interacted directly in a cell‐free system and phase‐separated together with DNA. Furthermore, ORF9 inhibited cGAMP production by cGAS. Taken together, these results reveal the importance of the cGAS/STING DNA sensing pathway for VZV recognition and identify a VZV immune antagonist that partially but directly interferes with DNA sensing via cGAS.

## Graphical abstract

The DNA sensing cGAS/STING pathway plays a key role in detecting VZV infection and is in turn directly countered by a viral immunosuppressor.

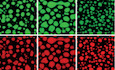

## Introduction

Varicella‐Zoster virus (VZV) is one of nine herpes viruses that infect humans (Arvin & Gilden, 2013). VZV is an alpha‐herpesvirus, closely related to herpes simplex virus (HSV) 1 and 2. It has a 125 kb dsDNA genome, the smallest of the human herpesviruses. The genome includes at least 70 open reading frames (ORFs). Primary infection causes chickenpox (*Varicella*). Like all human herpes viruses, VZV establishes life‐long latency in an infected host, and VZV can reactivate as shingles (*Zoster*). Shingles is a debilitating disease with significant associated morbidity. During both primary infection and reactivation, the virus can gain access to the central nervous system and cause severe complications such as encephalitis and vasculitis (Nagel & Gilden, 2014). Despite the introduction of the live‐attenuated chickenpox vaccine in the early 1990s, the virus remains highly prevalent worldwide (WHO, 2014).

The type I interferon (IFN) system lies at the forefront of host defence against infectious pathogens and is indispensable for the successful control of viral infections (McNab *et al*, 2015). The expression of type I IFNs is induced following pattern recognition receptor (PRR) activation. PRRs are a heterogenous group of proteins that can respond to a diverse array of pathogen‐associated molecular patterns (PAMPs; Brubaker *et al*, 2015). The recognition of viral pathogens relies to a large extent on the sensing of nucleic acids (Barrat *et al*, 2016; Hartmann, 2017). Both endosomal toll‐like receptors and dedicated cytosolic sensors are potently activated by viral RNA and DNA. The DNA sensor cyclic GMP‐AMP synthase (cGAS) synthesises the second messenger 2′3′‐cyclic GMP‐AMP (hereafter simply cGAMP), a cyclic dinucleotide, upon direct binding to dsDNA (Ablasser & Chen, 2019). Binding of cGAMP to stimulator of IFN genes (STING) results in the activation of the transcription factors IRF3 and NF‐κB via the kinases TBK1 and IKKε (Hopfner & Hornung, 2020). IRF3 and NF‐κB induce the expression of type I IFNs, type III IFNs, and inflammatory cytokines.

Type I IFNs, including IFNα and IFNβ, are secreted cytokines that act in an autocrine, paracrine, or systemic manner by binding to the type I IFN receptor (IFNAR; McNab *et al*, 2015). Canonical IFNAR signalling results in the phosphorylation and heterodimerisation of the transcription factors STAT1 and STAT2. After recruitment of IRF9, this protein complex drives expression of hundreds of genes, termed interferon‐stimulated genes (ISGs). Among others, ISGs include genes that encode PRRs, proteins involved in type I IFN induction and signalling, negative and positive feedback regulators, restriction factors acting directly on viruses, and proteins that are involved in adaptive immune responses (Schoggins, 2019).

Despite the fact that VZV is a highly prevalent and important human pathogen, its pathogenesis is still poorly understood. The lack of suitable small animal models that recapitulate primary infection and latency establishment has hindered the molecular characterisation of its life cycle *in vivo* (Haberthur & Messaoudi, 2013). During its dissemination in the human host, the virus infects a multitude of different cells. Infection of T cells, keratinocytes, neurons, and epithelial cells is indispensable for VZV’s life cycle (Zerboni *et al*, 2014). In addition, immune cells including dendritic cells (DCs), monocytes, and NK cells are capable of supporting VZV replication *in vitro* and are potentially relevant for *in vivo* spread (Abendroth *et al*, 2001; Morrow *et al*, 2003; Wang *et al*, 2005; Campbell *et al*, 2018; Kennedy *et al*, 2019). Current evidence suggests that type I IFNs are critical for the control of VZV infection. Increased IFNα levels can be detected in the serum of patients with primary VZV infection (Arvin *et al*, 1986; Zerboni *et al*, 2014). In addition, type I IFNs limit VZV replication *in vitro* (Torigo *et al*, 2000; Ku *et al*, 2016; Kim *et al*, 2017; Shakya *et al*, 2019). However, the events that govern the cell‐intrinsic recognition of the virus in the various cell types it infects and induction of the antiviral cytokine response have only begun to be elucidated *in vitro*. The DNA sensor TLR9 is partly responsible for IFNα secretion after infection of plasmacytoid DCs (Yu *et al*, 2011). In dermal fibroblasts, STING is required for type I and type III IFN production (Kim *et al*, 2017). An interesting genetic link between DNA sensing via RNA polymerase III and infection of the central nervous system by VZV has been uncovered recently (Carter‐Timofte *et al*, 2018). However, a comprehensive characterisation of the role of DNA sensing during VZV infection is still lacking.

In this study, we tested which nucleic acid sensors induce type I IFN expression in response to VZV infection. We show that the cGAS–STING–TBK1–IRF3 signalling axis was responsible for antiviral cytokine expression after VZV infection. We further report the generation of a VZV open reading frame (ORF) expression library and identification of a viral cGAS antagonist. The tegument protein encoded by ORF9 curtailed the activation of cGAS and subsequent synthesis of cGAMP. Mechanistically, we show that ORF9 interacted with cGAS and DNA. This resulted in decreased cGAMP and IFN production. We propose a model in which cGAS activation upon VZV infection is limited immediately after viral entry through the tegument protein ORF9.

## Results

### Induction of the type I IFN response to VZV infection in THP1 cells requires the DNA sensor cGAS

To identify PRRs that induce type I IFNs in response to VZV infection, we used the monocytic cell line THP1. We hypothesised a role of DNA sensors in VZV infection, particularly given its identity as a DNA virus and the previously shown role of STING in recognition of VZV (Kim *et al*, 2017). THP1 cells, unlike many other immortalised cell lines, induce type I and type III IFNs *via* cGAS in response to DNA (Sun *et al*, 2013; Wu *et al*, 2013). Furthermore, THP1 cells are amenable to genome editing and can be used to genetically dissect the role of individual proteins involved in pattern recognition. THP1 cells are permissive for VZV infection and propagation (Nour *et al*, 2011), and VZV infects primary human monocytes and macrophages *in vitro* and *in vivo* (Mainka *et al*, 1998; Kennedy *et al*, 2019). In addition to wild‐type (WT) THP1 cells, we tested previously described knockout (KO) lines lacking STING, TBK1, MyD88, or IFNAR2. We further generated cGAS‐KO, MAVS‐KO, and IRF3‐KO cells using CRISPR/Cas9 technology (see Materials and Methods and Appendix Fig S1 and S2). All THP1‐KO cells were validated by immunoblotting for the absence of protein and functionally by stimulation with DNA, RNA, and type I IFN (Appendix Fig S1 and S2). These cells contained a secreted luciferase reporter construct under control of an IRF3‐responsive promoter.

**Figure EV1:**
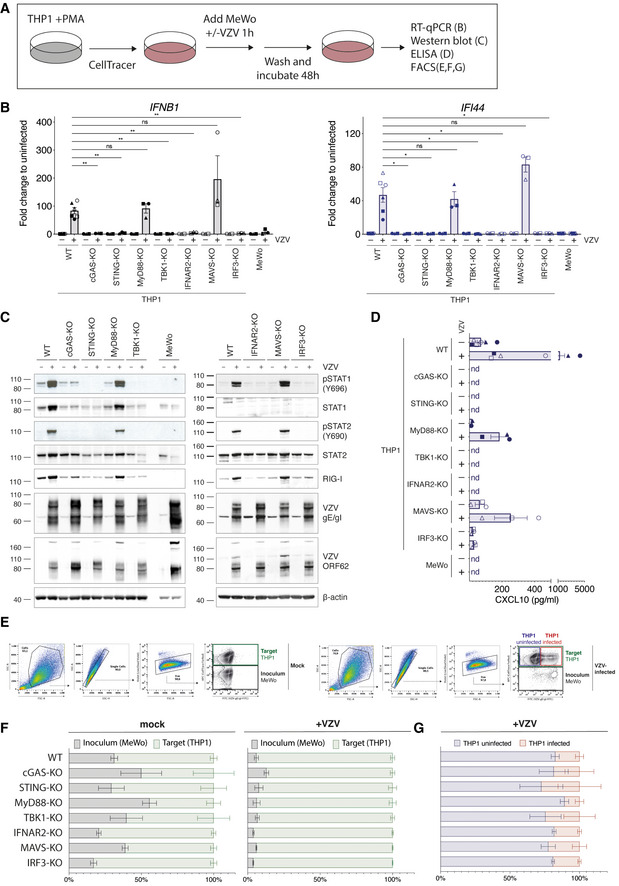
The cGAS/STING DNA sensing pathway induces type I IFNs in response to VZV infection. Related to Fig 1 Schematic of the THP1‐MeWo co‐culture VZV infection system. See text for details.A panel of THP1 knockout cell lines was mock infected or infected with VZV as shown in (A). The mRNA expression levels of *IFNB1* (encodes IFNβ) and *IFI44* were analysed by RT–qPCR. Expression levels were normalised to *GAPDH* and are shown as fold changes relative to levels in uninfected cells.Cells infected as in (A) were analysed by western blot using the indicated antibodies.Levels of CXCL10 (IP‐10) in co‐culture supernatants were quantified by ELISA.WT THP1 cells infected as in (A) were analysed by flow cytometry. Left and right panels show gating strategy for (F) and (G), respectively.Quantification of inoculum (MeWo) and target cells (THP1) in co‐culture experiments.Quantification of VZV‐infected cells within THP1 target cell population. Data information: The different shapes of data points in (B) and (D) correspond to independent biological repeat experiments. Panels (B), (D), (F), and (G) show pooled data from six (THP1 WT, MeWo) or three (THP1 KOs) independent biological repeats (*n* = 3/6 ± SEM). Panel (C) shows a representative result of three independent repeats. Panel (E) shows a representative result of six independent repeats. Statistical analysis in panel (B) was one‐way ANOVA with Dunnett’s multiple comparisons test. **P < 0.01, *P < 0.05, ns = not significant.

Upon treatment with PMA, THP1 cells adopt a macrophage‐like, adherent, and highly responsive phenotype. Given the difficulties of working with cell‐free VZV (Chen *et al*, 2004; Caunt & Taylor‐Robinson, 2009), we used co‐culture with VZV‐infected (+VZV) MeWo cells to infect PMA‐treated THP1 cells; co‐culture with uninfected MeWo cells served as a control (Fig EV1A). MeWo cells are a melanoma cell line that is well‐established for VZV propagation. After 48 h of co‐culture, cells were harvested for RT–qPCR and immunoblotting. For all experiments, uninfected MeWo cells were additionally used as target cells. MeWo cells do not induce type I IFNs in response to VZV (Fig EV1B).

To investigate whether THP1 cells induce type I IFNs in response to VZV, we analysed mRNA expression levels of *IFNB1* (encodes IFNβ) and *IFI44*, an ISG. In WT THP1 cells, both transcripts were robustly induced after VZV infection (Fig EV1B). Similar results were obtained using MyD88‐KO and MAVS‐KO cells. In contrast, no transcriptional upregulation of *IFNB1* or *IFI44* was observed in THP1 cells lacking cGAS, STING, TBK1, IRF3, or IFNAR2. Moreover, immunoblot analysis showed that the transcription factors STAT1 and STAT2 were only phosphorylated in WT, MyD88‐KO, and MAVS‐KO cells (Fig EV1C). No p‐STAT1 and p‐STAT2 signals were observed in cells lacking cGAS, STING, TBK1, IRF3, or IFNAR2. This indicated that only WT, MyD88 KO, and MAVS‐KO cells secreted type I IFNs in response to VZV infection. Consistently, STAT1 and RIG‐I, which are both encoded by ISGs, were upregulated at protein level only in the cells showing STAT1/2 activation (Fig EV1C). Importantly, we could not observe phosphorylation of STAT1/2 or increased abundance of STAT1 and RIG‐I in infected MeWo cells. Western blotting with antibodies against VZV‐glycoprotein E (gE)/glycoprotein I (gI) and VZV ORF62 confirmed that all cell lines became infected (Fig EV1C). Determination of CXCL10 (IP‐10) levels in co‐culture supernatants confirmed the findings of our RT–qPCR and immunoblot analyses. WT, MyD88 KO, and MAVS‐KO THP1 cells produced low levels of CXCL10 at baseline, and these were increased after infection with VZV (Fig EV1D). No CXCL10 was detected in supernatants from uninfected cells and in samples from infected cGAS‐KO, STING‐KO, TBK1‐KO, and IFNAR2‐KO THP1 cells; similarly, MeWo cells did not secrete CXCL10. We could detect low levels of CXCL10 in supernatants from IRF3‐KO cells, but there was no increase above baseline after infection. Analysis of co‐cultured cells by flow cytometry (cell‐surface staining for the VZV‐gE/gI complex (Mo *et al*, 2003)) showed that the proportion of inoculum MeWo cells and THP1 target cells differed between samples, in particular for uninfected conditions (Fig EV1E and F). Within the population of THP1 target cells, the percentage of VZV‐infected cells was variable and no clear differences between THP1‐KO cell lines could be observed (Fig EV1E and G). It is likely that this variability is partly because the different THP1‐KO cells were obtained from various sources. Collectively, these results suggest that in THP1 cells, the induction of the type I IFN in response to VZV infection required the DNA sensor cGAS and the STING–TBK1–IRF3 signalling axis. It is therefore likely that dsDNA is a PAMP recognised in VZV‐infected cells.

**Figure 1:**
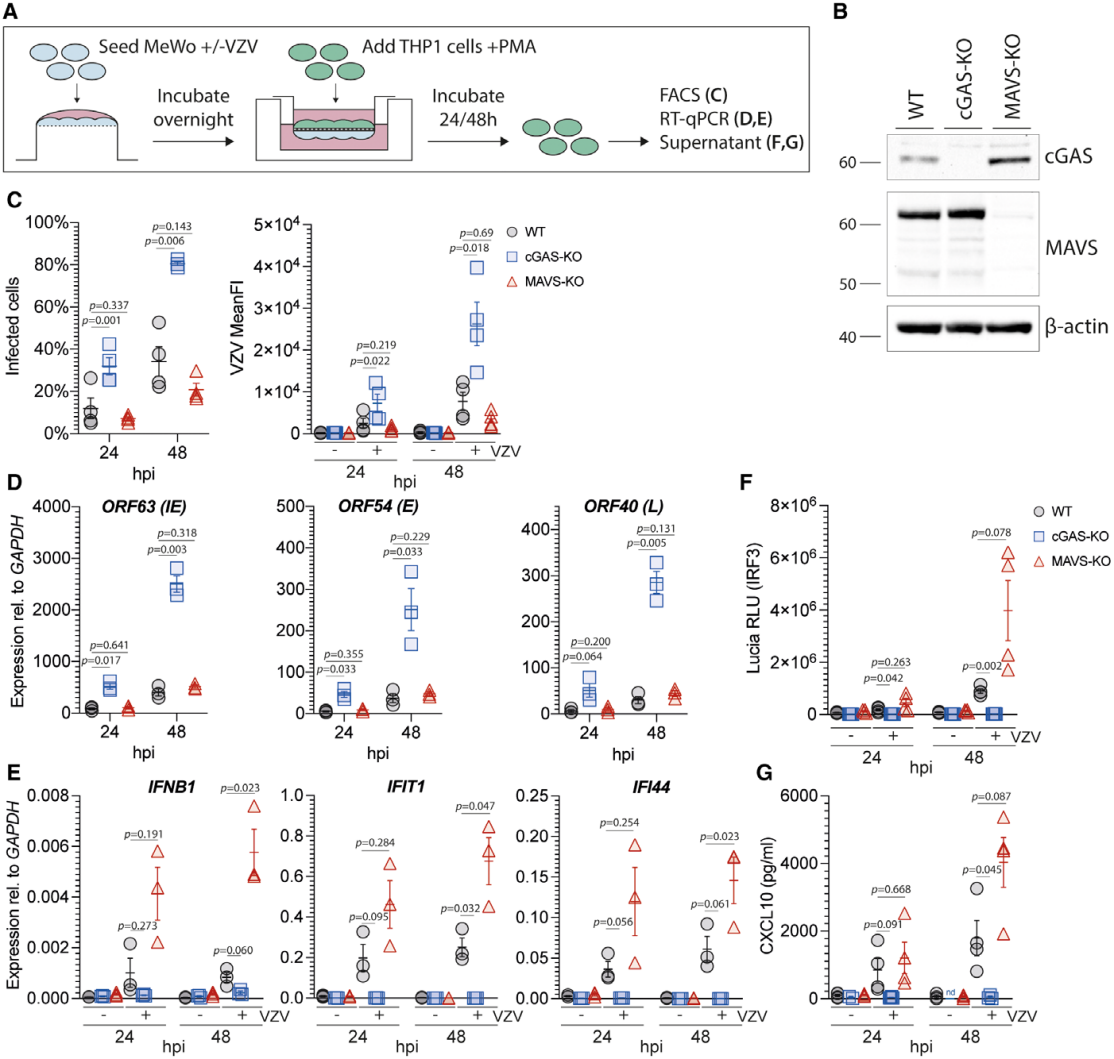
cGAS induces type I IFNs in response to VZV infection Schematic detailing the experimental procedure for infection of PMA‐differentiated THP1 cells with VZV by transwell assay. See text for details.Immunoblot of WT, cGAS‐KO, and MAVS‐KO THP1 cells used in (C–G).THP1 cells of the indicated genotypes were mock infected or infected with VZV for 24 and 48 h using the transwell assay described in (A). Infected cells were quantified by surface staining for VZV‐gE/gI and flow cytometry analysis. See Appendix Fig S3 for gating.RT–qPCR analysis of VZV ORF63 (immediate early (IE) gene), ORF54 (early (E) gene), and ORF40 (late (L) gene) transcripts in cells infected as in (C). Graphs show expression relative to *GAPDH*.RT–qPCR analysis of *IFNB1*, *IFI44*, and *IFIT1* expression in cells infected as in (C). Graphs show expression relative to *GAPDH*.Activity of Lucia luciferase (secreted under an IRF3‐dependent promoter) was determined in supernatants of cells infected as in (C) by QUANTI‐Luc assay.Concentrations of CXCL10 in supernatants of cells infected as in (C) were determined by ELISA. Data information: Panel (B) is representative of two independent biological experiments. Panels (C), (F), and (G) show pooled data from four repeats, where each data point represents an independent biological experiment (*n* = 4 ± SEM). Panels (D) and (E) show pooled data from three repeats, where each data point represents an independent biological experiment (*n* = 3 ± SEM). Statistical analysis in panels (C), (D), (F), and (G) was paired *t*‐tests and in panel (E) paired ratio *t*‐tests. hpi: hours post infection, WT: wild type, KO: knockout. See also Fig EV1 and Appendix Fig S1, Fig S2, and Fig S3. Source data are available online for this figure.

To further dissect the role of the DNA sensor cGAS in the recognition of VZV, we developed a transwell‐based infection system (Fig 1A). In this setup, infected MeWo cells are first seeded on the bottom side of the transwell membrane. After adherence, THP1 target cells are seeded on the opposite side of the membrane. The membrane contains 1 µm pores through which cell–cell contacts can be established and VZV can spread. Importantly, the inoculum and target cells do not mix, and a homogenous target cell population can be harvested for analysis. We anticipate that this new infection protocol (see Materials and Methods for details) will be widely applicable to many VZV research projects.

WT, cGAS‐KO, and MAVS‐KO THP1 (Fig 1B) cells were infected for 24 and 48 h using this transwell assay. Of note, the cGAS‐KO and MAVS‐KO cells were both generated as part of this study to ensure consistency (Appendix Fig S1). Flow cytometry analysis revealed significantly higher levels of infection in cGAS‐KO cells compared to WT and MAVS‐KO cells at both time points (Fig 1C and Appendix Fig S3). Additionally, the mean fluorescence intensity (MFI) of VZV‐gE/gI staining was significantly higher in cGAS‐KO cells compared to WT and MAVS‐KO cells (Fig 1C). We further found increased expression of immediate early (IE), early (E), and late (L) viral gene products in cells lacking cGAS (Fig 1D). In line with our previous results, THP1 cells failed to upregulate *IFNB1* and ISG expression after VZV infection in the absence of cGAS (Fig 1E). In WT and MAVS‐KO cells, VZV infection robustly induced secretion of the IRF3‐controlled luciferase reporter and CXCL10 (Fig 1F and G). This response was undetectable in cGAS‐KO cells.

These results confirm our previous observations from the co‐culture system and establish that the induction of the type I IFN response to VZV infection in THP1 cells was mediated by the DNA sensor cGAS. Significantly more cells became infected with VZV in the absence of cGAS, indicating that recognition by cGAS was required for restriction of VZV infection.

### A VZV ORF expression library

**Figure EV2:**
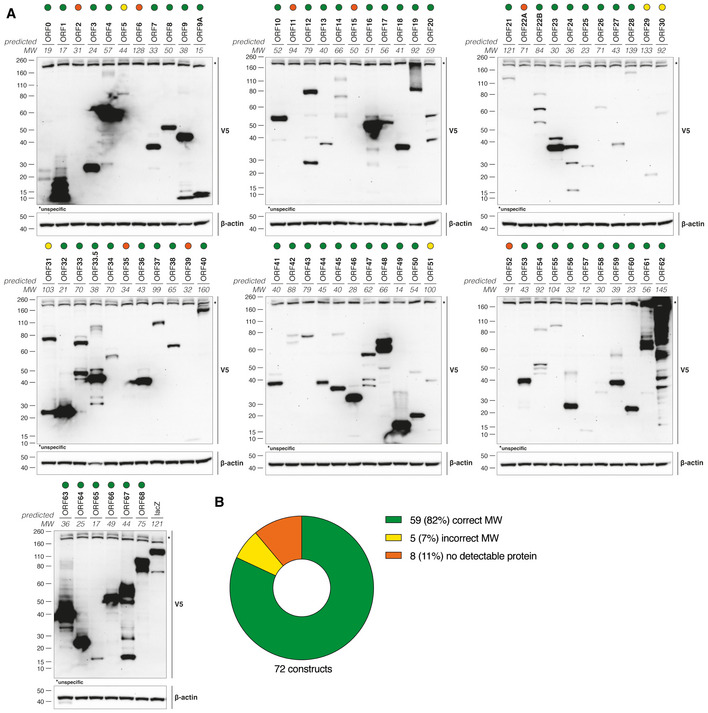
VZV ORF expression library. Related to Fig 2 HEK293T cells were transiently transfected with individual VZV ORF expression constructs. The next day, cell lysates were subjected to immunoblotting. Ectopically expressed proteins were detected with an antibody against the V5 tag. The predicted molecular weight (MW) is indicated in kDa. Coloured circles highlight VZV proteins expressed at the predicted MW (green), expressed at a wrong MW (yellow) or not detectably expressed (orange).Summary of the data in (A). Data information: Data in panels (A) and (B) are from one experiment.

Type I IFNs inhibit VZV infection (Ku *et al*, 2016; Kim *et al*, 2017). VZV, like many other viruses, employs immune evasion strategies that target the type I IFN system. For example, both ORF61 and ORF62 limit IRF3 activation through distinct mechanisms (Sen *et al*, 2010; Zhu *et al*, 2011). In light of our finding that cGAS was crucial for type I IFN induction in response to VZV, we hypothesised that the virus expresses a direct antagonist of cGAS and/or STING. Indeed, other large DNA viruses often encode multiple antagonists of the same innate immune pathway (Smith *et al*, 2018; Stempel *et al*, 2019). In order to test the role of individual viral gene products in immune evasion, we generated an expression library for all canonical VZV ORFs. All coding sequences were PCR‐amplified and cloned into a gateway entry vector. Using recombination, these sequences were then shuttled into a lentiviral vector (pLenti6.3/TO/V5). This vector allows expression with a C‐terminal V5 epitope tag either after transient transfection or *via* lentiviral transduction. To validate these constructs, we transiently transfected HEK293T cells and analysed expression of VZV proteins by immunoblot using an antibody against the V5 tag (Fig EV2). Fifty‐nine of 72 constructs (82%) were expressed, and bands at the expected molecular weights were detected. An additional five constructs were expressed but not at the expected size, and eight were not expressed at detectable levels. This VZV ORF library is a resource for the scientific community and is available to all interested scientists.

### VZV ORF9 is an antagonist of DNA sensing

**Figure 2:**
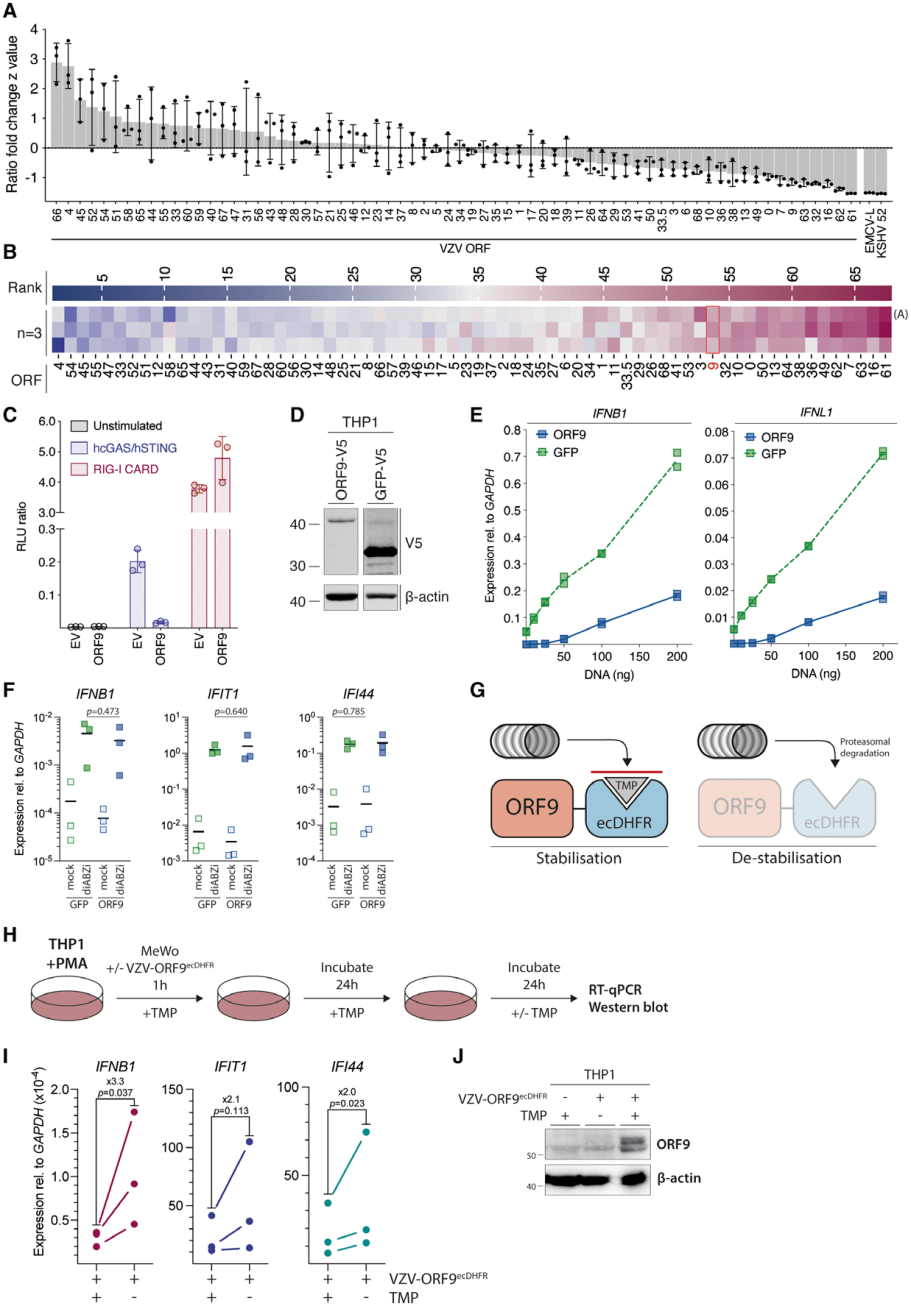
Screening of all VZV ORFs identifies ORF9 as an antagonist of cGAS‐mediated DNA sensing HEK293T cells were transfected with pRL‐TK, p125‐F‐Luc, expression plasmids for human cGAS and human STING, as well as expression plasmids for individual ORFs. The next day, luciferase activity was determined. For each ORF, a z‐value was calculated and ORFs sorted by descending values.The experiment shown in panel (A) was repeated three times, and for each ORF in each experiment, a rank was determined according to *z*‐value (highest *z*‐value = rank 1). Ranks are displayed as a heatmap. The first row shows results displayed in panel (A).HEK293T cells were transfected as in (A, B) with expression constructs for VZV ORF9 or empty vector. In parallel, reporter expression was stimulated by co‐transfection of the RIG‐I‐CARD plasmid instead of hcGAS and hSTING.Western blot analysis of PMA‐differentiated THP1 monocytes stably transduced with either V5‐tagged VZV ORF9 or GFP using the indicated antibodies.THP1 monocytes stably transduced with either VZV ORF9 or GFP were PMA‐differentiated and transfected with indicated doses of dsDNA. Expression of *IFNB1* and *IFNL1* was assessed by RT–qPCR. Graphs show expression relative to *GAPDH*.THP1 monocytes stably transduced with either VZV ORF9 or GFP were treated with the STING agonist diABZi (0.005 µM). Expression of *IFNB1*, *IFIT1*, and *IFI44* was assessed by RT–qPCR. Graphs show expression relative to *GAPDH*. See also Fig EV2A.Schematic detailing the *E*. *coli* DHFR degron system.Schematic detailing the experimental procedure for (I) and (J). See text for details.PMA‐differentiated THP1 cells were co‐cultured with VZV‐ORF9^ecDHFR^‐infected MeWo cells for 1 h in the presence of TMP, washed, and incubated for another 24 h in the presence of TMP. Cells were then washed extensively, and fresh medium was added that was supplemented or not with TMP. Expression levels of *IFNB1*, *IFIT1*, and *IFI44* were measured by RT–qPCR after a further 24 h incubation. Graphs show expression relative to *GAPDH*. Mean fold changes are indicated.Cells infected and treated as in (I) with the addition of uninfected cells were analysed by western blotting using the indicated antibodies. Data information: Panel (A) is representative of three independent biological experiments, where each data point represents a technical replicate (mean ± SD). The three independent experiments are summarised in panel (B), where the data from (A) forms the first row. Panel (C) is representing two independent biological experiments, where each data point represents a technical replicate (mean ± SD). Panels (D) and (E) are representing two independent biological experiments. In (E), each data point represents a technical replicate. Panel (F) shows pooled data from three repeats, where each data point represents an independent biological experiment (horizontal bars show means). Panel (I) shows pooled data from three repeats, where each data point represents an independent biological experiment. Panel (J) is representative of three independent experiments. Statistical analysis in (F) and (I) were paired *t*‐tests. See also Figs EV2 and EV3. Source data are available online for this figure.

To investigate whether VZV ORFs block cGAS/STING activation, we utilised a luciferase‐based screening platform in HEK293T cells. In brief, a plasmid expressing Firefly luciferase under *IFNB1* promoter control and pRL‐TK, which constitutively expresses Renilla luciferase, were transiently transfected. HEK293T cells do not express cGAS and STING naturally. We therefore reconstituted human cGAS and human STING by transient transfection, which results in activation of the *IFNB1* promoter and firefly luciferase expression. Lastly, individual viral ORFs (or controls) were co‐expressed. Firefly luciferase expression was normalised to Renilla luciferase expression, and we calculated for each ORF a luciferase fold change to an empty vector control condition without cGAS and STING expression constructs. The mean and standard deviation of all data points was then used to calculate Z‐values, which represent the number of standard deviations an individual data point is diverging from the mean. We used these Z‐scores to rank ORFs in their ability to block IFN activation downstream of cGAS/STING (Fig 2A). KSHV ORF52, a previously described cGAS antagonist (Wu *et al*, 2015), and the L protein of EMCV, a previously described IRF3‐antagonist (Freundt *et al*, 2018), served as positive controls. As expected, we found these with the lowest ranks (i.e. smallest fold change) in our assay, and both potently blocked firefly luciferase induction. In addition, we identified a number of VZV ORFs that showed similar behaviour. The two aforementioned IRF3 antagonists expressed by VZV, ORF61 and ORF62, were among them, which further validated our approach.

We performed this screening experiment three times; Fig 2B displays the results as a heatmap. We identified a number of ORFs that reproducibly ranked very low, including ORF9. To test if ORF9 specifically blocked cGAS and/or STING, and not downstream signalling proteins such as IRF3 that are also activated by other PRRs, we compared this hit from the primary screen in its ability to block reporter activation by overexpression of cGAS/STING or RIG‐I‐CARD. RIG‐I‐CARD is a constitutively active fragment of RIG‐I that activates the *IFNB1* promoter *via* MAVS. A direct cGAS/STING antagonist is therefore unable to block this stimulation. ORF9 selectively blocked reporter activation by cGAS/STING but not RIG‐I‐CARD when compared to empty vector (Fig 2C).

**Figure EV3:**
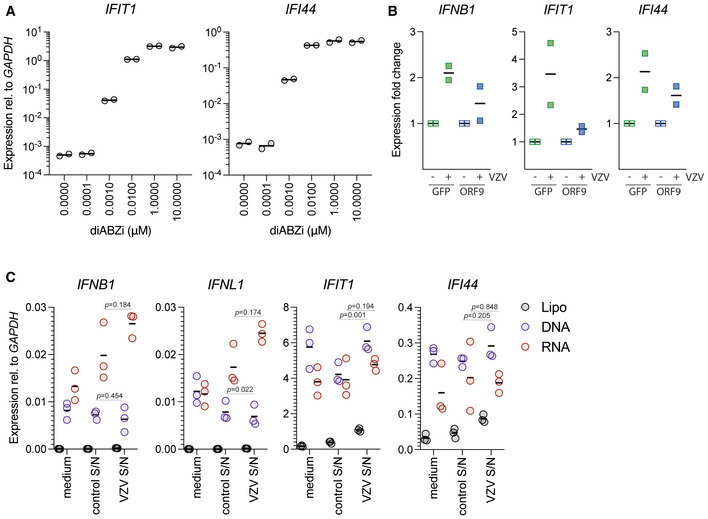
ORF9 blocks IFN and ISG induction in VZV‐infected cells. Related to Fig 2 THP1 cells were treated with the indicated concentrations of diABZi. After 24 h, expression of the indicated ISGs was determined by RT–qPCR. Data are relative to *GAPDH*.THP1 monocytes stably transduced with either VZV ORF9 or GFP were VZV or mock infected by co‐culture with VZV‐infected or uninfected MeWo cells. Expression of *IFNB1*, *IFIT1*, and *IFI44* was assessed by RT–qPCR. Expression fold change relative to uninfected cells is shown.THP1 cells were treated with PMA and were then transfected with *E*. *coli* DNA (DNA), the RIG‐I agonist Neo^1‐99^ *in vitro* transcribed RNA (RNA) or, as control, treated with transfection reagent alone (lipofectamine 2000, Lipo). These stimulations were performed in fresh M10 medium (medium) and in conditioned medium collected from uninfected (control S/N) or VZV‐infected (VZV S/N) MeWo cells. After 24 h, expression of the indicated IFNs and ISGs was determined by RT–qPCR. Data are relative to *GAPDH*. Data information: Data in (A) are technical duplicates from a single experiment (horizontal bars show means). Panel (B) shows pooled data from two repeats, where each data point represents an independent biological experiment (horizontal bars show means). In (C), data are pooled from three biological repeat experiments each performed in technical duplicate (horizontal bars show means). Statistical analysis was paired *t*‐tests.

We then aimed to verify that VZV ORF9 antagonises activation of cGAS by dsDNA in an endogenous setting. THP1 monocytes were stably transduced with ORF9‐V5 or GFP‐V5 as a negative control (Fig 2D). We stimulated cGAS in these cells by transfection of increasing doses of dsDNA and measured expression levels of *IFNB1* mRNA and *IFNL1* mRNA (encoding a type III IFN) by RT–qPCR (Fig 2E). As expected, THP1 cells expressing GFP showed a dose‐dependent increase in expression of both transcripts. In contrast, cells expressing ORF9 did not respond to low doses of DNA. At higher doses, their response was attenuated when compared to GFP‐expressing cells. To test whether ORF9 inhibits cGAS or STING, we used the small molecule STING agonist diABZi to stimulate STING signalling independently of cGAS (Ramanjulu *et al*, 2018). Based on a dose titration (Fig EV3A), we added a non‐saturating dose (0.005 µM) of diABZi to THP1 cells transduced with ORF9 or GFP as control. The expression of *IFNB1* and two ISGs was stimulated to a similar degree in cells expressing ORF9 or GFP (Fig 2F). These data show that STING signalling is unaffected in ORF9‐expressing cells, indicating that ORF9 acts on cGAS directly. We further investigated how ORF9 affects the type I IFN response in THP1 cells upon VZV infection. THP1 cells expressing ORF9 or GFP were infected by co‐culture with VZV‐infected MeWo cells, and the type I IFN response was measured by RT–qPCR. The presence of ectopically expressed ORF9 attenuated the upregulation of *IFNB1* and ISGs (Fig EV3B). This effect was not simply due to a soluble factor released by VZV‐infected MeWo cells: the response of THP1 cells to transfected immunostimulatory nucleic acids was largely unaffected by supernatant samples from VZV‐infected cells (Fig EV3C).

Lastly, we aimed to investigate the antagonist function of endogenous ORF9 expressed during viral infection. Since ORF9 is essential for viral replication, we used a recombinant virus in which a degron motif is fused to the ORF9 protein (VZV‐ORF9^ecDHFR^; Warner *et al*, 2021). In this setting, the stability of the degron (and the ORF9 protein fused to it) in cells can be regulated with the degron ligand trimethoprim (TMP) (Fig 2G). Withdrawal of TMP leads to destabilisation of the degron and subsequent proteasomal degradation of the ORF9‐degron protein. We hypothesised that upon TMP withdrawal, ORF9 degradation leads to an increased type I IFN response in VZV‐ORF9^ecDHFR^‐infected THP1 cells. To test this, PMA‐differentiated THP1 cells were co‐cultured in the presence of TMP with VZV‐ORF9^ecDHFR^‐infected MeWo cells for 1 h (Fig 2H). The cells were then washed of inoculum and cultured with TMP for an additional 24 h. Finally, the cells were washed free of TMP, cultured overnight in the presence or absence of TMP, and harvested for RT–qPCR and immunoblot analysis. Expression of *IFNB1* and ISGs was increased in cells where TMP was withdrawn (Fig 2I). The absence of ORF9 protein in cells cultured without TMP was confirmed by immunoblotting (Fig 2J). These data show that the absence of endogenously expressed ORF9 during VZV infection leads to an increased type I IFN response in THP1 cells. This confirms a role of ORF9 in inhibiting the cGAS‐dependent response to VZV infection in these cells. Collectively, the data presented in Fig 2 revealed that ectopically and endogenously expressed VZV ORF9 protein prevented cGAS activation.

### ORF9 interacts with cGAS

**Figure 3:**
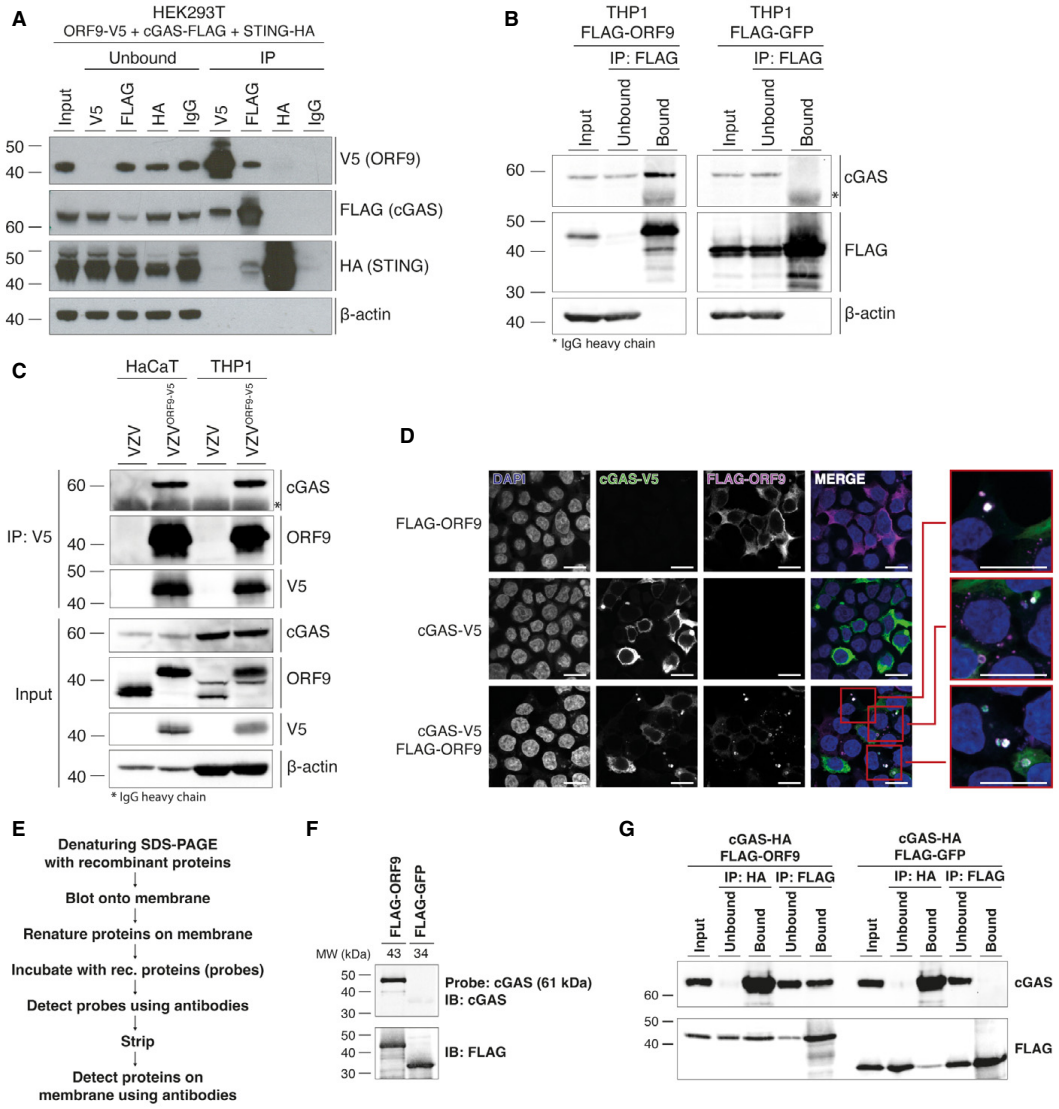
ORF9 interacts with cGAS HEK293T cells were transfected with expression plasmids for ORF9‐V5, human cGAS‐FLAG, and human STING‐HA. The next day, cells were lysed, and overexpressed proteins were immunoprecipitated with α‐V5, α‐FLAG, α‐HA, or IgG isotype control antibody. Input, unbound, and IP fractions were subjected to immunoblotting using the indicated antibodies.THP1 monocytes stably transduced with either VZV FLAG‐ORF9 or FLAG‐GFP were PMA‐differentiated overnight. The next day, cells were lysed and ectopically expressed proteins were immunoprecipitated using α‐FLAG antibody. Input, unbound, and IP fractions were subjected to immunoblotting using the indicated antibodies.HaCaT cells and PMA‐differentiated THP1 cells were infected with WT VZV or VZV^ORF9‐V5^ through co‐culture with infected MeWo cells for 48 h. Cells were lysed and ORF9 was immunoprecipitated using α‐V5 antibody. Input and IP fractions were subjected to immunoblotting using the indicated antibodies.HEK293T cells were seeded onto glass coverslips and were transfected with human cGAS‐V5, FLAG‐ORF9, or both together. The next day, cells were fixed, permeabilised and stained using α‐V5‐FITC, rabbit‐α‐FLAG, and goat‐α‐rabbit‐AF647 antibodies, and DAPI. Mounted coverslips were imaged using confocal microscopy. Scale bars: 15 µm.Outline of the far western protocol.Far western analysis of cGAS‐ORF9 interaction. Recombinant FLAG‐ORF9 and FLAG‐GFP protein were run on an SDS–PAGE gel and transferred to a membrane. After renaturation of proteins, the membrane was incubated with recombinant human cGAS as a probe, which was detected using α‐cGAS antibody. After stripping, the proteins on the membrane were detected using α‐FLAG antibody.Recombinant human cGAS‐HA was mixed with recombinant FLAG‐ORF9 or FLAG‐GFP. Proteins were immunoprecipitated using α‐HA and α‐FLAG antibodies. Input, unbound, and IP fractions were analysed by immunoblotting. Data information: Recombinant proteins used in (F) and (G) are shown in Appendix Fig S4A. Panels (A) and (G) are representative of two independent experiments. Panels (B), (D), and (F) are representative of three independent experiments. Panel (C) is representative of two (HaCaT) and three (THP1) independent experiments. Source data are available online for this figure.

We hypothesised that VZV ORF9 exerts its antagonistic function by directly interacting with cGAS. To test this, HEK293T cells were transiently transfected with expression constructs for cGAS‐FLAG, STING‐HA, and ORF9‐V5, and protein interaction was analysed by immunoprecipitation (IP). We used antibodies against the epitope tags and analysed IP fractions by immunoblotting (Fig 3A). All ectopically expressed proteins were precipitated efficiently, and an IgG isotype control antibody did not precipitate any of the proteins tested. Interestingly, ORF9 was detected in the bound fraction after cGAS IP. The reverse IP confirmed this result: we found cGAS in the ORF9 IP. In contrast, ORF9 did not interact with STING in this assay (Fig 3A). To verify that this interaction occurred with endogenous cGAS, we used THP1 cells stably transduced to express FLAG‐ORF9, which was precipitated from cell lysates with α‐FLAG antibody. Indeed, endogenous cGAS was present in the IP fraction; IP of FLAG‐GFP served as a negative control (Fig 3B).

Next, we asked whether ORF9 expressed from its endogenous promoter during viral infection had the ability to interact with cGAS. We used a recombinant VZV that expressed C‐terminally V5‐tagged ORF9 from its endogenous genomic locus. We infected THP1 cells and HaCaT cells (a keratinocyte cell line that expresses cGAS) with WT VZV or VZV^ORF9‐V5^ and performed α‐V5 IP (Fig 3C). In both THP1 and HaCaT cells infected with VZV^ORF9‐V5^, endogenous cGAS co‐precipitated with ORF9. These data showed that endogenous ORF9 protein expressed by the virus in infected cells interacted with cGAS.

To investigate whether the two proteins co‐localise in cells, we overexpressed tagged ORF9 and cGAS in HEK293T cells and performed immunofluorescence analysis (Fig 3D). Expression of ORF9 alone resulted in cytoplasmic staining. Similarly, cGAS was detected in the cytoplasm. We further observed cGAS foci co‐localising with extranuclear DNA. Extranuclear DNA foci, sometimes in the form of micronuclei, can be observed in some cancer cells and have been shown to bind cGAS (Harding *et al*, 2017; Mackenzie *et al*, 2017; Hu *et al*, 2019). Interestingly, when ORF9 and cGAS were expressed together, both proteins co‐localised in DAPI‐positive, extranuclear regions (Fig 3D). This indicated that ORF9 interacted with cGAS in cells and localised together with cGAS in DNA‐positive areas.

In order to biochemically characterise the interaction between ORF9 and cGAS in more detail, we performed experiments in a cell‐free system. We expressed cGAS, cGAS‐HA, FLAG‐ORF9, and FLAG‐GFP in *Escherichia coli* and performed single‐step purification (Appendix Fig S4A). First, we tested whether ORF9 and cGAS interacted directly using the far western protocol (Fig 3E). FLAG‐ORF9 or FLAG‐GFP protein were separated on a denaturing SDS–PAGE gel and transferred to a membrane. The proteins on the membrane were then re‐natured and incubated with recombinant cGAS as a probe. Binding of cGAS to proteins on the membrane was then tested using α‐cGAS antibody. Indeed, we found that probing for cGAS resulted in a signal at the size of ORF9 (Fig 3F). Importantly, cGAS did not bind to GFP.

We then tested interaction of the two recombinant proteins using immunoprecipitation. FLAG‐ORF9 or FLAG‐GFP was incubated with cGAS‐HA in the test tube. The proteins were then precipitated using antibodies against the epitope tags. Immunoblot analysis of the IP fractions showed that cGAS was co‐immunoprecipitated with ORF9 but not GFP (Fig 3G). The reverse IP of cGAS resulted in binding of both ORF9 and GFP; however, the signal was stronger for ORF9. Taken together, these data indicate that VZV ORF9 and cGAS interacted without the requirement for another cellular or viral protein.

**Figure 4:**
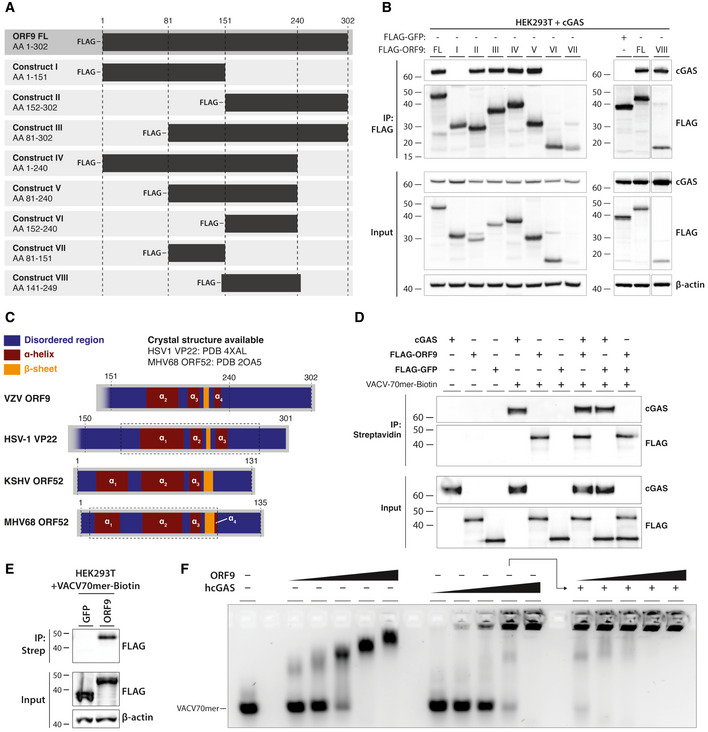
VZV ORF9 binds DNA Schematic detailing ORF9 truncation mutants used in (B).HEK293T cells were transfected with expression plasmids for human cGAS and ORF9 truncation mutants. The next day, cells were lysed and ORF9 proteins were immunoprecipitated using α‐FLAG antibody. IP fractions were subjected to immunoblotting using the indicated antibodies.Schematic detailing predicted structural features of VZV ORF9 in relation to predicted and crystal structure‐derived features in HSV‐1 VP22, KSHV ORF52, and MHV68 ORF52. See text for details.Recombinant cGAS, FLAG‐ORF9, FLAG‐GFP, and biotinylated VACV70mer dsDNA were incubated in the indicated combinations. Recombinant proteins were precipitated using streptavidin beads. Fractions were analysed by immunoblotting.HEK293T cells were transfected with expression plasmids for FLAG‐GFP or FLAG‐ORF9. The next day, cells were lysed and the lysate was spiked with biotinylated VACV70mer dsDNA. DNA was precipitated using streptavidin beads and fractions were subjected to immunoblotting using the indicated antibodies.VACV70mer dsDNA was incubated with indicated proteins and analysed by agarose gel EMSA. Triangles indicate concentrations of ORF9 (0.7, 1.4, 2.9, 5.7, 8.6 µM), cGAS (0.6, 1.1, 2.2, 4.5, 6.7 µM), and ORF9 (as before) in the presence of 4.5 µM cGAS. Data information: Recombinant proteins used in (D) and (F) are described in Appendix Fig S4A and B, respectively. Results shown in panels (B) and (D–F) are representative of two independent experiments. Source data are available online for this figure.

To characterise the interaction of ORF9 and cGAS mechanistically, we constructed ORF9 truncation mutants (Fig 4A). We tested their ability to interact with cGAS by co‐IP after overexpression in HEK293T cells (Fig 4B). Consistent with our earlier observation, full‐length ORF9 co‐immunoprecipitated cGAS. The C‐terminal half of ORF9 (construct II) behaved the same, whilst the N‐terminal half (construct I) failed to interact with cGAS. ORF9 constructs III, IV, and V also pulled down cGAS. All ORF9 constructs that interacted with cGAS shared amino acids (AA) 151 to 240. However, the IP of this region in isolation (ORF9^151‐240^, construct VI) did not co‐precipitate cGAS (Fig 4B). Extension of this construct at both ends by about 10 amino acids to generate construct VIII (AA 141–249) restored robust interaction with cGAS (Fig 4B).

### ORF9 binds DNA

To gain insight into possible structural features of ORF9 in this region, we used the PSIPRED 4.0 algorithm to predict its secondary structure based on the AA sequence (Buchan & Jones, 2019). This analysis predicted a two helix–sheet–helix motif in the C‐terminal region of ORF9 (Fig 4C). We obtained a similar secondary structure prediction for the C‐terminal half of VP22, the HSV‐1 homologue of VZV ORF9. A crystal structure is available for this region of VP22 (Hew *et al*, 2015) and confirms the presence of the predicted two helix–sheet–helix motif. Hew *et al* further identified a structural similarity of VP22 with the unrelated ORF52 protein of murine herpesvirus 68 (MHV68). MHV68 is closely related to human herpesvirus 8, also known as Kaposi sarcoma‐associated herpesvirus (KSHV). KSHV ORF52 has been identified as a cGAS antagonist and has DNA‐binding properties (Wu *et al*, 2015). This led us to hypothesise that ORF9 interacts with DNA. To test this, we incubated biotinylated VACV‐70mer dsDNA with recombinant ORF9; recombinant cGAS and GFP served as positive and negative controls, respectively. The VACV‐70mer is a well‐established immunostimulatory dsDNA that binds cGAS (Unterholzner *et al*, 2010; Almine *et al*, 2017; Lum *et al*, 2018). The DNA was then precipitated using streptavidin beads, and the presence of bound proteins was analysed by immunoblotting (Fig 4D and Appendix Fig S4A). In the absence of DNA, none of the proteins were precipitated. As expected, cGAS bound to DNA. Interestingly, ORF9 was also pulled down by DNA, both alone and in the presence of cGAS. GFP did not bind DNA under any conditions (Fig 4D). We further overexpressed either FLAG‐ORF9 or FLAG‐GFP in HEK293T cells and performed a similar precipitation experiment after spiking the lysates from these cells with VACV70mer‐Biotin (Fig 4E). As expected, ORF9, but not GFP, was pulled down with DNA. To characterise the interaction of ORF9 and DNA in more detail, we performed agarose gel electromobility shift assays (EMSA; Fig 4F and Appendix Fig S4B). Recombinant ORF9 protein impaired the mobility of VACV70mer dsDNA, indicating the formation of ORF9‐DNA complexes. These increased in size with higher doses of ORF9, suggesting multivalent protein–protein/protein–DNA interactions. We performed similar experiments with full‐length human cGAS protein. As previously described (Zhou *et al*, 2018), hcGAS and DNA form high‐molecular‐weight (HMW) complexes, which are unable to migrate out of the gel pocket. Next, we tested incubation of VACV70mer and cGAS with increasing doses of ORF9 protein. Addition of ORF9 to cGAS and DNA increased the size of protein/DNA complexes observed at this concentration of cGAS alone (Fig 4F). Taken together, these data show that ORF9 interacted with both DNA and cGAS, without displacing cGAS from DNA.

### ORF9 phase‐separates with DNA

**Figure 5:**
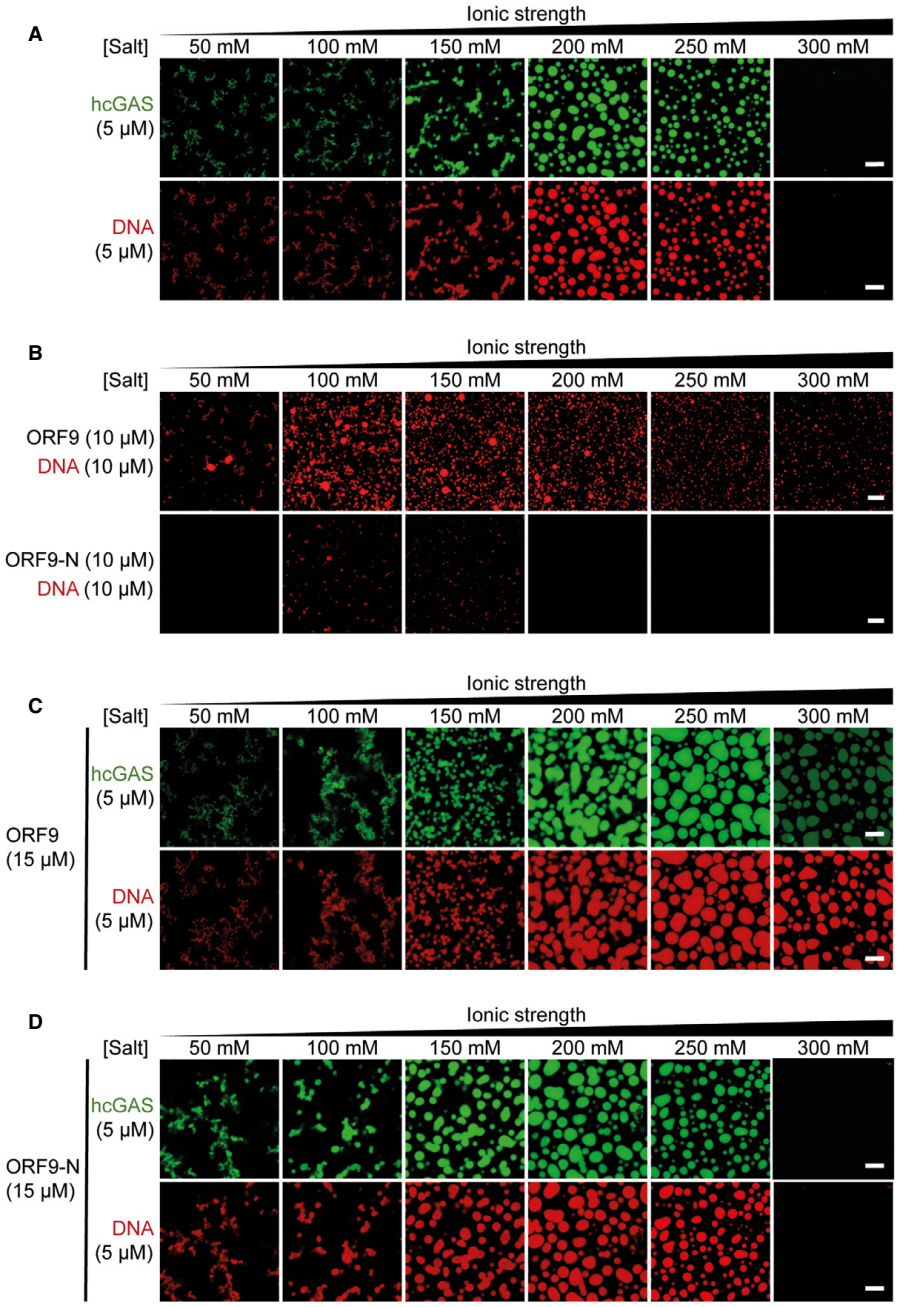
ORF9 phase‐separates with DNA A5 µM hcGAS (with 10% AlexaFluor‐488‐labelled) was incubated with 5 µM dsDNA‐100 (with 10% of DNA Cy3‐labelled) with increasing salt concentrations (total NaCl and KCl) and imaged by confocal microscopy.B10 µM ORF9 or ORF9‐N were incubated with 10 µM dsDNA‐100 (with 10% of DNA Cy3‐labelled) and analysed as in (A).C, D5 µM hcGAS (with 10% AlexaFluor‐488‐labelled) was incubated with 5 µM dsDNA‐100 (with 10% of DNA Cy3‐labelled) and 15 µM ORF9 (C) or ORF9‐N (D) and analysed as in (A). Data information: Recombinant proteins used in are described in Appendix Fig S4C. Scale bar, 10 µm. Data are representative of 3 experiments. See also Fig EV4.

**Figure EV4:**
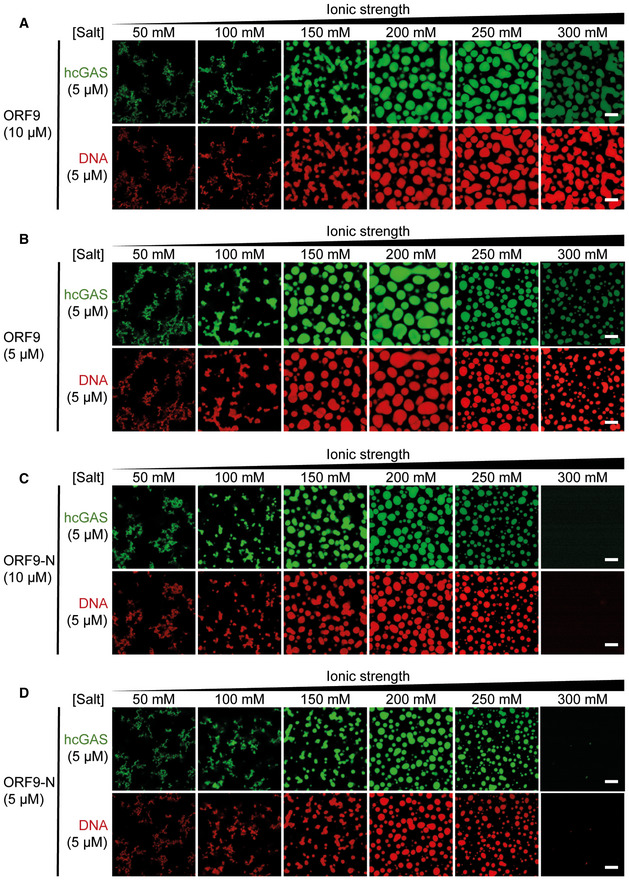
ORF9 phase‐separates with DNA. Related to Fig 5 A–DPhase separation of cGAS and DNA was analysed as described in Fig 5C and D using 10 µM or 5 µM ORF9 or ORF9‐N. Data information: Data are representative of three experiments.

Liquid–liquid phase separation contributes to cGAS activation by dsDNA (Du & Chen, 2018; Xie *et al*, 2019; Zhou *et al*, 2021). This is driven by multivalent interactions between cGAS and DNA. In light of our results that ORF9 bound cGAS and DNA, we investigated the effect of ORF9 on cGAS–DNA phase separation. As reported previously, we observed droplet formation by human cGAS and labelled dsDNA, which was sensitive to increasing salt concentration > 250 mM (Fig 5A and Appendix Fig S4C). Similarly, ORF9 and labelled dsDNA formed liquid droplets in the absence of cGAS (Fig 5B and Appendix Fig S4C). ORF9‐DNA droplets were smaller than cGAS‐DNA droplets, which may indicate a lower propensity of ORF9 to phase‐separate with DNA compared to cGAS. A C‐terminally truncated version of ORF9 (ORF9‐N, AA 1–244) had much reduced ability to form liquid droplets (Fig 5B and Appendix Fig S4C). Next, we formed cGAS–DNA droplets in the presence of ORF9. Larger droplets were formed by cGAS and DNA when a three‐fold molar excess of full‐length ORF9, but not ORF9‐N, was added (Fig 5C and D). This was consistent with our EMSA data (Fig 4F). Interestingly, at 300 mM NaCl/KCl, DNA and cGAS alone did not phase‐separate, but droplets containing DNA and cGAS were formed at this salt concentration in the presence of ORF9 (Fig 5A and C). Furthermore, at 300 mM NaCl/KCl, the intensity of the cGAS signal, but not of the DNA signal, was diminished compared to lower salt concentrations. These observations were reproducible using lower ORF9:cGAS molar ratios (Fig EV4). One of the multiple possible interpretations is that ORF9 displaces cGAS from DNA under these conditions, which is consistent with a recent report (Xu *et al*, 2021).

### VZV ORF9 Inhibits cGAMP synthesis

**Figure 6:**
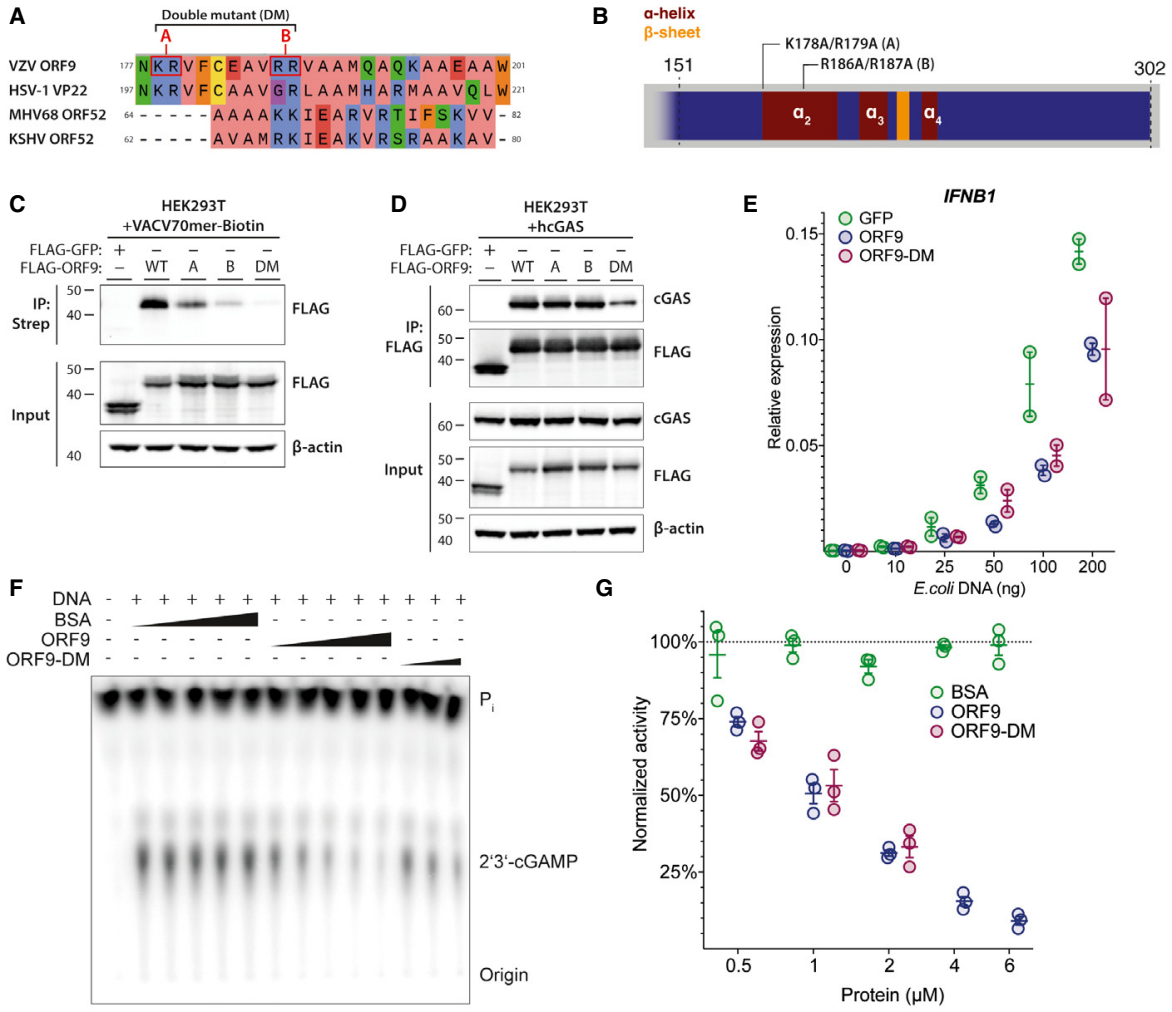
ORF9 inhibits cGAS’ catalytic activity Protein sequence alignment of VZV ORF9, HSV‐1 VP22, KSHV ORF52, and MHV68 ORF52. Residues selected for mutagenesis in ORF9 are highlighted in red. Residue colouring represents physico‐chemical properties (green: hydrophilic, blue: positive, coral: aliphatic/hydrophilic, orange: aromatic, yellow: cysteine, red: negative, purple: conformationally special). See text for details.Schematic detailing position and identity of residues mutated in (C–G).HEK293T cells were transfected with expression plasmids for FLAG‐GFP or FLAG‐ORF9 as indicated. The next day, cells were lysed and the lysate was spiked with biotinylated VACV70mer dsDNA. DNA was precipitated using streptavidin beads and proteins were analysed by immunoblotting using the indicated antibodies.HEK293T cells were transfected with expression plasmids for human cGAS and FLAG‐GFP or FLAG‐ORF9. The next day, cells were lysed and proteins were immunoprecipitated using α‐FLAG antibody and analysed by immunoblotting using the indicated antibodies.THP1 cells stably transduced with GFP, ORF9, or ORF9‐DM were transfected with the indicated amounts of *E*. *coli* dsDNA. The next day, *IFNB1* expression was assessed by RT–qPCR.
*In vitro* cGAS activity assay. Recombinant hcGAS was incubated with ATP, GTP, and radioactive α32P‐ATP at 37°C with addition of dsDNA and other proteins as indicated (triangles represent concentrations of 0.5, 1, 2, 4, 6 µM). ORF9‐DM was tested only at 0.5, 1, 2 µM. Reactions were treated with calf‐intestinal phosphatase, and products were analysed by thin‐layer chromatography and phosphorimaging.Signal intensities from (F) were determined by densitometry analysis and normalised to the average BSA signal. Data information: Recombinant proteins used in (F) and (G) are described in Appendix Fig S4B. Data shown in panels (C) and (D) are representative of two independent experiments. Panel (E) shows pooled data from two repeats, where each data point represents an independent biological experiment (*n* = 2 ± range). Panel (F) is representative of three independent experiments. Panel (G) shows pooled data from three repeats, where each data point represents an independent biological experiment (*n* = 3 ± SEM). Source data are available online for this figure.

We next sought to establish the importance of DNA binding for ORF9’s function as a cGAS antagonist. Alignment of ORF9 with related herpesvirus proteins (Fig 4C) revealed multiple conserved, positively charged residues (Fig 6A). This included ORF9 K178/R179 and ORF9 R186/R187. The latter is aligned with KSHV ORF52 R68/K69 that are required for DNA binding (Wu *et al*, 2015). We therefore generated three ORF9 mutants: K178A/R179A (construct A), R186A/R187A (construct B), and a double mutant (DM), in which all four residues were mutated to alanine. The mutation sites A and B are located at the beginning and in the middle, respectively, of the first alpha helix in the predicted two helix–sheet–helix of ORF9 (Fig 6B). In the HSV‐1 VP22 crystal structure, the corresponding helix forms part of a large, positively charged groove, consistent with a possible role in DNA binding (Hew *et al*, 2015). To test the effect of these mutations experimentally, we performed DNA pull‐down experiments. As observed before (Fig 4E), WT ORF9 robustly precipitated with DNA (Fig 6C). In contrast, both the A and B mutants were impaired in their ability to bind DNA, with a stronger effect for the B site. The double mutant did not detectably interact with DNA. Next, we investigated whether the mutant ORF9 proteins interacted with cGAS (Fig 6D). Mutation of either the A or B site alone had no effect on cGAS binding, but ORF9‐DM showed attenuated cGAS binding.

To test the ORF9‐DM protein functionally, we generated THP1 cell lines that stably overexpressed GFP, ORF9, or ORF9‐DM and stimulated these cells with increasing doses of dsDNA (Fig 6E). Compared to GFP‐expressing cells, we found equally reduced *IFNB1* induction in cells expressing either WT or mutant ORF9. Taken together, these results indicate that the interaction of ORF9 and cGAS was partially dependent on ORF9’s ability to bind DNA. Intriguingly, however, DNA binding by ORF9 was not required for its ability to inhibit DNA sensing.

Lastly, we used a cell‐free cGAMP synthesis assay (Kranzusch *et al*, 2013) to test whether ORF9 inhibits cGAS enzymatic activity directly. For this, recombinant human cGAS was incubated with radioactively labelled ATP, GTP, DNA, and recombinant ORF9 or bovine serum albumin (BSA) as control. The radioactively labelled 2′3′‐cGAMP produced in these reactions was visualised by thin‐layer chromatography and phosphorimaging (Fig 6F and Appendix Fig S4B). Whilst addition of BSA did not affect cGAMP production at any dose, ORF9 inhibited cGAS activity in a dose‐dependent manner (Fig 6F and G). At the doses tested, ORF9‐DM was equally potent in inhibiting cGAS compared to WT ORF9. In sum, these results showed that ORF9 directly inhibited the enzymatic activity of cGAS by a mechanism that did not require DNA binding by ORF9.

## Discussion

Varicella‐Zoster virus is a highly prevalent human virus, yet little is known about its host–pathogen interactions and innate immunity. Here we report that cGAS and its downstream signalling pathway consisting of STING, TBK1, and IRF3 were required for type I IFN induction after VZV infection in THP1 cells. An earlier study using RNA interference had implicated STING in innate recognition of VZV (Kim *et al*, 2017). Our results confirm this observation by complete genetic ablation, and—importantly—identify cGAS as the DNA sensor for VZV. It is possible that innate sensing pathways that detect VZV differ between cell types. Indeed, a study using inhibitory synthetic oligonucleotides found that plasmacytoid DCs produce type I IFN in a partially TLR9‐dependent manner (Yu *et al*, 2011). In contrast, we found that the essential TLR9 adaptor protein MyD88 was dispensable for type I IFN induction in VZV‐infected THP1 cells. Of note, the inhibitory ODN used in the aforementioned study was later shown to exert inhibitory activity on cGAS as well (Steinhagen *et al*, 2018), thus complicating interpretation of those results. It will be interesting to characterise innate sensing pathways in different cell types relevant to *in vivo* infection, including neuronal cells, T cells, and skin cells. This is important because the viral life cycle and effects of viral replication on host cells can differ between cell types (Zerboni *et al*, 2014). For example, VZV shows cytopathic effects in fibroblast and some immune cells but does not induce cell death in neurons (Gerada *et al*, 2020). Future investigation should therefore address the role of PRRs not only in the induction of cytokines such as type I IFNs but also in the induction of cell death (Maelfait *et al*, 2020).

Our identification of cGAS as a sensor of VZV infection implicates recognition of an immunostimulatory DNA in infected cells. Studies of herpesvirus entry suggest that the viral DNA remains within the viral capsid during nuclear targeting and may therefore be unavailable for binding to the cytosolic pool of cGAS (Radtke *et al*, 2006). Single‐cell analysis of HSV‐1 infection showed that only cells undergoing abortive infection respond by production of type I IFNs (Drayman *et al*, 2019). It is therefore possible that viral particles with defective capsids are responsible for the type I IFN response observed in bulk populations of cells. Furthermore, cellular restriction mechanisms may make viral DNA from capsids accessible for cGAS binding. Indeed, degradation of herpesviral capsids *via* the ubiquitin–proteasome system has been suggested to release viral DNA for recognition (Horan *et al*, 2013). Alternatively, cellular DNA may induce type I IFN. Indeed, infections with multiple different viruses result in the accumulation of extranuclear DNA and in mitochondrial damage (Ablasser & Chen, 2019). Host DNA has been shown to stimulate cGAS in the context of HSV‐1 infection (West *et al*, 2015). Whether viral and/or cellular DNA species activate cGAS in VZV‐infected cells should be tested in the future by deep sequencing of cGAS‐bound DNA. However, this approach has thus far been hampered by the lack of suitable antibodies for specific cGAS IP.

We further describe the identification of ORF9 as a cGAS antagonist. ORF9 interacted with cGAS in a variety of assays, and inhibited cGAMP production and DNA‐triggered type I IFN induction. ORF9 is a tegument protein, making it an attractive candidate for antagonising innate immunity. Tegument proteins are contained within viral particles and are delivered into cells at the same time as viral DNA. ORF9 may therefore limit DNA sensing during early infection before viral gene expression. At first glance, our findings that cGAS recognises VZV infection and that ORF9 inhibits cGAS activation might appear contradictory. However, viral immune evasion mechanisms typically limit but not entirely suppress innate immune responses. This notion is supported by the observation that in some cases multiple viral antagonists target the same host defence pathway (Smith *et al*, 2018; Stempel *et al*, 2019). In addition, cGAS activation may occur partly in cells infected with defective virions (Drayman *et al*, 2019) that are likely to contain and/or express reduced amounts of ORF9.

What is the molecular mechanism by which ORF9 inhibits cGAS? Formation of multimeric complexes and higher order structures of cGAS and DNA facilitate cGAS activation (Li *et al*, 2013; Zhang *et al*, 2014; Andreeva *et al*, 2017; Du & Chen, 2018; Zhou *et al*, 2018). We found that ORF9 binds DNA and phase‐separated with DNA. We therefore speculated that ORF9 might disrupt cGAS–DNA oligomers. However, further gel shift and phase separation experiments revealed a more nuanced situation. At salt concentrations up to 250 mM NaCl/KCl, ORF9‐facilitated phase separation and DNA–cGAS complexes migrated more slowly in EMSA when ORF9 was added. Nonetheless, at 300 mM salt, ORF9 appeared to limit the presence cGAS in condensates. Similarly, Xu *et al* (2021) recently reported that ORF9 displaces cGAS from cGAS–DNA droplets. It is noteworthy that these experiments were performed at ~200 mM NaCl, a salt concentration at which we did not observe cGAS displacement by ORF9. It is likely that these differences can be explained by the experimental setup, which involved simultaneous mixing of DNA, cGAS, and ORF9 in our experiments and preformed DNA–cGAS complexes with subsequent addition of ORF9 in Xu *et al* (2021). Other parameters such as DNA length are likely important, too. An ORF9 mutant with abrogated DNA binding retained the ability to bind cGAS and to inhibit cGAMP production *in vitro* and IFNβ induction *in cellulo*. Although we cannot exclude that this mutant retained weak DNA binding below the sensitivity of our assays, we prefer a model in which direct protein–protein interactions between ORF9 and cGAS are important for inhibition of cGAMP production. DNA binding of ORF9 may have unrelated functions and/or may facilitate cGAS interaction and inhibition in specific settings. Future experiments are required to decipher in detail the relationships between ORF9, cGAS, DNA binding, and cGAMP production. This could include testing DNA length requirements as cGAS oligomer formation is dependent on DNA length (Li *et al*, 2013; Andreeva *et al*, 2017; Luecke *et al*, 2017; Zhou *et al*, 2018).

ORF9 is essential for viral replication and is a member of the α‐herpesvirus UL49 gene family (Tischer *et al*, 2007; Che *et al*, 2008). With its closest relative, HSV‐1 VP22, it shares 36% AA sequence similarity (Hew *et al*, 2015). ORF9 has well‐established roles in VZV nuclear egress and secondary envelopment (Che *et al*, 2013; Riva *et al*, 2013, 2015; Lebrun *et al*, 2018). Mutational analyses have attributed these functions to AAs or regions either in the N‐terminal half or the extreme C‐terminus of the protein, whilst we describe a central region (AA151–240) to be required for the interaction of ORF9 with cGAS. A crystal structure is available for the core domain of HSV‐1 VP22 that is homologous to this region of ORF9 (Hew *et al*, 2015). The HSV‐1 VP22 core folds into a two helix–sheet–helix motif. Hew *et al* further describe the structural similarity between HSV1 VP22 and the unrelated MHV68 ORF52 (Hew *et al*, 2015). MHV68 ORF52 is the homologue of KSHV ORF52. Secondary structure prediction and examination of the published crystal structures revealed that VZV ORF9, HSV1 VP22, KSHV ORF52, and MHV68 ORF52 all potentially share a two helix–sheet structural feature (Fig 4C). Interestingly, both HSV‐1 VP22 and KSHV ORF52 have been described previously to inhibit cGAS activation (Wu *et al*, 2015; Huang *et al*, 2018). Antagonism of cGAS by KSHV ORF52 requires its DNA‐binding properties (Wu *et al*, 2015). In contrast, we found that ORF9’s DNA‐binding ability was not required for inhibition of cGAS. This indicates that whilst structural similarity between the aforementioned proteins could confer cGAS inhibitory properties, the precise molecular mechanisms might differ. In addition, VZV ORF9 and KSHV ORF52 are essential viral gene products whilst HSV‐1 VP22 is not required for viral replication (Li *et al*, 2016; Huang *et al*, 2018). Future experiment will be required to shed light on the precise relationship between these viral proteins.

Collectively, our observations lead us to propose a model in which distantly related herpesviruses have retained within unrelated proteins a structural feature that confers cGAS antagonist properties. Alpha, beta, and gamma‐herpesviruses have been estimated to have evolutionarily diverged hundreds of millions of years ago (McGeoch *et al*, 1995; Davison, 2002; preprint: Brito & Pinney, 2018). Anemone species that have diverged from humans more than 500 million years ago harbour cGAS‐like enzymes (Kranzusch *et al*, 2015). This indicates that a common ancestral organism expressed such proteins. It further opens up the possibility that ancient herpes viruses evolved the relevant evasion strategies. We hypothesise that antagonism of cGAS by herpesviruses constitutes an ancient molecular mechanism that evolved long before the advent of antiviral cytokines and IRF3 during evolution.

## Materials and Methods

### Reagents and Tools table

**Table:** 

Reagent/Resource	Reference or Source	Identifier or Catalog Number
**Experimental Models**		
HaCaT	Gift from Leonie Unterholzner (Lancaster University, UK)	RRID:CVCL_0038
HEK293	Gift from Caetano Reis e Sousa (The Francis Crick Institute, UK)	RRID:CVCL_0045
HEK293‐ISRE‐Fluc	Generated previously in our laboratory (Bridgeman *et al*, 2015)	N/A
HEK293T	Gift from Caetano Reis e Sousa (The Francis Crick Institute, UK)	RRID:CVCL_0063
MeWo	Gift from Graham Ogg (University of Oxford, UK)	RRID:CVCL_0445
THP1 cGAS KO	This study	N/A
THP1 Dual IFNAR2‐KO	Invivogen	Cat. #thpd‐koifnar2
THP1 Dual MyD88‐KO	Invivogen	Cat. #thpd‐komyd
THP1 Dual WT	Invivogen	Cat. #thpd‐nfis
THP1 IRF3 KO	This study	N/A
THP1 MAVS KO	This study	N/A
THP1 STING KO	Gift from Soren Paludan (Aarhus University, Denmark) (Holm *et al*, 2016)	N/A
THP1 TBK1 KO	Gift from Soren Paludan (Aarhus University, Denmark) (Holm *et al*, 2016)	N/A
VZV rOka	Gift from Jeffrey Cohen (NIH, Bethesda, USA) (Cohen & Seidel, 1993)	N/A
VZV‐ORF9^ecDHFR^	Gift from Paul R. Kinchington (University of Pittsburgh, USA) (Warner *et al*, 2021)	N/A
VZV^ORF9‐V5^	Gift from Catherine Sadzot (University of Liege, Belgium) (Riva *et al*, 2013)	N/A
**Recombinant DNA**		
hRIG‐I‐CARD	Gift from Andreas Pichlmair (Technical University Munich, Germany)	N/A
p125‐Luc	Gift from T. Fujita (Kyoto University, Japan) (Yoneyama *et al*, 2004)	N/A
p8.91	Gift from Greg Towers (University College London)	N/A
pcDNA/FLAG/hcGAS	This study	N/A
pcDNA/HA/hSTING	This study	N/A
pCMV‐3tag‐KSHV‐ORF52	Gift from Fanxiu Zhu (Florida State University, USA) (Wu *et al*, 2015)	N/A
pCMV‐VSV‐G	Gift from Bob Weinberg	Addgene_8454
pDONR/ORF22A	Gift from Jurgen Haas (University of Edinburgh, UK) (Uetz *et al*, 2006)	N/A
pDONR/ORF22B	Gift from Jurgen Haas (University of Edinburgh, UK) (Uetz *et al*, 2006)	N/A
pET‐6xHis‐SUMO2/hcGAS	Generated in previous study (Zhou *et al*, 2019)	N/A
pET‐6xHis‐SUMO2/ORF9	This study	N/A
pET‐6xHis‐SUMO2/ORF9‐N	This study	N/A
pET28a/FLAG‐GFP	This study	N/A
pET28a/FLAG‐ORF9	This study	N/A
pET28a/FLAG‐ORF9‐DM	This study	N/A
pGag‐eGFP	NIH AIDS reagent programme	#11468
pGEX6P1	Gift from Martin Reijns (University of Edinburgh, UK)	GE Healthcare
pGEX6P1/FLAG‐GFP	This study	N/A
pGEX6P1/FLAG‐ORF9	This study	N/A
pGEX6P1/hcGAS	Gift from Martin Reijns (University of Edinburgh, UK)	N/A
pGEX6P1/hcGAS‐HA	This study	N/A
pLenti/FLAG/GFP	This study	N/A
pLenti/FLAG/ORF9	This study	N/A
pLenti/FLAG/ORF9‐DM	This study	N/A
pLenti/FLAG/ORF9‐truncation‐I	This study	N/A
pLenti/FLAG/ORF9‐truncation‐II	This study	N/A
pLenti/FLAG/ORF9‐truncation‐III	This study	N/A
pLenti/FLAG/ORF9‐truncation‐IV	This study	N/A
pLenti/FLAG/ORF9‐truncation‐V	This study	N/A
pLenti/FLAG/ORF9‐truncation‐VI	This study	N/A
pLenti/FLAG/ORF9‐truncation‐VII	This study	N/A
pLenti/FLAG/ORF9‐truncation‐VIII	This study	N/A
pLenti6.3/V5/EMCV‐L	Generated in previous study (Hertzog *et al*, 2018)	N/A
pLenti6.3/V5/hcGAS	This study	N/A
pLenti6.3/V5/hSTING	This study	N/A
pLenti6.3/V5/VZV‐ORF	This study. Expression library for all VZV ORFs (see Figure EV2)	N/A
pRL‐TK	Promega	N/A
pX458/Ruby/hcGAS	This study	N/A
pX458/Ruby/hIRF3	This study	N/A
pX458/Ruby/hMAVS	Generated in previous study (Hertzog *et al*, 2018)	N/A
**Antibodies**		
Donkey‐anti‐mouse‐HRP (WB, 1:2,000)	GE Healthcare	Cat. #NA931, RRID:AB_772210
Donkey‐anti‐rabbit‐HRP (WB, 1:2,000)	GE Healthcare	Cat. #NA934, RRID:AB_772206
Goat‐anti‐rabbit‐AlexaFluor647 (IF, 1:500)	Invitrogen	Cat. #A21246, RRID:AB_1500778
Mouse monoclonal beta‐actin‐HRP (WB, 1:10,000)	Sigma Aldrich	clone AC‐15, RRID:AB_262011
Mouse monoclonal FLAG (IP, 5 μg/sample)	Sigma Aldrich	clone M2, RRID:AB_262044
Mouse monoclonal FLAG‐HRP (WB, 1:10,000)	Sigma Aldrich	clone M2, RRID:AB_439702
Mouse monoclonal HA (IP, 5 μg/sample)	Invitrogen	clone 2‐2.2.14, RRID:AB_10978021
Mouse monoclonal RIG‐I (WB, 1:2,000)	Calteg Medsystems	clone Alme‐1, RRID:AB_2490189
Mouse monoclonal V5 (IP, 5 μg/sample)	Biolegend	Cat. #680602, RRID:AB_2566387
Mouse monoclonal V5‐FITC (IF, 1:400)	Invitrogen	Cat. #R963‐25, RRID:AB_2556567
Mouse monoclonal V5‐HRP (WB, 1:5,000)	Invitrogen	Cat. #R961‐25, RRID:AB_2556565
Mouse monoclonal VZV gE/GI (WB, 1:1,000)	GE Healthcare	Cat. #MAB8612, RRID:AB_2158042
Mouse monoclonal VZV gE/GI‐FITC (Flow cytometry, 1:500)	GE Healthcare, coupled in‐house to FITC	Cat. #MAB8612, RRID:AB_2158042
Mouse monoclonal VZV ORF62 (WB, 1:1,000)	Meridian Life Science	Cat. #C05107MA, RRID:AB_1772162
Rabbit monoclonal FLAG (IF, 1:800)	CellSignaling Technology	clone D6W5B, RRID:AB_2572291
Rabbit monoclonal hcGAS (WB, 1:1,000)	CellSignaling Technology	clone D1D3G, RRID:AB_2799712
Rabbit monoclonal hSTING (WB, 1:1,000)	CellSignaling Technology	clone D2P2F, RRID:AB_2732796
Rabbit monoclonal IRF3 (WB, 1:1,000)	CellSignaling Technology	clone D6I4C, RRID:AB_2722521
Rabbit monoclonal MyD88 (WB, 1:1,000)	CellSignaling Technology	clone D80F5, RRID:AB_10547882
Rabbit monoclonal pSTAT1 (Y701) (WB, 1:1,000)	CellSignaling Technology	clone 58D6, RRID:AB_561284
Rabbit monoclonal pSTAT2 (WB, 1:1,000)	CellSignaling Technology	clone D3P2P, RRID:AB_2800123
Rabbit monoclonal STAT1 (WB, 1:1,000)	CellSignaling Technology	clone 42H3, RRID:AB_2197984
Rabbit monoclonal STAT2 (WB, 1:1,000)	CellSignaling Technology	clone D9J7L, RRID:AB_2799824
Rabbit monoclonal TBK1 (WB, 1:1,000)	CellSignaling Technology	clone D1B4, RRID:AB_2255663
Rabbit polyclonal MAVS (WB, 1:1,000)	ENZO Life Science	Cat. #ALX‐210‐929‐C100, RRID:AB_2050916
Rabbit polyclonal VZV ORF9 (WB, 1:2,000)	Gift from Catherine Sadzot (University of Liege, Belgium) (Riva *et al*, 2013)	N/A
**Oligonucleotides and sequence‐based reagents**		
VZV ORF library cloning PCR primers	This study	Appendix Table S1
Further cloning PCR primer	This study	Appendix Table S2
RT‐qPCR primers	This study	Appendix Table S3
VACV70mer F 5'‐ccatcagaaagaggtttaatatttttgtgagaccatcgaagagagaaagagataaaacttttttacgact‐3'	This study. Sequence described previously (Unterholzner *et al*, 2010)	N/A
VACV70mer R 5'‐agtcgtaaaaaagttttatctctttctctcttcgatggtctcacaaaaa‐tattaaacctctttctgatgg‐3'	This study. Sequence described previously (Unterholzner *et al*, 2010)	N/A
100 bp ISD F 5'‐ACATCTAGTACATGTCTAGTCAGTATCTAGTGATTATCTAGACATACATCTAGTACATGTCTAGTCAGTATCTAGTGATTATCTAGACATGGACTCATCC‐3'	This study. Sequence described previously (Zhou *et al*, 2021)	N/A
100 bp ISD R 5'‐GGATGAGTCCATGTCTAGATAATCACTAGATACTGACTAGACATGTACTAGATGTATGTCTAGATAATCACTAGATACTGACTAGACATGTACTAGATGT‐3'	This study. Sequence described previously (Zhou *et al*, 2021)	N/A
**Chemicals, enzymes and other reagents**		
AlexaFluor‐488 (AF488) carboxylic acid (succinimidyl ester)	Thermo Fisher	Cat. #A20000
Antibody FITC Labeling Kit	abcam	Cat. #ab288100
BL21‐pLysS‐Rosetta	Novagene	Cat. #70954‐3
BL21‐RIL DE3 *E. coli*	Agilent	Cat. #230240
Blasticidin	gibco	Cat. #A1113903
CellTrace Yellow	Invitrogen	Cat. #C34567
DAPI	Invitrogen	Cat. #D1306
diABZi	Cambridge Bioscience	Cat #28054‐500 ug‐CAY
Dual Luciferase assay system	Promega	Cat. #E1910
Dynabeads Protein G	Invitrogen	Cat. #10004D
*E. coli* DNA	Invivogen	Cat. #tlrl‐ecdna
EZBlue Gel Staining Reagent	Sigma Aldrich	Cat. #G1041‐500ML
Gateway LR Clonase II Enzyme mix	Invitrogen	Cat. #11791020
Lipofectamine 2000	Invitrogen	Cat. #11668019
Lipofectamine LTX	Invitrogen	Cat. #15338100
LIVE/DEAD Fixable Violet Dead Cell Stain	Invitrogen	Cat. #L34955
Normal goat serum	Invitrogen	Cat. #10000C
oligo‐dT primers	Invitrogen	Cat. #18418012
PMA	Sigma Aldrich	Cat. #P1585‐1MG
Polybrene	Sigma Aldrich	Cat. #TR‐1003‐G
PreScission Protease	GE Healthcare	Cat. #GE27‐0843‐01
Protease Inhibitor Cocktail	CellSignaling Technology	Cat. #5871
Puyomycin	gibco	Cat. #A1113803
QuantiLuc substrate	Invivogen	Cat. #rep‐qlc1
SuperScript IV Reverse Transcriptase	Invitrogen	Cat. #18090050
SYBR GreenER master mix	Invitrogen	Cat. #11762100
TaqMan Universal PCR Master Mix	Invitrogen	Cat. #4304437
Trimethoprim	Sigma Aldrich	Cat. #46984‐250MG
α32P‐ATP	Perkin Elmer	Cat. #NEG003X250UC
**Software**		
Web‐based iBright Image Analysis software	Thermo Fisher	
FlowJo	FlowJo, LLC	
SnapGene	GSL Biotech	
ApE	M. Wayne Davis, The University of Utah	
Fiji 2.0.0	Schindelin *et al* (2012)	

### Methods and Protocols

#### Cells

All cells were cultured at 37°C and 5% CO_2_ and checked regularly for mycoplasma contamination. Adherent cells were passaged using Trypsin‐EDTA (0.25%) dissociation reagent (Gibco) at appropriate confluence. FCS was obtained from Sigma‐Aldrich. HEK293 and HEK293T cells (gifts from Caetano Reis e Sousa, The Francis Crick Institute, UK) were maintained in DMEM (Sigma‐Aldrich) containing 4.5 g/l glucose, supplemented with 10% (v/v) FCS and 2 mM l‐glutamine (Gibco) (DMEM complete). HEK293‐ISRE‐Firefly luciferase reporter cells (clone 3C11) were described previously (Bridgeman *et al*, 2015). HaCaT cells were a gift from Leonie Unterholzner (Lancaster University, UK) and were maintained in DMEM complete medium. MeWo cells were a gift from Graham Ogg (University of Oxford, UK) and were maintained in MEM supplemented with 10% (v/v) FCS, 2 mM l‐glutamine (Gibco), 1× non‐essential amino acids (Gibco), and 1 mM sodium pyruvate (Gibco). THP1 Dual cells (WT, MyD88‐KO, and IFNAR2‐KO) were from Invivogen. STING‐KO and TBK1‐KO THP1 cells were a gift from Soren Paludan (Aarhus University, Denmark) (Holm *et al*, 2016). MAVS‐KO, cGAS‐KO, and IRF3‐KO cells were generated as described below. All THP1 cell lines were maintained in RPMI (Sigma‐Aldrich) supplemented with 10% (v/v) FCS and 2 mM l‐glutamine (Gibco).

#### Viruses

Varicella‐Zoster virus ROka was a gift from Jeffrey Cohen (NIH, Bethesda, USA; Cohen & Seidel, 1993). The virus was maintained in monolayers of MeWo cells. In brief, monolayers of infected cells were monitored microscopically for cytopathic effect. Cells that showed high level of infection were detached, and infected cells were mixed at appropriate ratios (1:2–1:4) with uninfected cells and re‐plated. Aliquots of infected cells were cryopreserved in freezing medium (90% FCS, 10% DMSO), stored in liquid nitrogen, and thawed for experiments. VZV^ORF9‐V5^ is a recombinant virus, in which a C‐terminal V5 tag was added to the coding sequence of ORF9. The virus was a kind gift from Catherine Sadzot (University of Liege, Belgium; Riva *et al*, 2013). VZV‐ORF9^ecDHFR^ is a recombinant virus, in which an *E*. *coli* dihydrofolate reductase degron motif was fused C‐terminally to the coding sequence of ORF9 (University of Pittsburgh, USA; Warner *et al*, 2021).

#### Plasmids

The p125‐Luc plasmid (*hIFNB1* promoter firefly luciferase) was a gift from T. Fujita (Kyoto University, Japan; Yoneyama *et al*, 2004). pRL‐TK was from Promega. RIG‐I‐CARD was a gift from Andreas Pichlmair (Technical University Munich, Germany). pCMV‐VSV‐G was a gift from Bob Weinberg (Addgene number 8454). The lentiviral packaging plasmid p8.91 was a gift from Greg Towers (University College London). pGag‐eGFP was from the NIH AIDS reagent programme (number 11468). pX458/Ruby/hMAVS was described previously (Hertzog *et al*, 2018). pX458/Ruby/hIRF3 and pX458/Ruby/hcGAS were created as described for the MAVS‐targeting plasmid. sgRNAs targeting exon 3 of the IRF3 locus and exon 2 of the cGAS locus were designed using the MIT algorithm (crispr.mit.edu) and cloned into the pX458 vector. pCMV‐3tag‐KSHV‐ORF52 was a kind gift from Fanxiu Zhu (Florida State University, USA; Wu *et al*, 2015). The pLenti6.3/EMCV‐L‐V5 plasmid was described previously (Hertzog *et al*, 2018).

#### Cloning

Mammalian expression plasmids for hcGAS and hSTING were created by PCR amplification using THP1 cDNA and ligation into the gateway entry vector pCR8. Coding sequences were shuttled into expression vectors (pLenti6.3/C‐V5, pcDNA3.2/C‐FLAG, pcDNA3.2/C‐HA) via Gateway recombination. Mammalian expression plasmids for eGFP (pLenti6.3/puro/N‐FLAG, pDEST31/N‐FLAG) were created in a similar way by PCR amplification from pGag‐eGFP introducing a stop codon. VZV ORF9 expression plasmids (pLenti6.3/puro/N‐FLAG, pDEST31/N‐FLAG) were created by amplification of ORF9 with a stop codon and gateway recombination. Expression plasmids for ORF9 truncation mutants (pLenti6.3/puro/N‐FLAG) were created by PCR amplification of the respective coding sequence with a start and stop codon. ORF9 mutant plasmids were generated using site‐directed mutagenesis (QuikChange II Site‐Directed Mutagenesis Kit, Agilent). GST‐fusion bacterial expression vectors for FLAG‐ORF9, FLAG‐GFP, and hcGAS‐HA were created by PCR amplification of the coding sequences from existing plasmids and restriction enzyme cloning into pGEX6P1. pGEX6P1 and pGEX6P1‐hcGAS were a kind gift from Martin Reijns (University of Edinburgh, UK). Further bacterial expression vectors for FLAG‐ORF9 and FLAG‐ORF9‐DM were created by restriction enzyme cloning of coding sequences into the pET28a bacterial expression vector (kind gift from Simon Davis, University of Oxford). Bacterial expression vectors for ORF9 and ORF9‐N were created by restriction enzyme cloning of coding sequences into a custom 6xHis‐SUMO bacterial expression vector (Zhou *et al*, 2018). All primer sequences are listed in Appendix Table S1.

#### Immunostimulatory dsDNA

*Escherichia coli* dsDNA was from Invivogen. A 70 bp immunostimulatory dsDNA fragment from VACV was described previously (Unterholzner *et al*, 2010). Two complementary oligos were synthesised (Sigma‐Aldrich) and combined at an equal molar ratio. The solution was heated to 95°C and allowed to anneal by cooling to RT.

Forward sequence: 5′‐CCATCAGAAAGAGGTTTAATATTTTTGTGAGACCATCGAAGAGAGAAAGAGA‐TAAAACTTTTTTACGACT‐3′‐TEG‐Biotin.
Reverse sequence: 5′‐AGTCGTAAAAAAGTTTTATCTCTTTCTCTCTTCGATGGTCTCACAAAAA‐TATTAAACCTCTTTCTGATGG‐3′

The 100 bp dsDNA used for the phase separation assay was described previously (Zhou *et al*, 2021). DNA oligos were synthesised (Integrated DNA Technologies) and double‐stranded DNA was prepared by annealing two complementary oligos.

Forward sequence: 5′‐ACATCTAGTACATGTCTAGTCAGTATCTAGTGATTATCTAGACATACATCTA‐GTACATGTCTAGTCAGTATCTAGTGATTATCTAGACATGGACTCATCC‐3′
Reverse sequence: 5′‐GGATGAGTCCATGTCTAGATAATCACTAGATACTGACTAGACATGTACTAGAT‐GTATGTCTAGATAATCACTAGATACTGACTAGACATGTACTAGATGT‐3′

#### Antibodies

##### For immunoblot

beta‐actin‐HRP (AC‐15, Sigma‐Aldrich), FLAG‐HRP (clone M2, Sigma‐Aldrich), V5‐HRP (R961‐25, Invitrogen), RIG‐I (Alme1, Calteg Medsystems), VZV‐gE/GI (MAB8612, GE Healthcare), VZV ORF62 (C05107MA, Meridian Life Science), pSTAT1 (Y701) (58D6, CellSignaling Technology), STAT1 (42H3, CellSignaling Technology), pSTAT2 (D3P2P, CellSignaling Technology), STAT2 (D9J7L, CellSignaling Technology), hcGAS (D1D3G, CellSignaling Technology), hSTING (D2P2F, CellSignaling Technology), MyD88 (D80F5, CellSignaling Technology), TBK1 (D1B4, CellSignaling Technology), MAVS (ALX‐210‐929‐C100, ENZO Life Science), IRF3 (D6I4C, CellSignaling Technology), VZV ORF9 (polyclonal rabbit serum, kind gift from Catherine Sadzot (University of Liege, Belgium) (Riva *et al*, 2013), donkey‐anti‐mouse‐HRP (NA931, GE Healthcare), donkey‐anti‐rabbit‐HRP (NA934, GE Healthcare).

##### For IP

FLAG (clone M2, Sigma‐Aldrich, V5 (680602, Biolegend), HA (2‐2.2.14, Invitrogen).

##### For IF

V5‐FITC (R963‐25, Invitrogen), FLAG (D6W5B, CellSignaling Technology), goat‐anti‐rabbit‐AF647 (A21246, Invitrogen).

##### For FACS

VZV‐gE/gI (see above) was conjugated to FITC using FITC Conjugation Kit (Abcam).

#### VZV ORF Library

Primers for PCR amplification of individual VZV ORF sequences were designed based on an annotated VZV genome sequence (GenBank accession: AB097933.1). Forward primers included a Kozak sequence (GCCGCC), added before the start codon of an ORF. Reverse primers excluded the Stop codon to allow for the addition of a C‐terminal tag through the vector (see below). Primer sequences are listed in Appendix Table S2. The PCR template was generated by extracting RNA from VZV‐infected MeWo cells using QIAshredder (Qiagen) and RNeasy Mini Kit (Qiagen). The RNA was reverse transcribed into cDNA using SuperScript II Reverse Transcriptase (Invitrogen). For some ORFs, DNA from infected MeWo cells extracted with DNeasy Blood and Tissue Kit (Qiagen) served as the PCR template. PCR products were generated using Phusion High‐Fidelity DNA Polymerase (New England Biolabs) or Herculase II Fusion DNA Polymerase (Agilent Technologies). PCR reactions were analysed by agarose gel electrophoresis, and PCR products of the predicted size were extracted from the gel using QIAquick Gel Extraction Kit (Qiagen). Fragments were ligated into the pCR8/TOPO Gateway entry vector (Invitrogen). Plasmid DNA from single clones of transformed *E*. *coli* (New England Biolabs) was analysed for correct orientation of the insert by Sanger sequencing. Inserts were then shuttled into pLenti6.3/TO/V5‐DEST (Invitrogen) using LR Clonase II Enzyme Mix (Invitrogen) for Gateway recombination. All clones with the pLenti6.3/TO/V5 backbone were propagated in recombination‐deficient Stbl3 bacteria (Invitrogen).

Cloning of the entire coding sequence of ORF22 (8,256 bp) was unsuccessful using various amplification and cloning technologies. Therefore, two Gateway entry vectors encoding individual segments within the ORF22 coding sequence (ORF22A: nt 2219‐4029, ORF22B: nt 4,012–6,114, described previously (Uetz *et al*, 2006)) were used (kind gift from Jurgen Haas, University of Edinburgh, UK). Expression vectors with the pLenti6.3/TO/V5 backbone were generated as described above.

To validate expression, HEK293T were seeded at 5 × 10^5^ cells per well in 12‐well plates. The next day, cells were transfected with 600 ng VZV ORF or control expression plasmids using 3 µl Lipofectamine 3000 (Invitrogen) per well. Twenty‐four hours later, cells were lysed in RIPA buffer (10 mM TRIS–HCl pH8, 140 mM NaCl, 1% Triton‐X 100, 0.1% SDS, 0.1% sodium deoxycholate, 1 mM EDTA, 0.5 mM EGTA). Lysates were clarified by centrifugation and 30 µg protein was subjected to immunoblotting.

#### Luciferase reporter assays

HEK293T cells were seeded at 3.5 × 10^4^ cells per well in 96‐well plates. On the following day, cells were transfected with the following plasmids using Lipofectamine 2000 (Invitrogen): 20 ng p125‐F‐Luc, 5 ng pRL‐TK, 1 ng hcGAS, 25 ng hSTING, and 50 ng of a VZV ORF. Alternatively, cells were transfected with 5 ng RIG‐I‐CARD plasmid instead of cGAS and STING plasmids. The next day, expression of firefly and renilla luciferases was assessed using Dual Luciferase assay system (Promega).

Activity of the secreted Lucia luciferase under IRF3 promoter control (THP1 Dual cells) was assessed using QuantiLuc substrate (Invivogen) according to manufacturer’s instructions.

#### Lentivirus production and transduction

1.2 × 10^7^ HEK293T cells were seeded in 15 cm cell culture dishes. The next day, cells were transfected with 9 µg lentiviral expression plasmids harbouring the gene of interest, 9 µg p8.91 plasmid, and 3 µg pVSV‐G using Lipofectamine 2000. Sixteen hours later, the medium was replaced with fresh growth medium. Twenty‐four hours later, lentivirus containing supernatant was harvested, clarified by centrifugation, and stored at 4°C. Cells were overlaid with fresh growth medium. Eight hours later, supernatants were harvested again and pooled with previous supernatants. After 16 h, lentivirus containing supernatants were harvested for the third time. Pooled supernatants from all three harvests were filtered through a 0.45 µm filter, aliquoted into cryovials and stored at −80°C. For transduction of cells, polybrene (Sigma‐Aldrich) was added to lentiviral supernatants to a final concentration of 8 µg/ml. THP1 cells were pelleted and resuspended in lentiviral supernatant containing polybrene. The next day, cells were pelleted and resuspended in fresh growth medium. After overnight incubation, cells were once again pelleted and resuspended in growth medium containing 10 µg/ml blasticidin or 1 µg/ml puromycin (both Gibco) depending on which vector was used. Surviving cells were used for experiments.

#### THP1 knockout cell generation

For generation of knockout cells using CRISPR/Cas9 technology, the pX458‐mRuby plasmid encoding the Cas9 protein, the sgRNA, and mRuby was used (Hertzog *et al*, 2018). The sgRNA sequences were “GAAGTAATATGCACGAGTG” for *cGAS*, “GGCCACCATCTGGATTCCTT” for *MAVS*, and “GGTGGTGCATATGTTCCCGGG” for *IRF3*. 1 × 10^7^ THP1 cells were transiently transfected with 50 µg of respective pX458 plasmids using Lipofectamine LTX (Invitrogen) and incubated overnight. The next day, cells were stained with 10 µg/ml DAPI in PBS for 10 min. After resuspension of cells in growth medium, live, mRuby‐positive cells were FACS‐sorted into Eppendorf tubes containing growth medium with 20% FCS. The suspension of sorted cells was diluted with special growth medium (50% conditioned THP1 medium, 40% fresh RPMI with 10% FCS, 10% additional FCS) to one or three cells per 200 µl. Two hundred microlitre cell suspension were then dispensed into each well of a 96‐well plate (one or three cells per well). The cells were incubated for several weeks until clones grew out.

The absence of the targeted protein in each clone was assessed by immunoblot analysis. For functional validation of MAVS and IRF3 knockout cells, 1.5 × 10^5^ cells were seeded per well into 96‐well plates in growth medium containing 10 ng/ml PMA (Sigma‐Aldrich). The next day, cells were transfected with 5 ng IVT‐RNA (Rehwinkel *et al*, 2010) or 15 ng *E*. *coli* DNA (Invivogen) using Lipofectamine 2000 (Invitrogen). Twenty‐four hours later, IFN in supernatants was measured using the type I IFN bioassay. For functional validation of cGAS knockout cells, cells were stimulated in the same way and activity of Lucia luciferase (under IRF3 promoter control) was assessed in supernatants. Knockout clones were further analysed for insertions and deletions in their genomic loci. *MAVS* and *IRF3* target regions were PCR‐amplified using Herculase II Fusion DNA Polymerase. PCR amplicons were gel extracted (QIAquick Gel Extraction Kit) and sequenced by Sanger sequencing. Sequencing traces were analysed using the TIDE software (https://tide.nki.nl; Brinkman *et al*, 2014).

#### VZV flow cytometry infection assay

Cells were washed in FACS tubes with PBS and incubated with LIVE/DEAD Fixable Violet Dead Cell Stain (Invitrogen) diluted 1:1,000 in PBS for 30 min at 4°C. Cells were washed in PBS and resuspended in 100 µl FACS buffer (1% FCS, 2 mM EDTA in PBS) containing 1:500 FITC‐coupled antibody against VZV‐gE/gI complex. Cells were incubated 30 min at 4°C. After washing with PBS, cells were resuspended in PBS and an equal volume of 8% methanol‐free formaldehyde in PBS was added. Cells were fixed for 15 min at room temperature. Cells were washed with PBS, resuspended in FACS buffer, and analysed on a Attune NxT Acoustic Focusing Cytometer (Thermo Fisher).

#### THP1 VZV co‐culture infections

6.125 × 10^6^ THP1 cells (WT, KOs, or stably transduced with ORF9/GFP) were seeded per well in six‐well plates in growth medium containing 10 ng/ml PMA (Sigma‐Aldrich). As a control, 1.625 × 10^6^ MeWo cells were seeded in MEM. In some experiments, cells were labelled on the next day with a tracer dye (CellTrace Yellow, Invitrogen) before infection. Aliquots of uninfected and VZV‐infected MeWo cells were thawed, washed, and resuspended in MEM. Cells were counted and their concentration was adjusted to 6.25 × 10^5^ live cells per ml (for THP1 cells) or 1.25 × 10^5^ live cells per ml (for MeWo cells) with MEM. Medium was removed from labelled cells, and cells were overlaid with 2 ml MeWo ±VZV cell suspension. Cells were incubated at 37°C for 1 h. The cell suspension was removed, adherent cells were washed with PBS, overlaid with their respective growth medium, and incubated for 48 h. Supernatants were removed from cells, clarified by centrifugation, and stored at −80°C. CXCL10 levels were determined using Human CXCL10/IP‐10 Quantikine ELISA Kit (R&D Systems) according to manufacturer’s instructions. Cells were washed with PBS and incubated with Trypsin‐EDTA until they started to detach. Growth medium was added, cells were resuspended by gentle pipetting, and transferred to tubes. Forty percent of the cell suspension were transferred to Eppendorf tubes on ice for extraction of RNA and generation of protein lysates, respectively. The cells in those Eppendorf tubes were pelleted, washed with PBS, and then lysed either in RLT buffer and processed for RT–qPCR or lysed in RIPA buffer and processed for immunoblot analysis. The remaining 20% of cells were processed for flow cytometry analysis.

#### THP1 transwell VZV infections

PET membrane 1 µm transwell inserts (Sarstedt 83.3930.101) were placed with the bottom membrane facing upwards into 15 cm dishes. Aliquots of uninfected and VZV‐infected MeWo cells were thawed, washed, and resuspended in MEM. Cells were counted and their concentration was adjusted to 3 × 10^6^ live cells per ml with MEM. Five hundred microlitre cell suspension (1.5 × 10^6^ cells) was pipetted onto the transwell membrane, and cells were let to adhere in the incubator overnight. Six‐well plates were filled with RPMI media and prepared transwells were placed cell‐side downwards into the plates, so that the MeWo cells were submerged. 2 × 10^6^ THP1 cells were seeded onto the upward facing side of the transwell membrane in RPMI medium containing 10 ng/ml PMA. Cells were harvested by trypsinisation after 24 h and 48 h and used for downstream analysis. A publication describing this method in more detail is in preparation.

#### VZV‐ORF9^ecDHFR^ infections

THP1 cells were seeded in six‐well plates in growth medium containing 10 ng/ml PMA. The next day, cells were co‐cultured with uninfected or VZV‐ORF9^ecDHFR^‐infected MeWo cells for 1 h in MEM containing 25 µM TMP (trimethoprim, Sigma‐Aldrich). Cells were washed and incubated overnight in THP1 growth medium containing 25 µM TMP. Cells were washed extensively with PBS and then cultured for another 24 h in THP1 growth medium containing 25 µM TMP or not. Cells were harvested and subjected to RNA extraction and RT–qPCR for ISGs and immunoblotting.

#### diABZi stimulations

THP1 cells stably transduced with either ORF9 or GFP were seeded in 12‐well plates in growth medium and were treated with the STING agonist diABZi (Cambridge Bioscience, 28054–500 μg‐CAY) at the concentrations indicated in the figure legends. After 24 h, cells were harvested and expression of IFNs and ISG was assessed by RT–qPCR.

#### Pulldowns

1.4 × 10^7^ HEK293T cells were seeded in 15 cm cell culture dishes. The next day, cells were transfected with 12.7 µg of ORF9‐V5, cGAS‐FLAG, and STING‐HA expression plasmids using Lipofectamine 2000. Sixteen hours later, cells were lysed in IP buffer (20 mM TRIS–HCl pH7.4, 100 mM NaCl, 1 mM EDTA, 0.5% NP‐40, Protease Inhibitor Cocktail; CellSignaling Technology). After clarification, an aliquot was removed as input sample and the lysate was split into four equal volumes. Each aliquot was incubated with 50 µl Dynabeads Protein G (Invitrogen) that were coated with 5 µg α‐V5, α‐FLAG, α‐HA, or control IgG antibody for 1 h under rotation at 4°C. The supernatant was removed, an aliquot was stored as unbound fraction from each sample, and beads were washed three times in lysis buffer. Input, unbound, and bound fractions were subjected to immunoblotting. For pulldowns from stably transduced THP1 cells, cells were seeded at 2 × 10^7^ per dish in 15 cm cell culture dishes in growth medium containing 10 ng/ml PMA. Lysates were generated the next day and processed as described above. For pulldowns of recombinant proteins, 3 µl recombinant cGAS‐HA protein was mixed with 1.5 µl FLAG‐ORF9 or FLAG‐GFP in IP buffer. IP was performed as described above. For pulldowns from VZV‐infected cells, 2.2 × 10^7^ THP1 cells and 4 × 10^6^ HaCaT cells were seeded in 10 cm dishes (in the presence of 10 ng/ml PMA for THP1 cells). The next day, cells were overlaid with 1.25 × 10^7^ MeWo cells infected with VZV or VZV^ORF9‐V5^ for 1 h and afterwards washed with PBS. Lysates were generated 48 h later as described above.

#### Far western

The experimental procedure for far western was based on a protocol previously described (Wu *et al*, 2007). 1.5 µl FLAG‐ORF9 protein or 1.5 µl FLAG‐GFP protein was subjected to SDS–PAGE and blotting as described for regular immunoblotting (see below). After transfer, the membrane was incubated for 30 min at RT temperature in AC buffer (6 M Guanidine HCl, 100 mM NaCl, 20 mM TRIS–HCl pH 7.6, 0.5 mM EDTA, 10% glycerol, 0.1% Tween‐20, 2% skim milk powder, 1 mM DTT). The membrane was then incubated in AC buffers with decreasing concentrations of Guanidine HCl (3 M, 1 M, 0.1 M) for 30 min each. For the last incubation, the membrane was transferred to 4°C and thereafter incubated in AC buffer free of Guanidine HCl overnight. The membrane was blocked for 1 h in 5% milk powder in PBST (PBS with 0.05% Tween‐20) and then incubated with 10 µl recombinant cGAS per 5 ml buffer as probe in 3% milk powder in PBST for 1 h. Membranes were washed for 10 min with PBST thrice and then incubated with α‐cGAS antibody for 1 h. After washing, membranes were incubated with appropriate secondary antibodies, washed again, and imaged. Thereafter, membranes were stripped and re‐probed with α‐FLAG antibody as described for conventional immunoblot.

#### Immunofluorescence

1.75 × 10^5^ HEK293T cells were seeded onto‐glass coverslips. The next day, cells were transfected with 250 ng of cGAS‐V5 or FLAG‐ORF9 expression plasmid using 1.5 µl Lipofectamine 3000 per well. Additional coverslips were co‐transfected with both plasmids. Twenty‐four hours later, cells were washed with PBS and fixed with 4% formaldehyde in cytoskeleton stabilisation buffer (CSB; 10 mM KCl, 274 mM NaCl, 8 mM NaHCO_3_, 0.8 mM KH_2_PO_4_, 2.2 mM Na_2_HPO_4_, 4 mM MgCl_2_, 10 mM PIPES, 4 mM EGTA, 11 mM glucose) for 15 min. Cells were washed and permeabilised with 0.1% Triton X‐100 in CSB for 20 min and incubated with 100 mM glycine in CSB for 10 min afterwards. Cells were washed with PBS four times and blocked using 1% BSA and 5% normal goat serum (Invitrogen) in PBS for 1 h. Coverslips were incubated with primary antibodies in blocking solution for 3 h at room temperature. Cells were washed three times with PBS and incubated with secondary antibodies in blocking solution for 1 h. Coverslips were washed with PBS three times and mounted onto glass slides using ProLong Diamond Antifade Mountant with DAPI (Invitrogen). Images were acquired using a Zeiss LSM780 confocal microscopy system (Zeiss).

#### Recombinant protein expression

##### Single‐step purification of GST‐fusion proteins (see Appendix Fig S4A)

BL21‐pLysS‐Rosetta *E*. *coli* (Novagene) were transformed with bacterial expression vectors. A single colony or a glycerol stock from a single colony were used to inoculate a starter culture of 25 ml LB‐medium containing 34 µg/ml Chloramphenicol and 100 µg/ml Carbenicillin. A large 400 ml LB culture was inoculated using the starter culture. OD600 was measured in intervals and, for VZV ORF9 and GFP, recombinant protein expression was induced by adding 0.1 mM IPTG once OD reached 0.6. Bacteria were grown 3 h at 37°C. For expression of recombinant cGAS and cGAS‐HA, bacteria were grown to OD 0.8 and chilled to 18°C. Protein expression was induced by adding 0.4 mM IPTG, and bacteria were incubated for 16 h at 18°C. For all proteins, bacteria were pelleted at 6,000 g for 7 min and the pellet was resuspended in 12 ml lysis buffer (20 mM TRIS pH7.4, 500 mM NaCl, 0.5 mM EDTA, 0.5 mM EGTA, 0.5% NP40, 1:100 Protease Inhibitor Cocktail; CellSignaling Technology). The suspension was sonicated on ice three times at 20% amplitude with 15 s ON and 30 s OFF (Branson Sonifier). Insoluble material was removed by centrifugation for 15 min at 24,000 *g*. In the meantime, 150 µl Glutathione Sepharose beads (GE Healthcare) were prepared according to manufacturer’s instructions. The beads were added to the clarified lysate and incubated under rotation for 4 h at 4°C. The beads were pelleted by centrifugation at 500 g and washed five times with lysis buffer. The beads were washed once in PreScission Cleavage buffer (50 mM TRIS pH 7.5, 150 mM, 1 mM freshly added DTT) and resuspended in 500 µl PreScission Cleavage buffer. After addition of 12 µl PreScission Protease (GE Healthcare), the suspension was incubated under rotation for 3 h at 4°C. The supernatant containing the recombinant protein was separated from beads and stored at −80°C. Aliquots from the various purification steps were analysed by SDS–PAGE and subsequent staining of the gel with EZBlue Gel Staining Reagent (Sigma‐Aldrich). Based on band intensities, the protein concentrations were estimated to be ca. 1 µg/µl for FLAG‐ORF9 and FLAG‐GFP, and ca. 0.5 µg/µl for cGAS and cGAS‐HA.

##### ORF9 purifications for EMSA and cGAS activity assay (Appendix Fig S4B)

BL21‐pLysS‐Rosetta *E*. *coli* were transformed with bacterial expression vectors. Three fresh, single colonies were used to inoculate a starter culture of 150 ml LB‐medium containing 34 µg/ml chloramphenicol and 50 µg/ml kanamycin and grown overnight at 16°C. Two large 1 L LB cultures were then inoculated using the starter culture. Bacteria were grown to OD 0.8 and chilled to 16°C. Protein expression was induced by adding 0.5 mM IPTG and bacteria were incubated for 16 h at 16°C. Bacteria were pelleted at 6,000 g for 15 min and the pellet was resuspended in 120 ml lysis buffer (20 mM HEPES‐KOH pH 8, 400 mM NaCl, 0.5% NP40, 10% glycerol, 30 mM imidazole, 1 mM PMSF, 5 mM beta‐mercaptoethanol). The suspension was sonicated on ice at 70% amplitude with 15 s ON and 15 s OFF for 8 min total sonication time (Branson Sonifier). Insoluble material was removed by centrifugation for 25 min at 25,000 *g*. In the meantime, 5 ml packed Ni‐NTA resin (Qiagen) were equilibrated in lysis buffer. The beads were added to the clarified lysate and incubated under rotation for 30 min at 4°C. The bead suspension was added to a gravity flow column and the flow through was collected. Beads were washed with 40 ml lysis buffer, 120 ml wash buffer (20 mM HEPES‐KOH pH8, 1 M NaCl, 0.5% NP40, 10% glycerol, 30 mM imidazole), and 80 ml lysis buffer. Proteins were eluted twice with 30 ml elution buffer (20 mM TRIS–HCl pH 7.5, 300 mM NaCl, 300 mM imidazole). Two HiTrap Heparin 5 ml columns (GE Healthcare) were installed in tandem on an Äkta chromatography system (GE Healthcare) and equilibrated with 10 column volumes of HIEX buffer A (20 mM TRIS–HCl pH 7.5, 300 mM NaCl). The Ni‐NTA eluate was applied to the columns, which were subsequently washed with 10 column volumes of HIEX buffer A. Bound proteins were eluted into fractions with a linear salt gradient of HIEX buffer A and HIEX buffer B (20 mM TRIS–HCl pH 7.5, 2 M NaCl). Fractions were analysed by SDS–PAGE, and the desired ones were pooled. After concentration (Amicon Ultra‐15 10 kD Spin Filters, EMD Millipore), proteins were applied to a Superdex 75 increase 10/300 GI column and eluted with the final storage buffer (HEPES‐KOH pH 7.5, 250 mM KCl, 1 mM TCEP). Fractions were analysed by SDS–PAGE, and the desired ones were pooled, concentrated, and stored at −80°C.

##### ORF9 purifications for phase separation assay (Appendix Fig S4C)

Recombinant ORF9 was purified using a protocol previously optimised for expression in MDG and M9ZB media (Zhou *et al*, 2019). Briefly, BL21‐RIL DE3 *E*. *coli* (Agilent) were transformed with pET‐6xHis‐SUMO bacterial expression vectors for full‐length ORF9 or C‐terminally truncated ORF9 1–244 (ORF9‐N). A starter culture of 30 ml grown in MDG‐medium was used to inoculate 2 × 1 L M9ZB‐media cultures. Cultures were grown to an OD of ~2.5, chilled to 16°C, and protein expression was induced with 0.5 mM IPTG before incubation at 16°C for ~16 h. Pelleted bacteria were lysed in lysis buffer (20 mM HEPES‐KOH pH 7.5, 400 mM NaCl, 10% glycerol, 30 mM imidazole, 1 mM DTT) by sonication. Insoluble material was removed by centrifugation, and 6xHis‐tagged protein was purified using Ni‐NTA (Qiagen) and gravity chromatography. Proteins were eluted with 30 ml elution buffer (20 mM HEPES‐KOH pH7.5, 400 mM NaCl, 10% glycerol, 300 mM imidazole, 1 mM DTT), dialysed against low salt buffer (150 mM NaCl) overnight in the presence of 250 μg hSENP2 protease for cleave of the 6xHis‐SUMO tag. Cleaved protein was further purified by HiTrap SP ion‐exchange for full‐length ORF9 and a combination of HiTrap Q ion‐exchange and Superdex 75 size‐exclusion chromatography for ORF9 1–244. Final purified fractions were pooled, concentrated, and flash‐frozen in liquid nitrogen for storage at −80°C.

##### cGAS

Recombinant human cGAS protein used for EMSA and cell‐free cGAS activity assays was purified as described previously (Zhou *et al*, 2019).

#### Immunoblotting

Protein concentrations in lysates were determined using Pierce BCA Protein Assay Kit (Thermo Scientific) and equalised by dilution of samples with lysis buffer. Subsequently, 4× NuPAGE LDS Sample Buffer (Invitrogen) was added and samples were incubated at 95°C for 10 min. Samples were run on NuPAGE Novex 4–12% Bis‐TRIS gels (Invitrogen) using NuPage MOPS‐SDS running buffer (Invitrogen). Proteins were subsequently blotted onto PROTRAN Pure nitrocellulose membrane (PerkinElmer) using transfer buffer (25 mM TRIS, 192 mM glycine). Membranes were blocked with 5% skim milk powder (Sigma‐Aldrich) in TBS containing 0.1% Tween‐20 (5% milk TBS‐T) for 1 h at room temperature and were then incubated with primary antibodies in 5% milk TBS‐T overnight at 4°C. Primary antibodies that bind to phosphorylated residues were diluted in 5% BSA in TBS‐T. Membranes were washed thrice with TBS‐T and incubated with HRP‐coupled secondary antibodies in 5% milk TBS‐T for 1 h at room temperature. After three further washes with TBS‐T, proteins were detected using Western Lightning Plus‐ECL (PerkinElmer) and the iBright FL1000 Imaging System (Thermo Fisher) or Amersham Hyperfilm MP (GE Healthcare). If needed, antibodies were stripped from the membrane with stripping buffer (200 mM glycine, 0.1% SDS, 1% Tween‐20, pH 2.2) for 20 min at room temperature. Membranes were washed with TBS‐T, blocked as described above, and re‐probed.

#### RT–qPCR

Cells were lysed in RLT Plus buffer and RNA was extracted using RNeasy Plus Mini Kit (Qiagen). The RNA was reverse transcribed using SuperScript IV Reverse Transcriptase (Invitrogen) and oligo‐dT primers (Invitrogen). The qPCR reaction containing TaqMan Universal PCR Master Mix (Applied Biosystems) and TaqMan Primer/Probes was run on QuantStudio 7 Flex Real‐Time PCR System (Thermo Fisher) with standard settings. Gene expression was analysed with the Ct method using *GAPDH* expression for normalisation. Taqman primer/probes used were: *GAPDH* (Hs02758991_g1), *IFNB1* (Hs02621180_s1), *IFI44* (Hs00951349_m1), *IFIT1* (Hs03027069_s1), and *IFNL1* (Hs00601677_g1). RT–qPCR for VZV transcripts was performed with SYBR GreenER master mix (Invitrogen) using primer pairs indicated in Appendix Table S3.

#### Cell‐free cGAS activity assay

Cell‐free cGAS activity assays were performed according to a previously described procedure (Kranzusch *et al*, 2013). Full‐length recombinant human cGAS protein (1 μM) and VACV70mer DNA (1 μM) were incubated in 20 μl reaction buffer (50 mM TRIS–HCl pH 7.5, 10 mM Mg(OAc)_2_, 10 mM KCl, 1 mM DTT, 25 µM ATP, 25 µM GTP, 1 µCi α32P‐ATP; PerkinElmer) for 90 min at 37°C. Reactions were terminated for 5 min at 95°C. Leftover ATP was converted to inorganic phosphate by addition of 10 U calf‐intestinal phosphatase and incubation for 20 min at 37°C. Two microlitres of each reaction were spotted onto a PEI Cellulose F thin‐layer chromatography plate (EMD Millipore) and run in 1.5 M KH_2_PO_4_. Plates were dried at room temperature and exposed to a phosphorscreen overnight. Screens were imaged using a Typhoon FLA 9500 imager (GE Healthcare). Signal intensities were quantified using Fiji 2.0.0 software (Schindelin *et al*, 2012) and normalised to the average signal in conditions with BSA.

#### Phase separation assays

*In vitro* phase separation was performed as previously described (Du & Chen, 2018; Zhou *et al*, 2021). Briefly, human cGAS was labelled with AlexaFluor‐488 (AF488) carboxylic acid (succinimidyl ester) (Thermo Fisher Scientific) according to manufacturer’s manuals using a molar ratio of 1:10 at 4°C for 4 h. Excess free dye was removed by dialysis against buffer (20 mM HEPES‐KOH pH 7.5, 250 mM KCl, 1 mM DTT) at 4°C overnight, and then human cGAS was further purified on a PD‐10 desalting column (GE Healthcare) eluted with storage buffer (20 mM HEPES‐KOH pH 7.5, 250 mM KCl, 1 mM TCEP) as previously described (Zhou *et al*, 2021).

To induce phase separation, human cGAS, ORF9, or ORF9 truncation (5–15 μM each) was incubated with 100 bp dsDNA (5–10 μM, containing 10% Cy3‐labelled DNA) in the presence of various salt concentrations at 25°C in a total reaction volume of 20 μl. The details of proteins, nucleic acids, and salt concentrations are provided in figure legends. Reactions were placed in 384‐well non‐binding microplates (Greiner Bio‐One) and incubated at 25°C for 30 min prior to imaging to allow condensates to settle. Fluorescence microscopy images were acquired at 25°C using a Leica TCS SP5 X (Leica Microsystems) mounted on an inverted microscope (DMI6000; Leica Microsystems) with an oil immersion 63×/numerical aperture 1.4 objective lens (HCX PL APO; Leica Microsystems). Labelled proteins were detected with excitation at 488 nm (emission at 500–530 nm), and DNA was detected with excitation at 550 nm (emission at 560–590 nm). Microscopy images were processed with Fiji (Schindelin *et al*, 2012) and contrast adjusted with a uniform threshold setup for each enzyme.

#### Protein sequence analysis

The HSV‐1 VP22 (PDB: 4XAL) and MHV68 ORF52 (PDB: 2OA5) crystal structures were used to graphically represent secondary structure features. For VZV ORF9 (UniProt accession Q4JQW6) and KSHV ORF52 (UniProt accession F5HBL8), structural features and sequence disorder was predicted by the PROFphd secondary structure prediction algorithm through submission of protein sequences to the predictprotein.org web interface (Yachdav *et al*, 2014).

#### Data analysis and software

Data were analysed using Excel for Mac (Microsoft) and GraphPad Prism 8 (GraphPad Software). SnapGene (GSL Biotech) and ApE (M. Wayne Davis, The University of Utah) were utilised for DNA sequence analysis to assist cloning. Statistical analysis is detailed in the figure legends and was performed using GraphPad Prism 8. Sample sizes were estimated based on prior experience and the literature. Randomisation and blinding were not performed. Graphs and figures were created using GraphPad Prism 8 and Adobe Illustrator CC (Adobe Systems). Immunoblot images were processed using web‐based iBright Image Analysis software (Thermo Fisher). Flow cytometry data was analysed using FlowJo (FlowJo, LLC). Fiji 2.0.0 software was used to process confocal microscopy images (Schindelin *et al*, 2012).

## Author contributions

**Jonny Hertzog:** Conceptualization; Data curation; Formal analysis; Validation; Investigation; Visualization; Methodology; Writing—original draft; Project administration; Writing—review and editing. **Wen Zhou:** Resources; Investigation; Visualization; Methodology; Writing—review and editing. **Gerissa Fowler:** Investigation; Visualization; Methodology; Writing—review and editing. **Rachel E Rigby:** Conceptualization; Investigation; Methodology; Writing—review and editing. **Anne Bridgeman:** Investigation; Writing—review and editing. **Henry TW Blest:** Investigation; Writing—review and editing. **Chiara Cursi:** Investigation; Methodology; Writing—review and editing. **Lise Chauveau:** Resources; Writing—review and editing. **Tamara Davenne:** Methodology; Writing—review and editing. **Benjamin E Warner:** Resources; Writing—review and editing. **Paul R Kinchington:** Resources; Writing—review and editing. **Philip J Kranzusch:** Supervision; Writing—review and editing. **Jan Rehwinkel:** Conceptualization; Formal analysis; Supervision; Funding acquisition; Validation; Visualization; Writing—original draft; Project administration; Writing—review and editing.

In addition to the CRediT author contributions listed above, the contributions in detail are:

JH, RER, and JR conceived the study. JH, WZ, GF, RER, and JR designed the experiments and analysed the data. JH, WZ, GF, AB, HTWB, CC, LC, and TD performed the experiments. JH, WZ, RER, and PJK developed the methodology. BEW and PRK developed and provided the reagents. JH and JR wrote the manuscript with help from all authors. All authors read and approved the final manuscript.

## Disclosure and competing interests statement

The authors declare that they have no conflict of interest.

## Supporting information



Appendix

Expanded View Figures PDF

Source Data for Figure 1

Source Data for Figure 2

Source Data for Figure 3

Source Data for Figure 4

Source Data for Figure 6

## Data Availability

This study includes no data deposited in external repositories.
