# Supplementary material for: Varicella‐Zoster virus ORF9 is an antagonist of the DNA sensor cGAS
Source: EMBO J. 2022 Jun 7;41(14):e109217. doi: 10.15252/embj.2021109217 (PMC9289529; doi:10.15252/embj.2021109217)
Supplement: Supplementary file 1 — Appendix [file EMBJ-41-e109217-s004.pdf]

APPENDIX

Varicella-Zoster Virus ORF9 Is an Antagonist of the DNA Sensor cGAS

Jonny Hertzog[1], Wen Zhou[2,3,‡], Gerissa Fowler[1], Rachel E. Rigby[1], Anne Bridgeman[1], Henry T. W. Blest[1], Chiara Cursi[1], Lise Chauveau[1], Tamara Davenne[1], Benjamin E. Warner[5], Paul R. Kinchington[5,6], Philip J. Kranzusch[2,3,4], and Jan Rehwinkel[1,*]

[1]MRC Human Immunology Unit, MRC Weatherall Institute of Molecular Medicine, Radcliffe Department of Medicine, University of Oxford, Oxford, UK
[2]Department of Microbiology, Harvard Medical School, Boston, MA, USA.
[3]Department of Cancer Immunology and Virology, Dana-Farber Cancer Institute, Boston, MA, USA.
[4]Parker Institute for Cancer Immunotherapy, Dana-Farber Cancer Institute, Boston, MA, USA.
[5]Department of Ophthalmology, University of Pittsburgh, Pittsburgh, Pennsylvania, USA.
[6]Department of Microbiology and Molecular Genetics, University of Pittsburgh, Pittsburgh, Pennsylvania, USA.
[‡]Present address: School of Life Sciences, Southern University of Science and Technology, Shenzhen, Guangdong 518055, China.

*correspondence: jan.rehwinkel@imm.ox.ac.uk

TABLE OF CONTENTS

Appendix Figures 1-4

Appendix Fig S1: IRF3, MAVS, and cGAS THP1 Dual knockout clone validation. Related to Fig 1.
Appendix Fig S2. Validation of THP1 knockout cells. Related to Fig 1.
Appendix Fig S3: Flow cytometry gating strategy for VZV-infected THP1 cells. Related to Fig 1C.
Appendix Fig S4. SDS-PAGE analysis of recombinant protein production. Related to Fig 3-6.

Appendix Tables 1-3

Appendix Table S1: Primers for Cloning
Appendix Table S2: Primers for VZV ORF PCR
Appendix Table S3: Primers for VZV RT-qPCR using SYBR Green

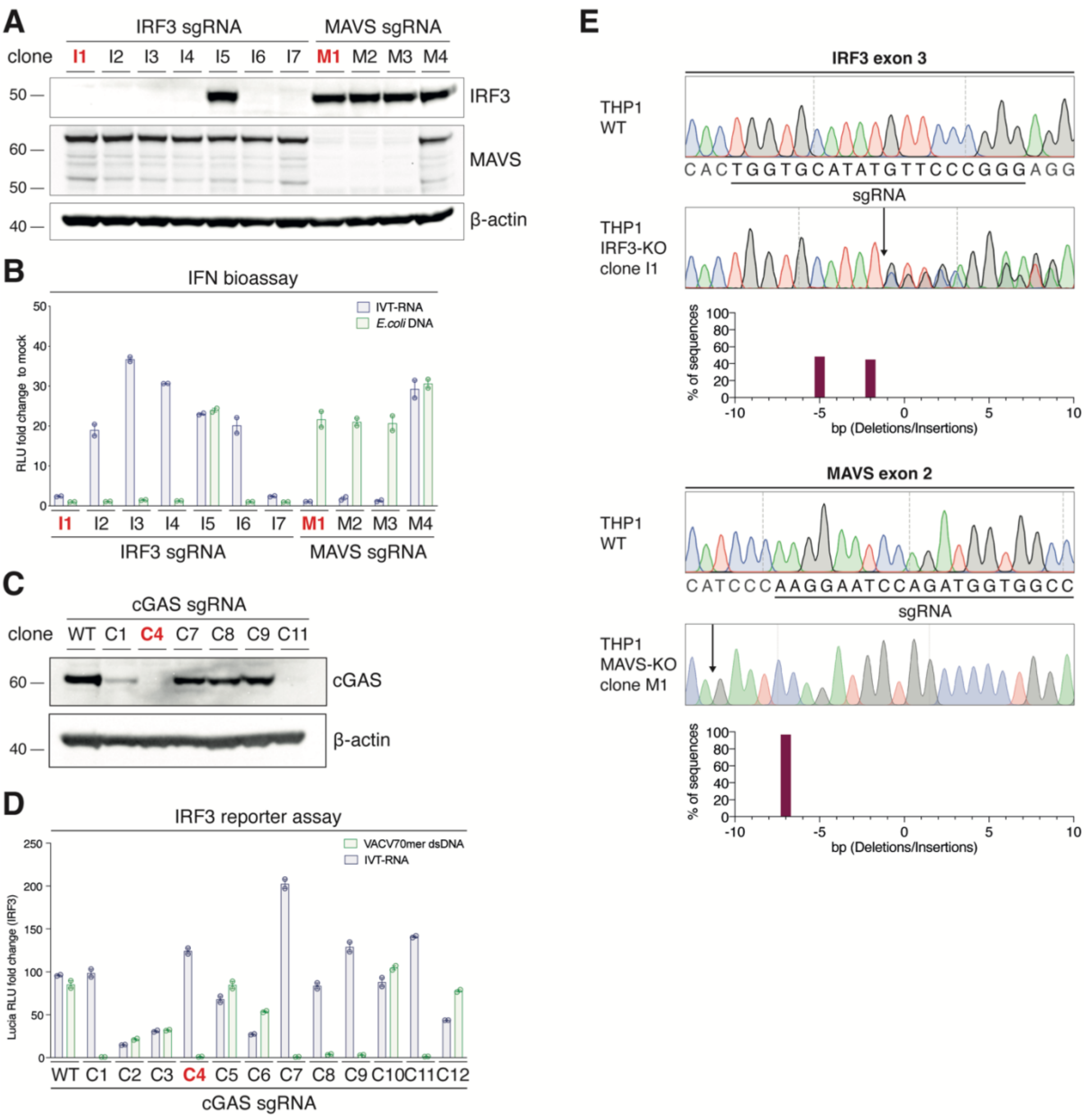

Appendix Fig S1: IRF3, MAVS, and cGAS THP1 Dual knockout clone validation. Related to Fig 1.

(A, C) THP1 Dual cells were transfected with plasmids encoding *IRF3*, *MAVS* or *cGAS* targeting sgRNAs, Cas9, and a fluorescent marker protein. Cells expressing the marker were purified by FACS and subjected to limiting dilution. Clones were expanded and tested by immunoblotting. Membranes were probed with the indicated antibodies. **(B)** Cells from the same clones as in (A) were PMA-differentiated and transfected with IVT-RNA or *E.coli* DNA. The next day, supernatants were analysed by IFN bioassay (see methods). Fold changes were calculated based on supernatants from mock-transfected cells. **(D)** Cells from clones in (C) were PMA-differentiated and transfected with IVT-RNA or VACV70mer dsDNA. The next day, luciferase activity was determined in cell supernatants. Fold changes were calculated relative to mock-transfected cells. **(E)** The *IRF3* or *MAVS* locus was PCR-amplified from genomic DNA and analysed by Sanger sequencing. Panels show electropherograms from WT cells and the knockout clones used for experiments (highlighted in red in A-B). Sequencing traces were analysed using the TIDE algorithm.

Data are from one experiment. In (B) and (D), average and range (technical duplicates) are shown.

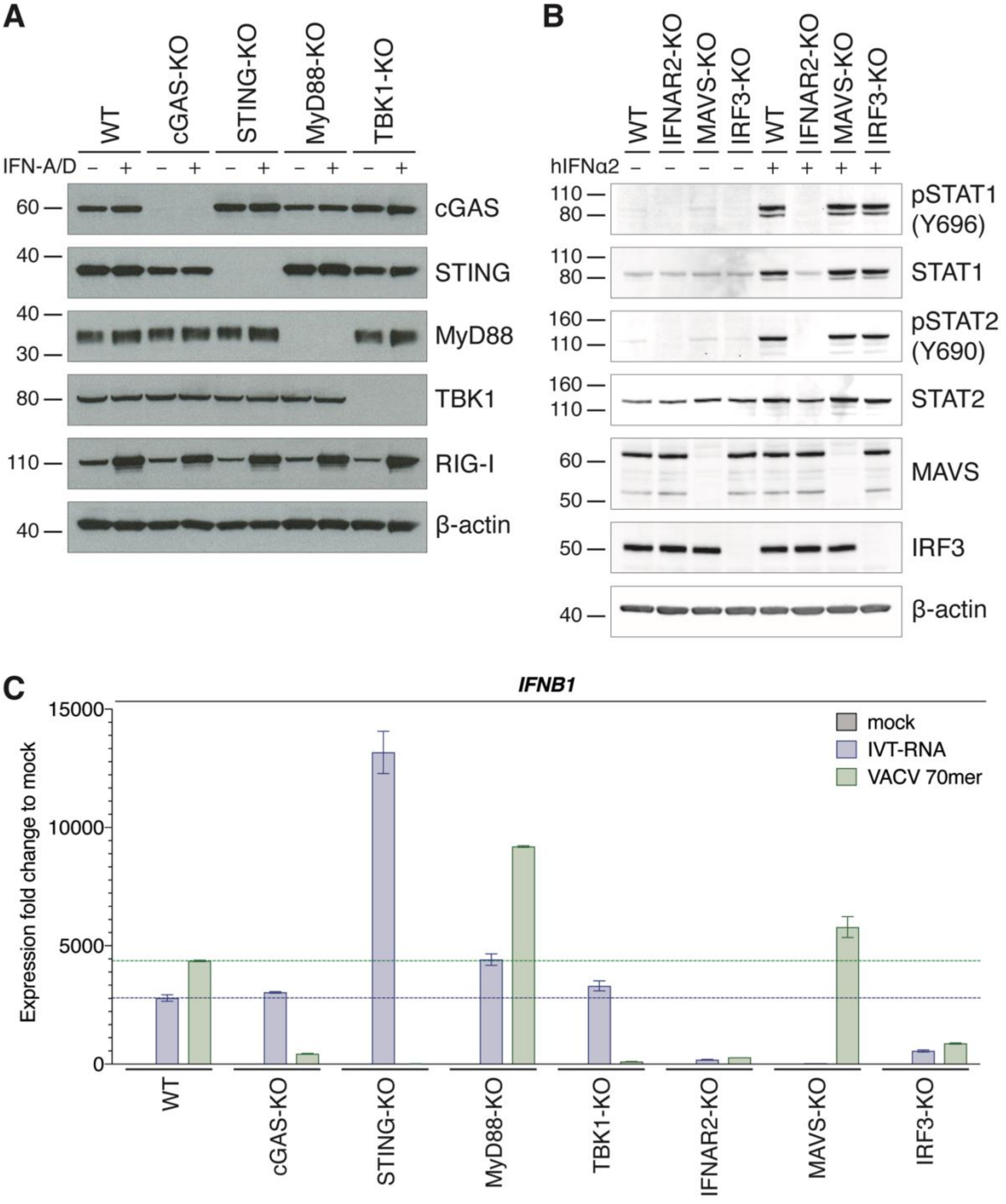

Appendix Fig S2. Validation of THP1 knockout cells. Related to Fig 1.

(A,B) The indicated THP1 cell lines were differentiated with PMA and treated or not with recombinant type I IFN as indicated. Lysates were processed for immunoblotting with the indicated antibodies. **(C)** PMA-differentiated THP1 cells were transfected with 200ng IVT-RNA or 500ng VACV 70mer DNA, or were treated with transfection reagent only (mock). RNA was extracted and RT-qPCR was performed for the *IFNB1* transcript. Horizontal lines indicate expression fold changes in WT cells.

Panel (A) is representative of three independent experiments. Panels (B) and (C) show data from one experiment. In (C), bars indicate the average and error bars the range of technical duplicates.

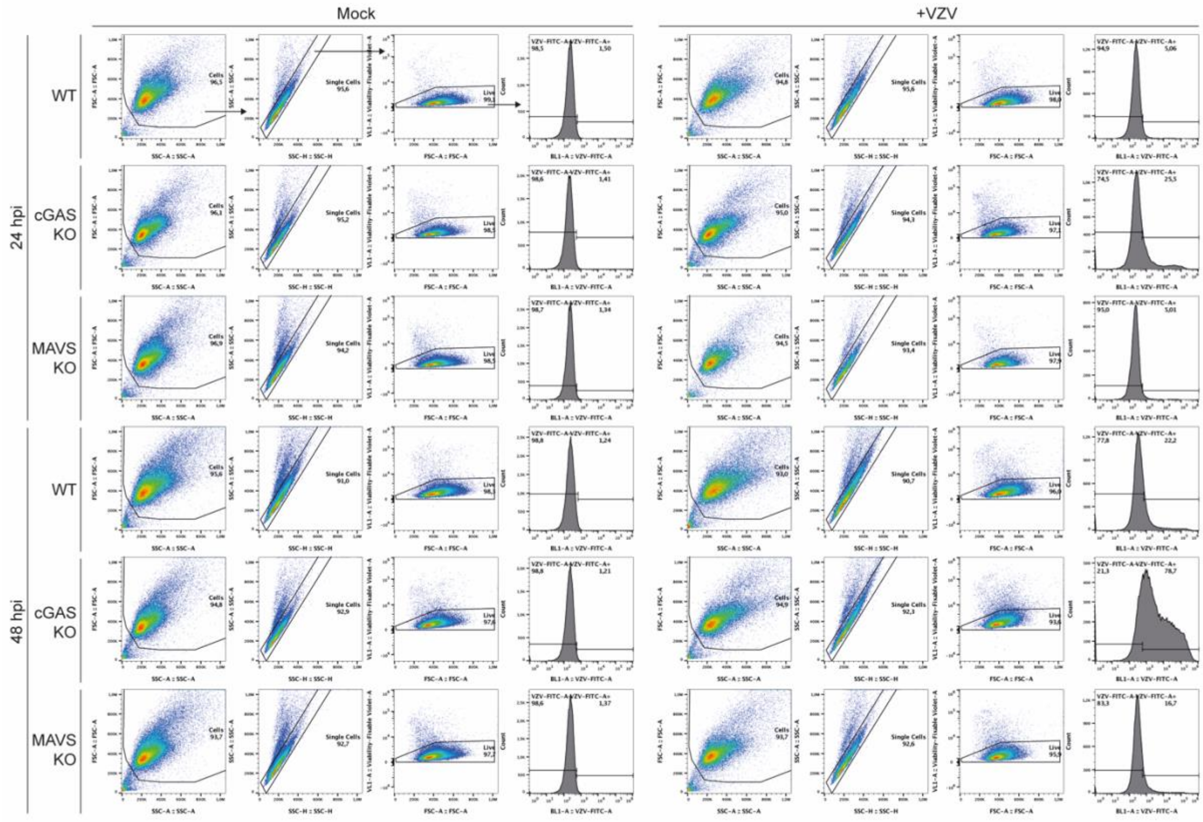

Appendix Fig S3: Flow cytometry gating strategy for VZV-infected THP1 cells. Related to Fig 1C.
Cells were stained with Fixable Violet Viability dye and a FITC-coupled antibody against the VZV-gE/gI heterodimer on the cell surface. FITC fluorescence was quantified after gating on single, live cells as indicated by arrows in the first row. Panel shows representative results of four independent experiments.

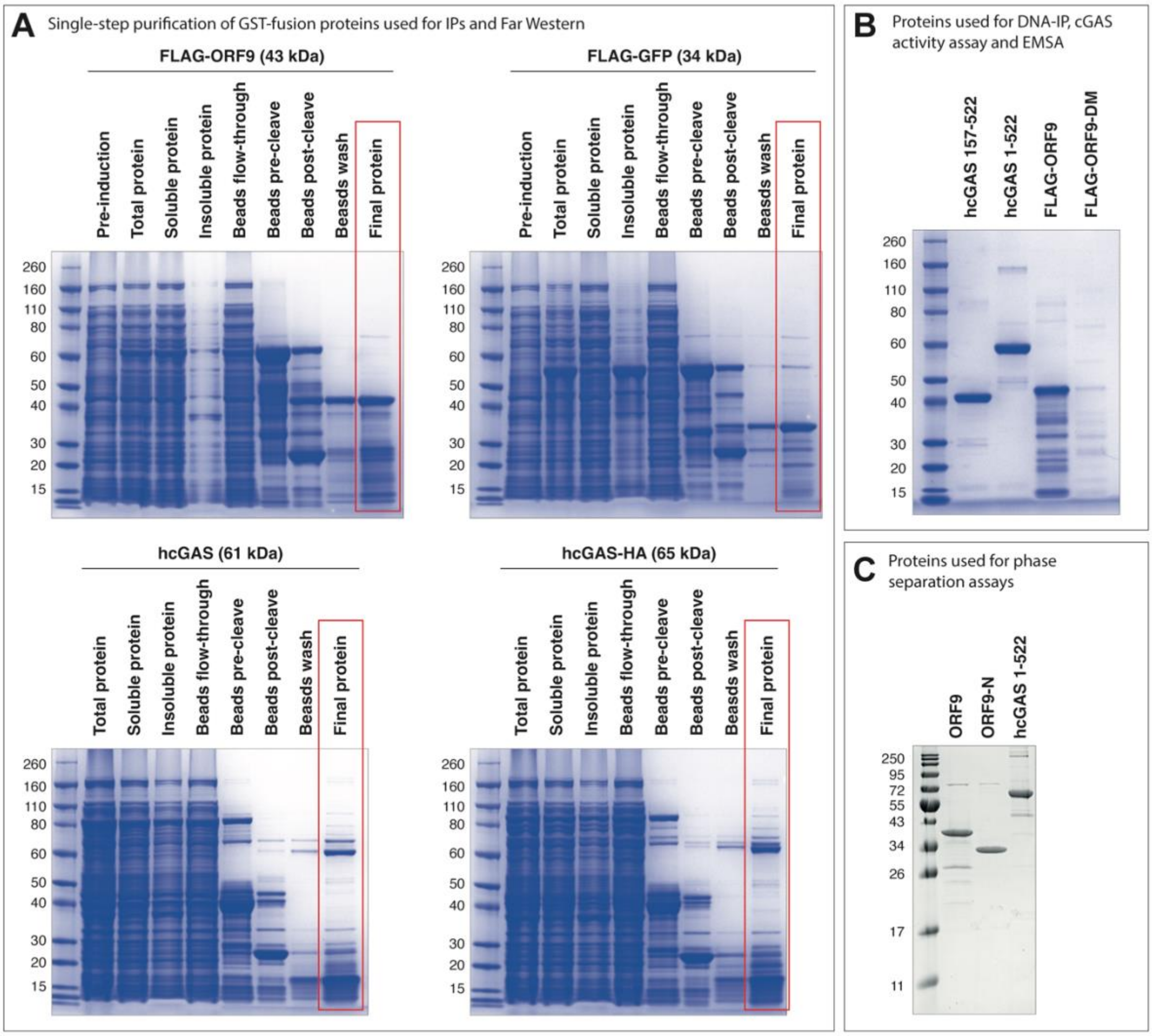

Appendix Fig S4. SDS-PAGE analysis of recombinant protein production. Related to Fig 3-6.
(A) Related to Fig 3F, 3G and 4D. Images of Coomassie stained gels from production of GST-fusion recombinant proteins. See methods for details. **(B)** Related to Fig 4F, 6F and 6G. Images of Coomassie stained gels from recombinant proteins used in DNA-IPs, cGAS activity assays and agarose gel EMSA. **(C)** Related to Fig 5. Images of Coomassie stained gels from recombinant proteins used in phase separation assays. See methods for details.

Appendix Table S1: Primers for Cloning

Name	F primer sequence 5'-3'	R primer sequence 5'-3'
hcGAS	gccgccatgcagccttggcacg	aaattcatcaaaaactggaaactcattg
hSTING	gccgccatgccccactccagc	agagaaatccgtgcggag
eGFP	gccgccatggtgagcaagggcgag	cttgtacagctcgtccatgcc
ORF9-STOP	gccgccatggcatcttccgacggtga	ctattttcgcgcatcagttcttgatg
ORF9-I-S	gccgccatggcatcttccgacggtga	ctaggcaattgcgcctgctcc
ORF9-II-S	gccgccatgagcgggagaccaatttccttcag	ctattttcgcgcatcagttcttgatg
ORF9-III-S	gccgccatgtccagcggatcggaagatg	ctattttcgcgcatcagttcttgatg
ORF9-IV-S	gccgccatggcatcttccgacggtga	ctataggtctgcttcattagcggcttg
ORF9-V-S	gccgccatgtccagcggatcggaagatg	ctataggtctgcttcattagcggcttg
ORF9-VI-S	gccgccatgagcgggagaccaatttccttcag	ctataggtctgcttcattagcggcttg
ORF9 mutant A	gcgaccgcttcacaaaagacggctgcgttatatg atggcgtaggacc	ggtcctacgccatcatataacgcagccgtcttttg tgaagcggtcgc
ORF9 mutant B	ctgcatggcggctacggccgcgaccgcttcacaa aag	cttttgtgaagcggtcgcggccgtagccgccatg cag
FLAG-ORF9-BamHI (pGEX6P1)	gggcccctgggatccgactacaaagaccatg-acggtga	gaattccggggatccctattttcgcgc -atcagttcttg
FLAG-GFP-BamHI (pGEX6P1)	gggcccctgggatccgactacaaagaccatg-acggtga	gaattccggggatccctacttgtacag-ctcgtccatgc
FLAG-ORF9/ORF9-DM (pET28a)	GGCAGCCATATGGACTACAAAGACCA TGACGGTG	GCTGCCGTCGACCTACTTGTACAGCT CGTCCATGC

Appendix Table S2: Primers for VZV ORF PCR

ORF	F primer sequence 5'-3'	R primer sequence 5'-3'
0	GCCGCCatggcgaccgtgcactactcc	tgtagttgagttgggaggttcctcgg
1	GCCGCCatgtccagggtatcggagtatgggg	ttctcgcttgcagcttgtcgc
2	GCCGCCatgcatgtaatttctgagacac	catcaatacgccctccg
3	GCCGCCatggatacaacgggagcttccg	tagtccgccgacagccg
4	GCCGCCatggcctctgcttcaattc	gcagttaaaggtactacacttaaa
5	GCCGCCatgcaggctttaggaatca	atgtttctgggagttttcac
6	GCCGCCatggataaatcctccaaacc	actcgaagttaaatttggataatt
7	GCCGCCatgcagacggtgtgtgcc	tacaagcataacatgggatttcttga
8	GCCGCCatgaacgaagcggtaattg	atgttttagtagaaaatcgacat
9	GCCGCCatggcatcttccgacggtga	ttttcgcgcatcagttcttga
9A	GCCGCCatgggatcaattaccgcttcg	ccacgtgctgcgtaatacagaac
10	GCCGCCatggagtgtaatttaggaaccg	acgcgttaaaaacccaca
11	GCCGCCatgcagtcgggtcattataa	atattttcgtagtaaatgcatgg
12	GCCGCCatgttttctcggtttgcgcg	atgatgactcttaggcgtatttttcct
13	GCCGCCatgggagacttgtcatgttg	aagagccatttccatttttaggg
14	GCCGCCatgaagcggatacaaataaatttaatt	tgaacagcaacggatgca
15	GCCGCCatggccgtgaatggtgaa	cgatacatatgtaccacatagatagc
16	GCCGCCatggatttgaggtcgcgt	tttaactgtacatattacgtcagattcac
17	GCCGCCatggggctctttggactga	attccaatattttgttaatacag
18	GCCGCCatggatcagaaagattgc	taaatcgtttatcactgtgc
19	GCCGCCatggagttcaaaagaattttta	taaagcacaactggtac
20	GCCGCCatggggagtcaaccaacc	ataataacattcgttccatgtatttgt
21	GCCGCCatggaagaaccaatttgtta	agggtcactcccacttg
23	GCCGCCatgacacaacccgcatcg	caccctacgacttcttgaagc
24	GCCGCCatgtcacggagaacgtatg	tttccagaaaagcaccgc
25	GCCGCCatgtacgaatcggaaaatg	agcatccttcaatatttcatg
26	GCCGCCatggatcgggtagaatcag	gacatacttcgatagggtg
27	GCCGCCatgcatttaaagcctaccag	ccgaggaggaacaaagt
28	GCCGCCatggcgatcagaacgggg	actttgatggagaattgcttttggaa
29	GCCGCCatggaaaatactcagaagact	aatcatttccattgtaatgttcc
30	GCCGCCatggaattggatattaatcgaacattg	tgaaaacgccgggtc
31	GCCGCCatgtttgttacggcggtt	cacccccgttacattctcg
32	GCCGCCatggaatcgtctaacattaacg	atcggtgtcagaatcttcat
33	GCCGCCatggctgctgaagctgac	acaccgccccaccatcat
33.5	GCCGCCatggcttctgtagcaggtaacgc	acaccgccccaccatcat
34	GCCGCCatgacggcgagatatgggtt	cggtgtggaggcaaact
35	GCCGCCatgtccgctagtcgaattcgg	cccatgggaaaacatcccgg
36	GCCGCCatgtcaacggataaaaccgat	ggaagtgttgtcctgaacg
37	GCCGCCatgtttgcgctagttttagc	tgtcagaggtattttattatattct

38	GCCGCCatggaatttccatatcattcaac	cctttgggttttttccc
39	GCCGCCatgaacccaccccaagcccg	aaacgaaatagatgtttttaacataacacgg
40	GCCGCCatgacaacggtttcatgt	tcgcggaagaggaaga
41	GCCGCCatggctatgccatttgagat	cacttgaatcacggcc
42	GCCGCCatgtcattgataatgtttggt	tttaataggcataaacacgg
43	GCCGCCatggaagcccatttggca	tttatgggggttgggaatagag
44	GCCGCCatggaattacaacgcatatttccg	ggtggttgtaggttccggt
45	GCCGCCatgtcattgataatgtttggtcgtacg	tttaataggcataaacacggaatccg
46	GCCGCCatgtcaggccacactcca	cacatccgtgtgtggggt
47	GCCGCCatggatgctgacgacacacc	tgtcgatcctatccaatcccg
48	GCCGCCatggcacgatcgggattg	aagcaacggtttctccg
49	GCCGCCatgggacaatcttcatccag	acattttgcgcatttggaa
50	GCCGCCatgggaactcaaaagaaggg	ctcccacccactgtt
51	GCCGCCatgtctcccaacaccggg	taaactttcaaaatttaccgccccg
52	GCCGCCatggacgcaacgcagatt	taaaaacaagaagttatatgaagc
53	GCCGCCatgcagcggattcgacct	ctttacaacccgtggtgaattttt
54	GCCGCCatggccgaaataacgtct	agatcttcgatcacgtcg
55	GCCGCCatgaaaagatcaatttctgt	atacacaacgtgtacg
56	GCCGCCatgaaaaatccgcagaaattagcga	cgcgtttgcggcgtccc
57	GCCGCCatggacgtacgagaacgtaatg	acgttgaggagccttgc
58	GCCGCCatgttttcggagttgcctcc	cgttctcgtacgtccatga
59	GCCGCCatggatgtgtctggggag	tataacactccaatcgatctcg
60	GCCGCCatggcatcacataaatggttactgc	ttggcatacgcgttggaacaaa
61	GCCGCCatggataccatattagcg	ggacttcttcatcttg
62/71	GCCGCCatggatacgccgccgatgc	cccccgactctgcgggg
63/70	GCCGCCatgttttgcacctcaccggcta	cacgccatgggggggc
64/69	GCCGCCatgaatctctgcggatcccg	ggatctctcgtgggttcttgg
65	GCCGCCatggccggacaaaacacc	tccaacaaattgtgacgttat
66	GCCGCCatgaacgacgttgatgcaac	atctccaacttccattggatttg
67	GCCGCCatgtttttaatccaatgtttgat	tttaacaaacgggtttaca
68	GCCGCCatggggacagttaataaacc	ccgggtcttatctatatacaccgtgt

Appendix Table S3: Primers for VZV RT-qPCR using SYBR Green

GAPDH	CATGGCCTTCCGTGTTCCTA	CCTGCTTCACCACCTTCTTGAT
ORF40	CCGACACGCCAGGGAACCTA	CACACCGTCAACCTGCCGTC
ORF54	TCCAACCCCTCTTCGGCTCG	GGGGATGGCCGATGGGATGT
ORF63	CCGACGCGGAATCATCGGAC	TGTTGCACCCATCCCCGTCT
