# Supplementary material for: Varicella‐Zoster virus ORF9 is an antagonist of the DNA sensor cGAS
Source: EMBO J. 2022 Jun 7;41(14):e109217. doi: 10.15252/embj.2021109217 (PMC9289529; doi:10.15252/embj.2021109217)
Supplement: Supplementary file 2 — Expanded View Figures PDF [file EMBJ-41-e109217-s003.pdf]

Expanded View Figures

Figure EV1. The cGAS/STING DNA sensing pathway induces type I IFNs in response to VZV infection. Related to Fig 1.

A Schematic of the THP1-MeWo co-culture VZV infection system. See text for details.
B A panel of THP1 knockout cell lines was mock infected or infected with VZV as shown in (A). The mRNA expression levels of *IFNB1* (encodes IFNβ) and *IFI44* were analysed by RT–qPCR. Expression levels were normalised to *GAPDH* and are shown as fold changes relative to levels in uninfected cells.
C Cells infected as in (A) were analysed by western blot using the indicated antibodies.
D Levels of CXCL10 (IP-10) in co-culture supernatants were quantified by ELISA.
E WT THP1 cells infected as in (A) were analysed by flow cytometry. Left and right panels show gating strategy for (F) and (G), respectively.
F Quantification of inoculum (MeWo) and target cells (THP1) in co-culture experiments.
G Quantification of VZV-infected cells within THP1 target cell population.

Data information: The different shapes of data points in (B) and (D) correspond to independent biological repeat experiments. Panels (B), (D), (F), and (G) show pooled data from six (THP1 WT, MeWo) or three (THP1 KOs) independent biological repeats ($n = 3/6 \pm$ SEM). Panel (C) shows a representative result of three independent repeats. Panel (E) shows a representative result of six independent repeats. Statistical analysis in panel (B) was one-way ANOVA with Dunnett's multiple comparisons test. $^{**}P < 0.01$, $^{*}P < 0.05$, ns = not significant.

 © 2022 The Authors

 © 2022 The Authors

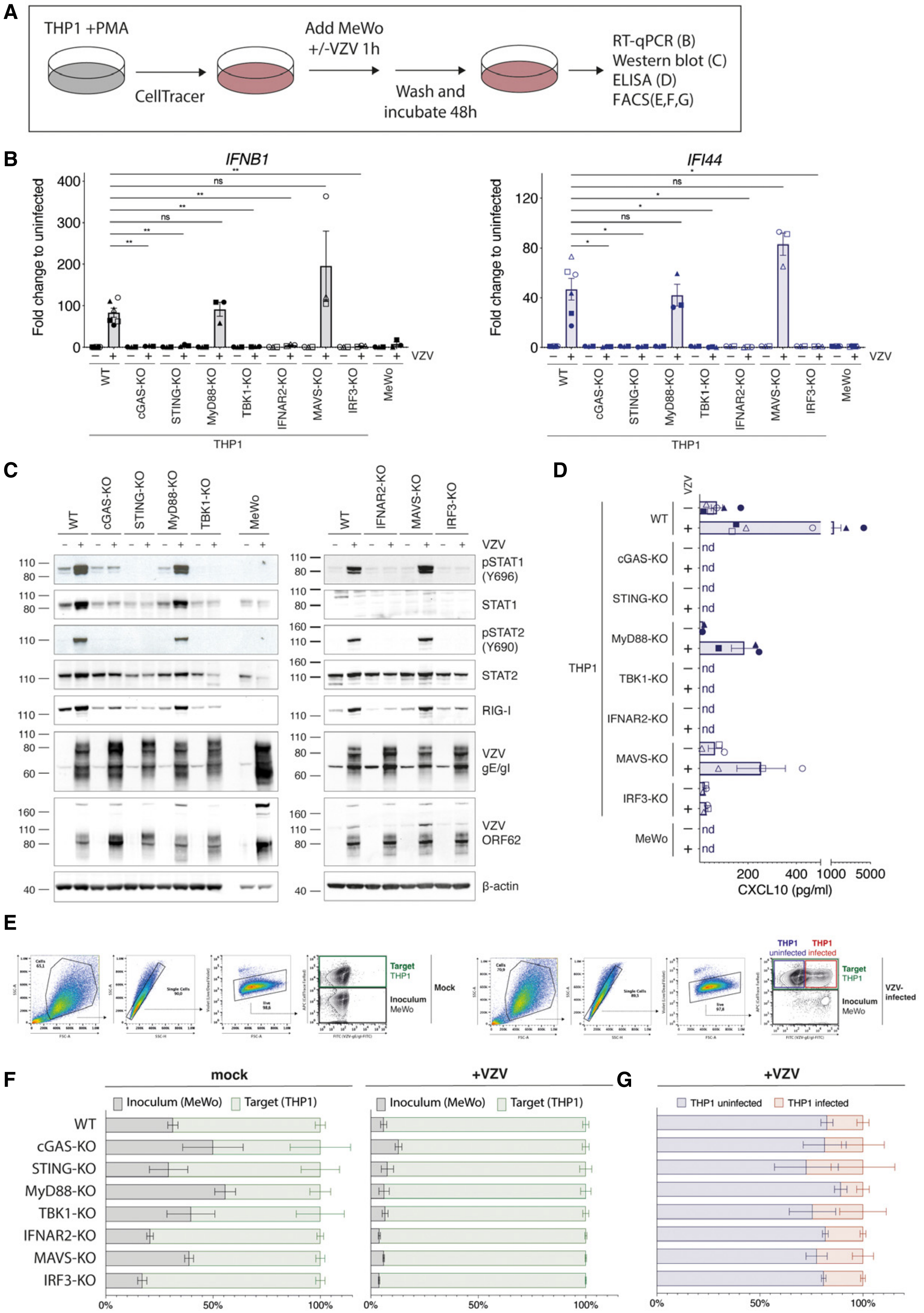

Figure EV1.

© 2022 The Authors

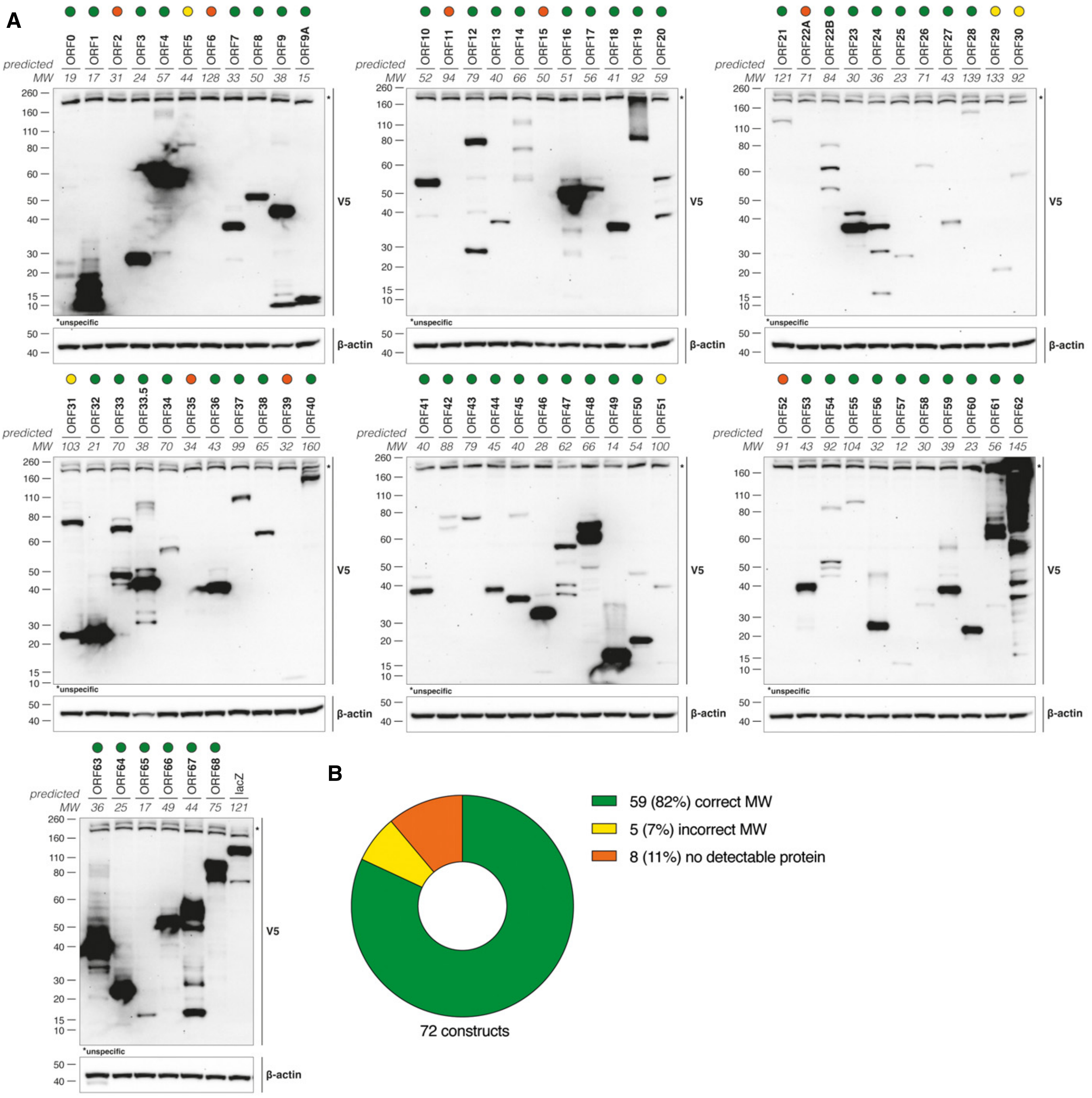

Figure EV2. VZV ORF expression library. Related to Fig 2.

A HEK293T cells were transiently transfected with individual VZV ORF expression constructs. The next day, cell lysates were subjected to immunoblotting. Ectopically expressed proteins were detected with an antibody against the V5 tag. The predicted molecular weight (MW) is indicated in kDa. Coloured circles highlight VZV proteins expressed at the predicted MW (green), expressed at a wrong MW (yellow) or not detectably expressed (orange).

B Summary of the data in (A).

Data information: Data in panels (A) and (B) are from one experiment.

 © 2022 The Authors

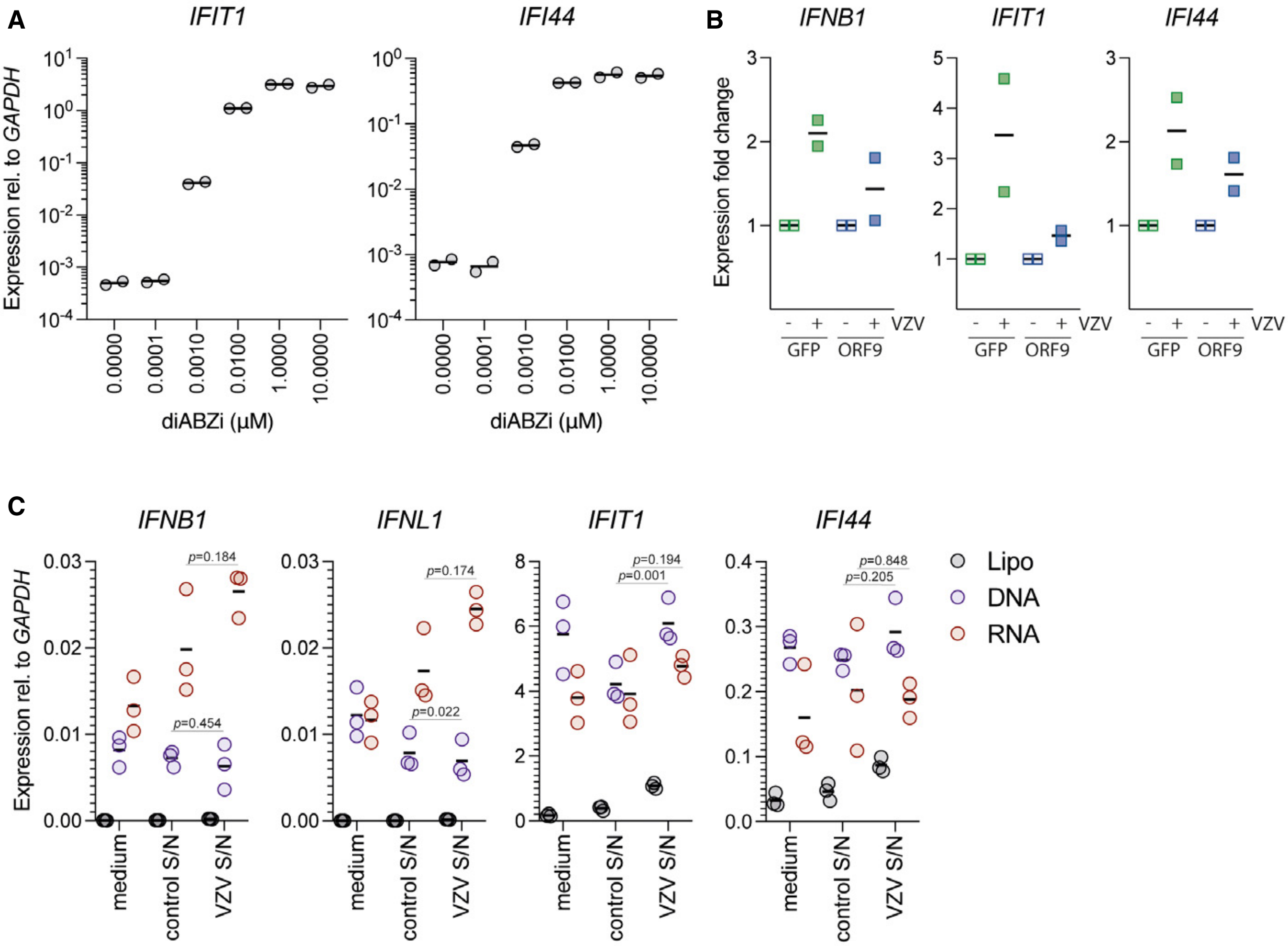

Figure EV3. ORF9 blocks IFN and ISG induction in VZV-infected cells. Related to Fig 2.

A THP1 cells were treated with the indicated concentrations of diABZi. After 24 h, expression of the indicated ISGs was determined by RT–qPCR. Data are relative to *GAPDH*.

B THP1 monocytes stably transduced with either VZV ORF9 or GFP were VZV or mock infected by co-culture with VZV-infected or uninfected MeWo cells. Expression of *IFNB1*, *IFIT1*, and *IFI44* was assessed by RT–qPCR. Expression fold change relative to uninfected cells is shown.

C THP1 cells were treated with PMA and were then transfected with *E. coli* DNA (DNA), the RIG-I agonist Neo$^{1\text{-}99}$ *in vitro* transcribed RNA (RNA) or, as control, treated with transfection reagent alone (lipofectamine 2000, Lipo). These stimulations were performed in fresh M10 medium (medium) and in conditioned medium collected from uninfected (control S/N) or VZV-infected (VZV S/N) MeWo cells. After 24 h, expression of the indicated IFNs and ISGs was determined by RT–qPCR. Data are relative to *GAPDH*.

Data information: Data in (A) are technical duplicates from a single experiment (horizontal bars show means). Panel (B) shows pooled data from two repeats, where each data point represents an independent biological experiment (horizontal bars show means). In (C), data are pooled from three biological repeat experiments each performed in technical duplicate (horizontal bars show means). Statistical analysis was paired *t*-tests.

Figure EV4. ORF9 phase-separates with DNA. Related to Fig 5.

A–D Phase separation of cGAS and DNA was analysed as described in Fig 5C and D using 10 µM or 5 µM ORF9 or ORF9-N.

Data information: Data are representative of three experiments.

© 2022 The Authors

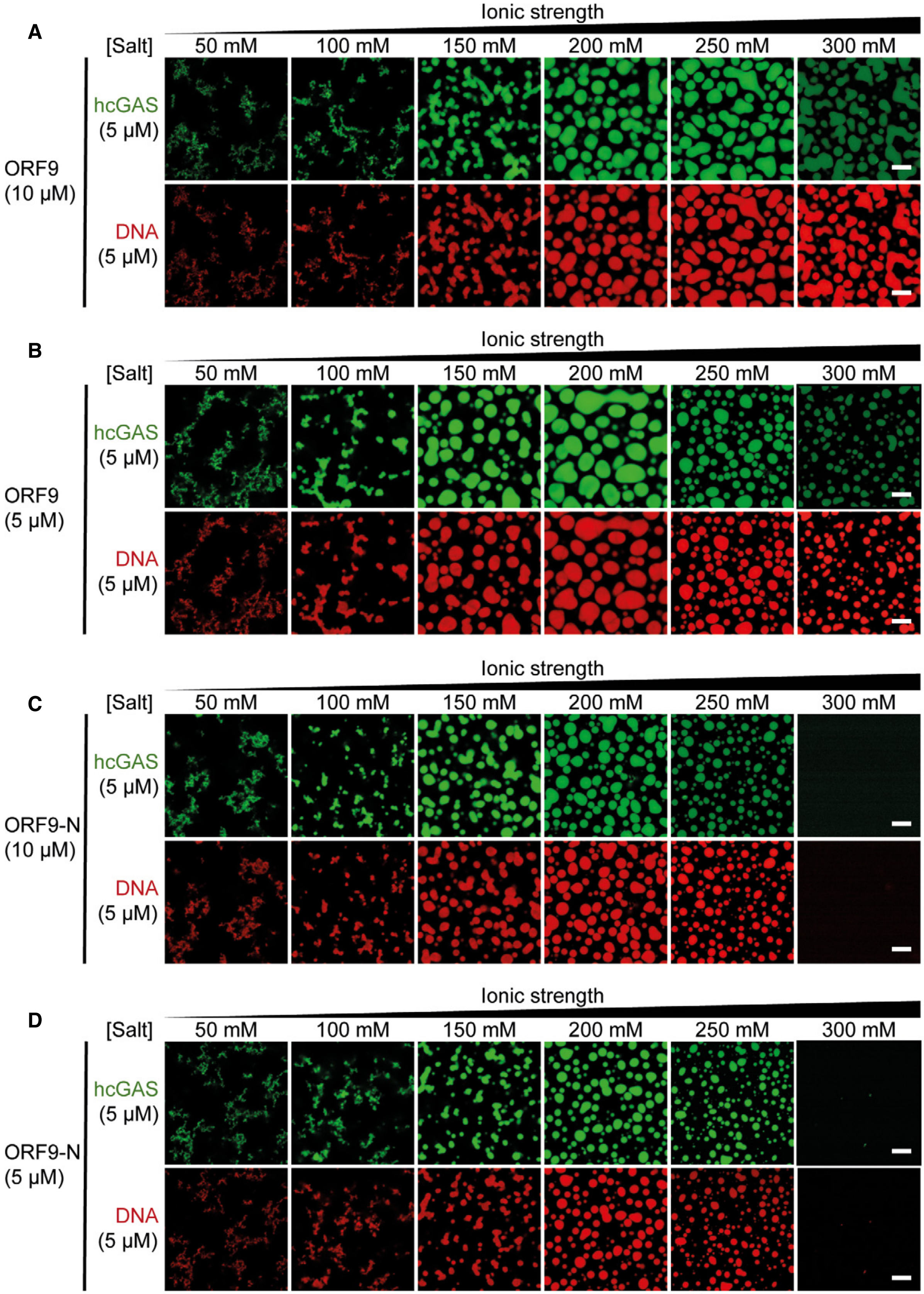

Figure EV4.

 © 2022 The Authors
