# Supplementary material for: Varicella‐Zoster virus ORF9 is an antagonist of the DNA sensor cGAS
Source: EMBO J. 2022 Jun 7;41(14):e109217. doi: 10.15252/embj.2021109217 (PMC9289529; doi:10.15252/embj.2021109217)
Supplement: Supplementary file 6 — Source Data for Figure 4 [file EMBJ-41-e109217-s007.pdf]

Figure 4 - full blots

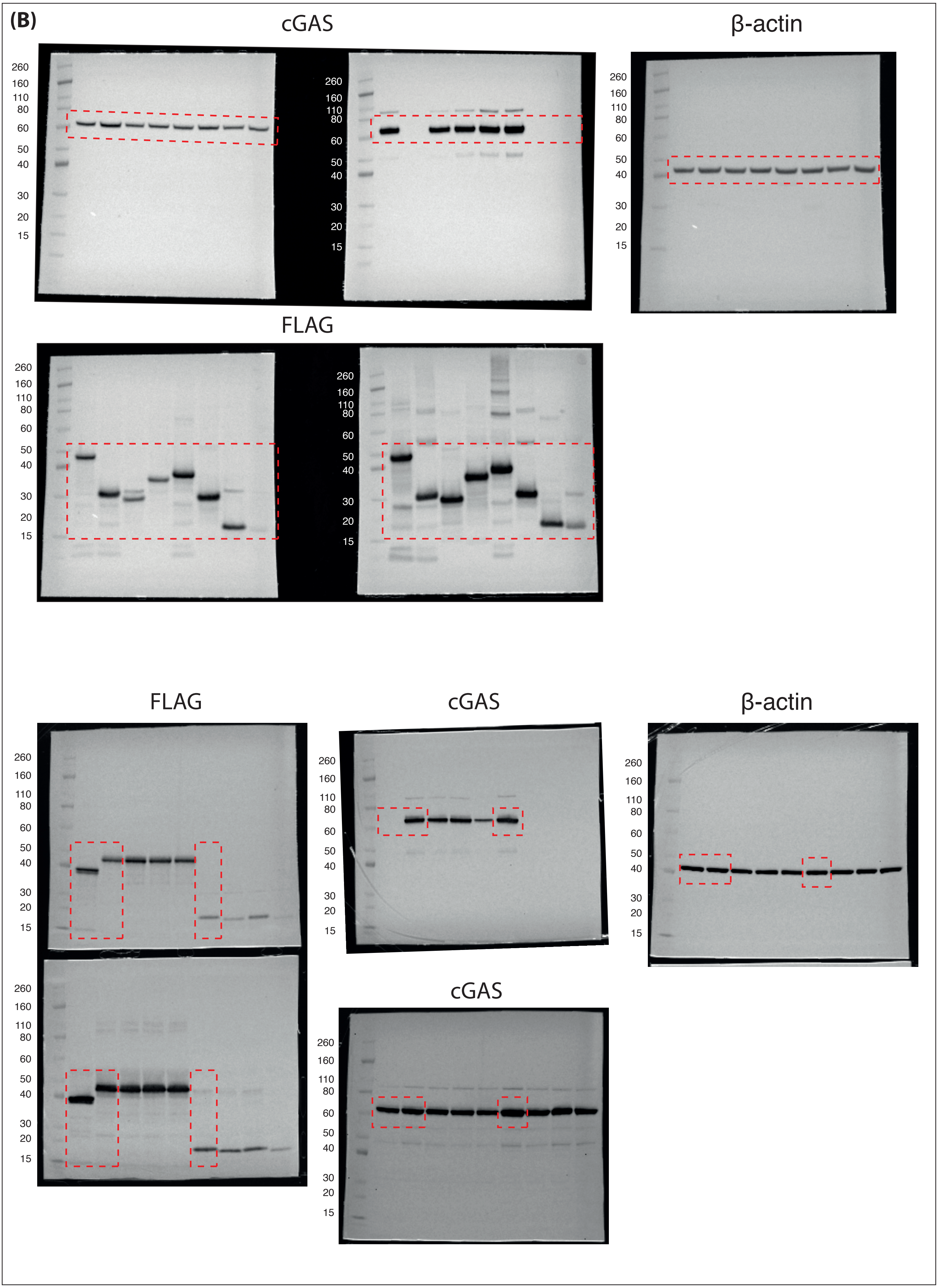

(D)

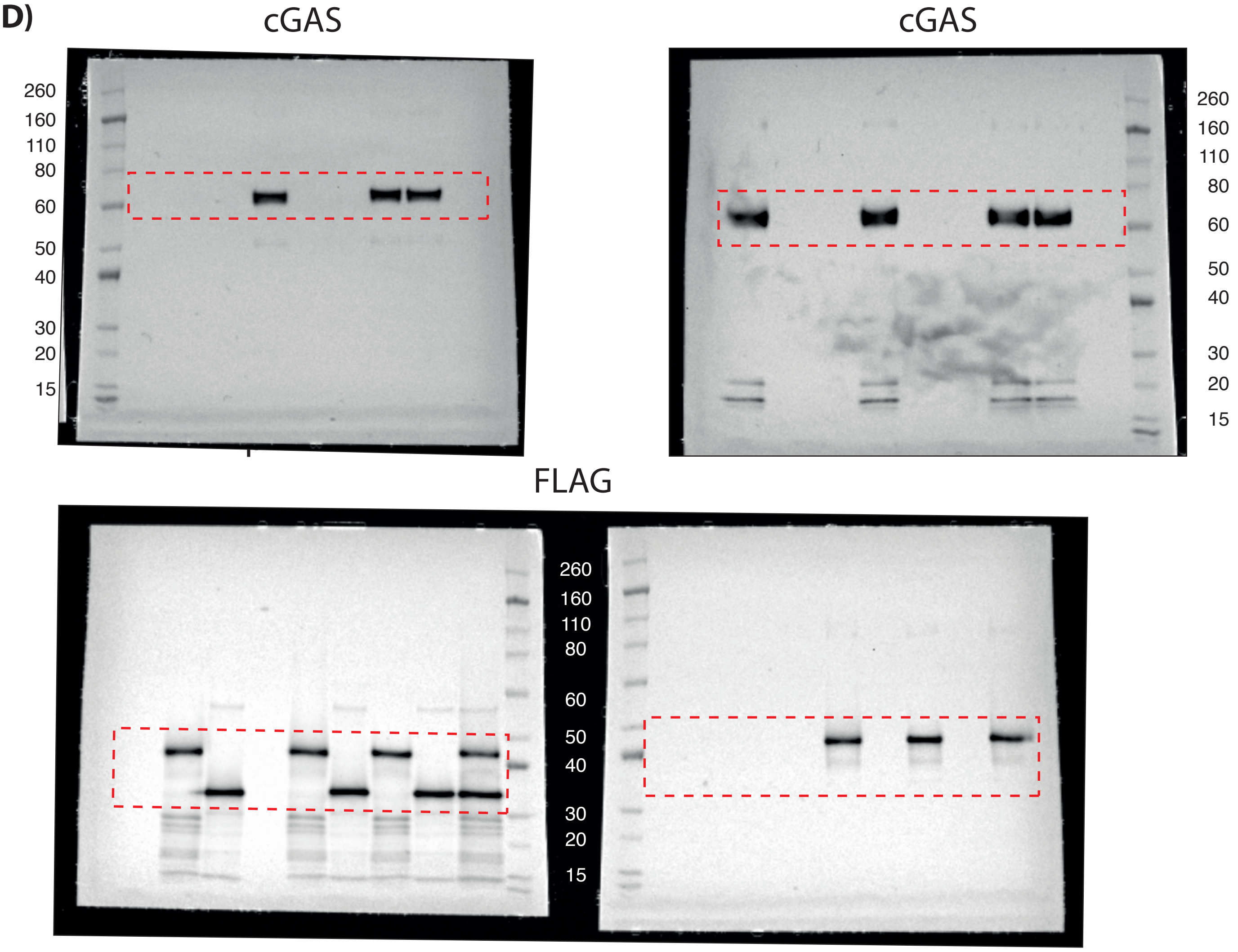

(E)

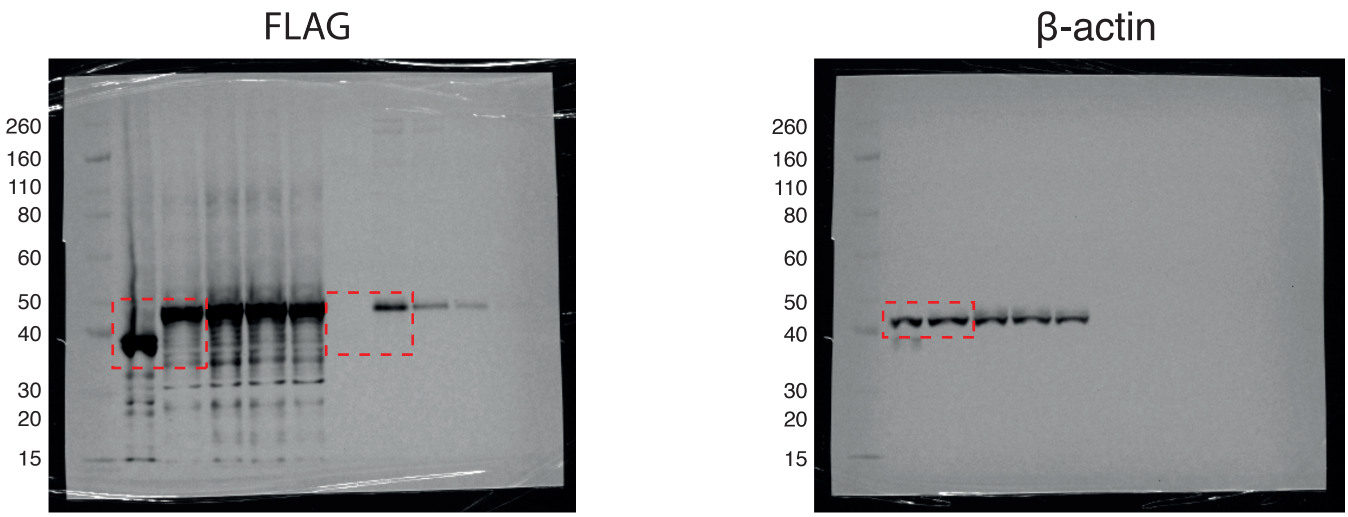

(F)

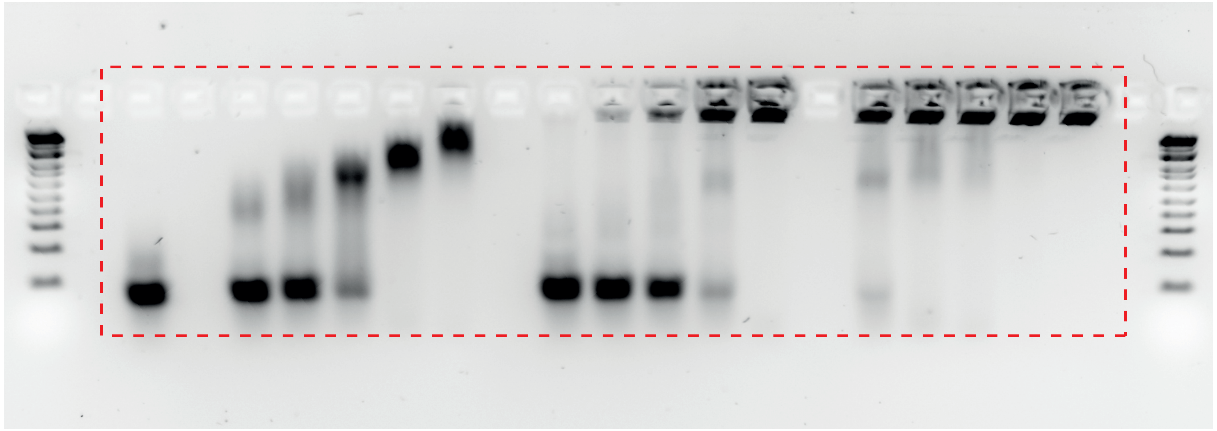

To visualise molecular weight markers, full bots shown here are overlays of bright field (membrane) and chemoluminescence images. Figures show only the chemoluminescence signal form the areas outlined by red dashed lines
