# Supplementary material for: Varicella‐Zoster virus ORF9 is an antagonist of the DNA sensor cGAS
Source: EMBO J. 2022 Jun 7;41(14):e109217. doi: 10.15252/embj.2021109217 (PMC9289529; doi:10.15252/embj.2021109217)
Supplement: Supplementary file 8 [file EMBJ-41-e109217-s002.pdf]

THE EMBO JOURNAL

Varicella-Zoster Virus ORF9 Is an Antagonist of the DNA Sensor cGAS

Jonny Hertzog, Wen Zhou, Gerissa Fowler, Rachel Rigby, Anne Bridgeman, Henry Blest, Chiara Cursi, Lise Chauveau, Tamara Davenne, Benjamin Warner, Paul Kinchington, Philip Kranzusch, and Jan Rehwinkel
DOI: 10.15252/embj.2021109217

Corresponding author(s): Jan Rehwinkel (jan.rehwinkel@imm.ox.ac.uk)

Review Timeline:

Transfer from Review Commons:	15th Jul 21
Editorial Decision:	21st Jul 21
Revision Received:	18th Feb 22
Editorial Decision:	28th Mar 22
Revision Received:	6th May 22
Accepted:	9th May 22

Editor: Karin Dumstrei

Transaction Report: This manuscript was transferred to The EMBO Journal following peer review at Review Commons.

Review COMMONS

(Note: With the exception of the correction of typographical or spelling errors that could be a source of ambiguity, letters and reports are not edited. Depending on transfer agreements, referee reports obtained elsewhere may or may not be included in this compilation. Referee reports are anonymous unless the Referee chooses to sign their reports.)

Dear Jan,

Thank you for submitting your manuscript to The EMBO Journal. This study was reviewed at Review Commons and transferred to The EMBO Journal. I have now had a chance to look at the manuscript, referee reports and your point-by-point response.

I appreciate that your analysis reporting a role of the cGAS/STING DNA sensing pathway in type I interferon induction during a VZV infection. The analysis also shows that the VZV ORF9 binds and antagonizes cGAS. I find the analysis insightful. I also appreciate your response to the referees' concerns and see that you can add data to address the majority of the raised concerns. I also understand the reasoning for the experiments that you will not be able to do.

Given this, I would like to invite you to submit a revised manuscript that addresses the concerns raised as proposed in your outline.

Let me know if we need to discuss anything further.

I have attached a document with instructions on how to prepare the revised version. Please pay attention to the parts on the Data Availability Section and figure legends.

With best wishes

Karin

Karin Dumstrei, PhD
Senior Editor
The EMBO Journal

Guide For Authors: https://www.embopress.org/page/journal/14602075/authorguide

Revision to The EMBO Journal should be submitted online within 90 days, unless an extension has been requested and approved by the editor; please click on the link below to submit the revision online before 19th Oct 2021:

Response to reviewers' comments

EMBOJ-2021-109217 | [RC-2021-00768] [REV]

Varicella-Zoster Virus ORF9 Is an Antagonist of the DNA Sensor cGAS

Jonny Hertzog, Wen Zhou, Gerissa Fowler, Rachel E. Rigby, Anne Bridgeman, Henry T. W. Blest, Chiara Cursi, Lise Chauveau, Tamara Davenne, Benjamin E. Warner, Paul R. Kinchington, Philip J. Kranzusch, and Jan Rehwinkel

The authors would like to thank all reviewers, the *Review Commons* team and the editors at *The EMBO Journal* for carefully evaluating our manuscript and for all the helpful comments and suggestions for improvement. We were delighted to hear that we were invited to submit a revised version of our manuscript.

All reviewers have highlighted that our conclusions are well-supported by the experimental data presented. Furthermore, it was pointed out that our findings are of great relevance to the virology and innate immunology communities and that our description of cGAS inhibition is important in the context of intense research on cGAS modulation in human disease. With the additional experiments and changes to the manuscript outlined below, we believe that our work constitutes a significant contribution to these fields and will be of interest to many researchers. We therefore hope our manuscript is a good candidate for publication in *The EMBO Journal*.

New or revised text is highlighted in red in the revised manuscript files. To reflect their contributions to the revisions, Henry T. W. Blest and Anne Bridgeman were included in the author list and Gerissa Fowler was moved to position 3 in the author list. Benjamin E. Warner and Paul R. Kinchington were included as authors for developing and providing ORF9-DHFR virus.

Point-by-point reply

Response to reviewer #1:

In this work, Hertzog et al study the role of the cGAS-STING pathway in the restriction of VZV infection. The authors first demonstrate in co-culture assays that the cGAS-STING-TBK1-IRF3-IFNAR2 axis is critical for the induction of an IFN response upon infection. They then constructed a large library of 59 ORFs from VZV which they tested in cGAS-STING overexpressing IFNb-luc reporter cells to identify modulators of the pathway. This led to the identification of ORF9 as a potent inhibitor of cGAS-STING. The authors next showed that cGAS but not STING could be pulled down with ORF9 including when expressed from a recombinant virus. ORF9 interacted with cGAS in vitro, and this was due to the region 141-249 of ORF9. The authors next looked at whether ORF9 was impacted cGAS, which was not through competitive binding to DNA, but rather through inhibition of the enzymatic activity of cGAS and CGAMP synthesis.

Overall the paper is very clear and the claims are very well supported, but an additional model supporting the role of cGAS in viral restriction (and the effect of ORF9) would help strengthen the biological significance of the findings of the manuscript.

We would like to thank the reviewer for their constructive assessment of our manuscript and positive comments.

Major comments:

1) The paper entirely relies on co-culture models to assess the role of cGAS on infection. This review notes that MeWo cells actually have cGAS, but lack STING (see PMID: 27680683; the pathway seems functional in these cells since treatment with aza restores function). It would be ideal to show that MeWo cells expressing STING, when co-cultured with infected MeWo, actually restrict the replication (with concurrent increased IFN response), compared to WT cells.

We thank the reviewer for their suggestion. We agree that investigation of VZV sensing in MeWo cells in the context of a functional cGAS/STING pathway would add nicely to the manuscript. To address this point, we reconstituted STING in MeWo cells through lentiviral transduction. We then inoculated these cells with VZV by co-culture with infected MeWo cells and assessed innate immune sensing by RT-qPCR (Fig R1A). We also confirmed infection by RT-qPCR (Fig R1B) and expression of STING by immunoblotting (Fig R1C). STING was successfully expressed in MeWo by this approach and the cells were infected with VZV (Fig R1B,C). However, different ISGs and *IL6* mRNA were not induced upon VZV infection (Fig R1A). This indicates that MeWo cells expressing STING do not detect VZV and consequently do not restrict VZV. We speculate that this is due effective evasion of innate sensing by VZV in this cell type. Please also see our response to reviewer 3 (major point 1).

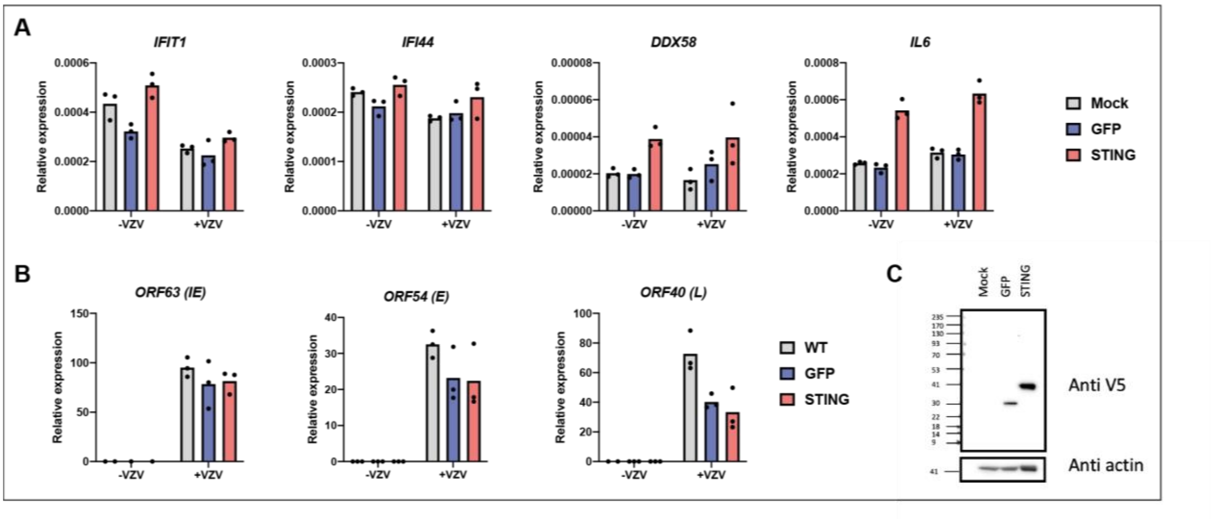

Fig R1: MeWo expressing STING do not detect VZV.
MeWo cells were transduced with lentivirus expressing C-terminally V5-tagged GFP or STING. Unmodified MeWo cells (mock) and GFP or STING expressing cells were then infected with VZV by co-culture with VZV-infected MeWo cells (+VZV) or as control were co-cultured with uninfected MeWo cells (-VZV). After 1h, inoculum MeWo cells were washed away. After 24h, RNA was extracted for RT-qPCR analysis. **(A)** RT-qPCR analysis of *IFIT1*, *IFI44*, *DDX58* and *IL6* mRNA expression. Data are shown relative to *GAPDH*. **(B)** RT-qPCR analysis of VZV ORF63 (immediate early (IE) gene), ORF54 (early (E) gene), and ORF40 (late (L) gene) transcripts. Data are shown relative to *GAPDH*. **(C)** Western blot analysis of GFP-V5 and STING-V5 expression.

2) The authors do not directly show the role of ORF9 in the facilitation of infection. Since mutating this protein in the virus may impact infection, the authors should test whether co-culture of their THP-1 cells overexpressing ORF9 with infected MeWo dampens ISG induction in THP-1 cells (the result should be intermediate to the ISG inhibition seen in cGAS KO cells in Figure 1E).

We agree with the reviewer that it would be interesting to study the role of ORF9 in the context of virus infection. We addressed this point in two ways:

1) We infected ORF9-THP1 and GFP-THP1 cells with VZV by co-culture as per the reviewer's suggestion. As shown in the new Fig 2G, ORF9 dampened *IFNB1* and ISG induction. As predicted by the reviewer, the effect was intermediate when compared to the effect of genetic cGAS KO.
2) We used a recombinant virus in which ORF9 is fused to an *E.coli* DHFR degron motif. In this setting, withdrawal of a stabilising compound (trimethoprim, TMP) leads to degradation of the ORF9 protein. We infected THP1 cells for 24h with the ORF9-DHFR virus in the presence of TMP to establish the infection. TMP was then withdrawn and IFN activation was measured by RT-qPCR. In addition, ORF9 protein levels were assessed by immunoblotting. In the absence of ORF9 protein, VZV infection led to higher IFN induction compared to cells in which ORF9 was TMP-stabilised. These new data, included in Fig 2H-K, strongly support that in infected cells, ORF9 antagonises recognition of the virus and leads to overall reduced IFN levels.

3) The experiments relying on ORF9 THP-1 should validate that downstream STING activation of these cells results in similar ISG induction as in GFP cells (in Figs2 and 6) - to confirm that inhibition of the pathway is selective to cGAS. This can easily be achieved using the diABZi human STING agonist (now available from selleckchem).

We thank the reviewer for their suggestion. To assess STING activation in the context of ORF9 expression, we stimulated ORF9-THP1 or GFP-THP1 control cells with diABZi, a potent STING agonist. We used a dose that induces a sub-maximal response (see new Fig EV3A). We find that the response to diABZi was similar in GFP and ORF9 expressing cells, indicating that ORF9 did not inhibit STING. These new data are shown in Fig 2F in the revised manuscript.

Minor Comments:

1) the authors need to be more consistent about the way the data is presented between figures. This reviewer found it confusing that some figures use symbols in graphs to represent independent experiments, but in other figures they represent technical/biological replicates from a single experiment (e.g. Fig 1 shows pooled independent experiments, but Fig 2 only shows representative experiments, and Fig 6 is a mix - panel G is very confusing with the legend stating pooled from three independent experiments but n=2? - and panel E is in fact just one experiment if I am correct?).

We apologise if the data presentation was confusing. By showing individual data points in all graphs we aimed to provide maximum transparency. In the revised manuscript, we will clarify the way the data is presented and make it more consistent where possible. The specific example of Fig 6 represents a mistake in the legend, which we corrected in the revised manuscript.

2) The sequences of the guide RNA used for the CRISPR/Cas9 should be provided.

We apologise for this omittance and added the sequences to our revised manuscript.

3) It was not clear to this review why the Figure S3 was not in fact Figure 1 - since it is where the data about the implication of the cGAS-STING axis in infection is first made.

We initially assessed cell-autonomous VZV sensing by co-culturing a panel of THP1 KO cells with infected MeWo cells. As pointed out by the reviewer, these experiments indicated a role of cGAS/STING in sensing of VZV. However, we prefer to keep these data in the 'Expanded View' content because these THP1 KO cells were obtained from various sources. Furthermore, in these experiments, co-cultured cells were harvested for RNA extraction and thus represented a mixture of THP1 cells and inoculum MeWo cells. We cannot rule out that extracted THP1 RNA was "diluted" with MeWo RNA, and, importantly, the proportions of MeWo and THP1 RNA differed between samples (see flow cytometry data on proportions of MeWo and THP1 cells in co-culture experiments in the new Fig EV1E,F). To circumvent these issues, we generated cGAS and MAVS KO cell lines isogenic to our WT cells. In addition, we conceived a novel way of infecting cells with VZV in which inoculum and target cells are spatially separated using a transwell approach. The data obtained using this experimental setup confirmed a critical role of cGAS in recognition of VZV infection. We believe it simplifies the understanding for the reader to focus on these data demonstrating a role of cGAS in Fig 1, with the wider dataset linked as Fig EV1.

4) Since Fig 2B shows the same a Fig 2A, what is really the point of A? Also, please clarify that you only tested the 59 validated ORFs in the screen data. Please also highlight ORF9 in Figure 2B.

Fig 2A shows a representative screen experiment in which data points signify three technical replicates. We included these data to show the distribution of values in a single experiment. This screen experiment was performed three times and ORFs were ranked in each experiment by their ability to block reporter activation. The results of all three independent screens are shown in Fig 2B, where the first row is data from the experiment also shown in (A). With this format of data presentation, we aimed to be as transparent as possible with regards to the primary data. We have added "(A)" to the first row of the heatmap in Fig 2B so emphasise that these data come from the exemplary experiment shown in (A).
Our VZV ORF library encompasses 72 constructs, which were all tested for expression by immunoblotting (Fig EV2). Of these, 68 were included in the cGAS/STING screening experiments. This is because we managed to clone ORFs 9A, 22A, 22B, and 42 only after the screening experiments were performed. However, all available plasmids were included in the screen, not just the 59 that showed correct expression.

5) Figure 4D states lysates - aren't these in vitro studies of recombinant proteins?

Indeed, recombinant proteins were used in Fig 4D. We thank the reviewer for spotting the mistake and corrected it.

6) In discussion about TLR9 in DCs, please note that the paper referenced used the A151 ODN, which was also shown to inhibit cGAS - PMID: 29215161 -(so cGAS could also have been involved in their experiments).

We thank the reviewer for this suggestion and added this to the discussion in our revised manuscript.

7) As pointed out in the discussion - line 441-442 - it could be a good idea to tweak a bit better the overall concept of the paper in the abstract (which can indeed be confusing): cGAS participates to antiviral restriction of VZV, but that ORF9 helps partially evade cGAS sensing.

We thank the reviewer for their suggestion and adjusted the wording of the abstract accordingly.

Reviewer #1 (Significance (Required)):

This manuscript constitutes an important body of work for our understanding of viral evasion of innate immune responses by VZV. The authors generated novel tools (such as the large ORF library) and protocols which will significantly advance research on VZV.
The findings could be useful in designing better adjuvants for vaccination (one could argue that ORF9 may antagonise sensing of the inactivated virus by cGAS, but that STING agonists may help by-pass this).

For the growing field of cGAS inhibition, this paper provides yet another angle to help modulate cGAS activation - and while further experiments are warranted to define how ORF9 inhibits cGAS enzymatic activity, the findings about ORF9 can inform the design novel molecules/therapies.

It should be noted that I am not an expert on VZV, but rather have extensive expertise about cGAS-STING sensing, and inhibition.

Referee Cross-commenting

Comments from reviewer 3:

> "Thus, the physiological relevance of THP1 cells as model system is rather over-stated in the introduction/results, and only somewhat qualified in the discussion":

reviewer 1 agrees with this comment; using the MeWo experiment proposed would help address this point.

> "the authors could show that THP1 responses to DNA or RNA are not altered in the presence of conditioned medium from infected MeWo cells"

reviewer 1 would argue that using a STING agonist in the presence of co-culture would help address this (since MeWo do not have STING)

> "Data in Fig. 2 seem to be solely technical duplicates or triplicates - this is not really sufficient. "

reviewer 1 had raised a similar point.

> "The authors could also test the function of ORF9 upstream of downstream of STING activation"

yes, using human STING agonist (commercially available).

> "The authors could try to measure inhibition of cGAMP production also in cells (e.g. THP1 cells stably expressing ORF9), using LC-MS or cGAMP ELISA. This would further show that the effect that the authors describe (mainly observed in vitro) indeed also happens in living cells,"

This is a good point (though the reviewer does not say whether they want this in the context of infection or just DNA transfection?), but would not be useful in the context of MeWo co-culture since the MeWos are likely to make cGAMP themselves (since they have cGAS). It may be simpler to show that ORF9 THP-1 cells transfected with DNA make less cGAMP.

> "While it will probably not be possible to construct deletion viruses to demonstrate the effect of ORF9 during infection, it might be conceivable to use ORF9 RNAi, in combination with the quantification of early responses (within the first hours of infection) prior to viral replication etc"

reviewer 1 would object that targeting ORF9 could itself alter the viral replication, and siRNAs/lipids can also activate immune responses, thus confounding the results. It seems much simpler to use infection of ORF9 THP-1 cells as suggested in my review.

Response to reviewer #2:

The manuscript identifies the VZV ORF9 protein as a specific inhibitor of cGAS. The group shows that cGAS signalling is activated in response to VZV infection of THP-1 cells leading to the production of antiviral cytokines/chemokines including type I interferon and CXCL10. They also report that VZV ORF9 antagonises this pathway by binding directly to cGAS, as well as DNA, blocking cGAMP production and thus inhibiting the downstream induction of type I interferon. This paper therefore increases our understanding of VZV immune evasion. The group utilises a novel method to establish a cell-associated infection (necessary when working with VZV in vitro) that will be of great interest in the field (although a further manuscript will elaborate on this method). ORF9 was identified as a key antagonist of cGAS using a broad screen of VZV open reading frames and to do this the group constructed a library of plasmids containing 59 individual VZV ORFs which could also be useful for others in the field (although other groups have generated similar libraries in the past). They then showed that ORF9 directly binds to cGAS and DNA using co-immunoprecipitation experiments and mapped the regions responsible for these interactions.

Major comments:

- Are the key conclusions convincing?

Yes, the key findings are well supported by the experimental data

We thank the reviewer for their summary of our manuscript and the positive assessment.

- Should the authors qualify some of their claims as preliminary or speculative, or remove them altogether?

In the first section of the results the title claims that "The type I interferon response to VZV requires the DNA sensor cGAS". It would be more accurate to say that "Induction of type I interferon in response to VZV infection of THP-1 cells requires cGAS". I think it is important clarify this as other cell types may possess other sensors (as noted in line 407) that can induce type I interferon during VZV infection. This also should be corrected in line 401/402 in the discussion. Likewise, line 404 should read 'identify cGAS as a DNA sensor for VZV' rather than 'the'

Additionally, there as remarks in the text about the 'type I IFN response to VZV infection' that should read the induction of type I IFN in response to VZV infection e.g. line152/153 and 175.

We thank the reviewer for the suggestion to clarify some of the wording in our results and conclusions and changed the text accordingly.

- Would additional experiments be essential to support the claims of the paper? Request additional experiments only where necessary for the paper as it is, and do not ask authors to open new lines of experimentation.

No

- Are the suggested experiments realistic in terms of time and resources? It would help if you could add an estimated cost and time investment for substantial experiments.

N/A

- Are the data and the methods presented in such a way that they can be reproduced?

The manuscript utilises a new method of establishing a cell-associated infection with VZV that will be of great interest and use for the field. Whilst I note that another manuscript detailing this method is in preparation, without being able to see the experiments used to validate this method, it is somewhat difficult to know how robust this set up is. Perhaps the authors could add a comment on the purity of the THP-1 cells collected at the end of the co-culture?

During development of the transwell assay we studied the purity of the recovered THP1 cell population. For this purpose, inoculum MeWo cells at the bottom of the transwell were labelled with a fluorescent tracer dye prior to addition of THP1 cells to the top of the insert. After 48h, cells from both sides of the transwell membrane were recovered and analysed by flow cytometry (Fig R2 below). Within the THP1 cell population, around 0.3% of cells were positive for the tracer dye, i.e. very likely represented inoculum MeWo cells. The recovered THP1 cell populations used for analysis were thus >99% pure.

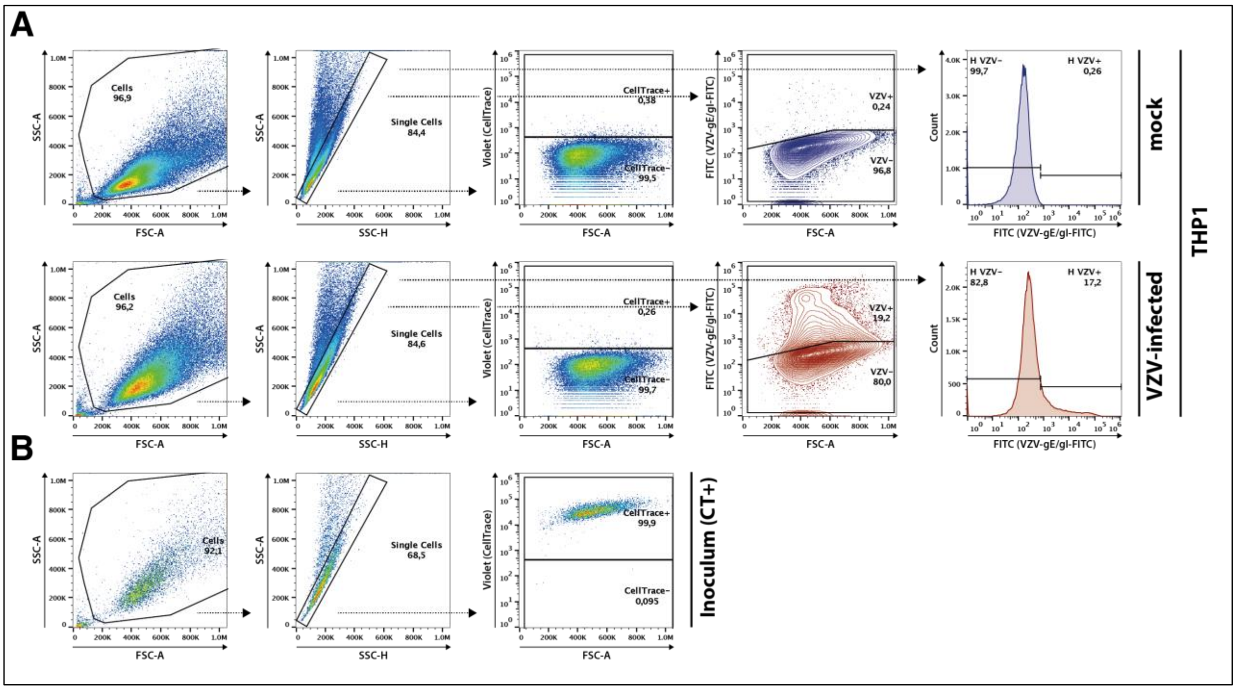

Fig R2: Transwell VZV-infected THP1 cells were >99% pure.

Uninfected or VZV-infected MeWo cells were seeded on the bottom side of a transwell insert and allowed to adhere overnight. Inoculum MeWo cells were then labelled with a tracer dye (CellTrace Violet). Subsequently, THP1 cells were added in medium containing PMA to the upward-facing side of the transwell insert. After 48h, THP1 cells from the top of the insert and MeWo inoculum cells from the bottom of the insert were recovered and processed for analysis by flow cytometry as described in the methods section of the manuscript. Panel (A) shows flow cytometry analysis of uninfected and VZV-infected THP1 cells. Panel (B) show analysis of CellTrace-labelled MeWo cells used as inoculum for the VZV-infected sample in Panel (A).

- Are the experiments adequately replicated and statistical analysis adequate?

Yes

Minor comments:

1) With regards to the initial VZV THP-1 co-culture (S3), details of labelling the THP-1s are missing from the methods section. In addition, were some of the cells subject to flow cytometry to determine the purity

of the THP-1 culture? Presumably this was the reason for labelling them, otherwise this is unnecessary. Also was the percentage of VZV positive THP-1 cells determined?

Thank you for spotting this omission. We added these data and methods to the revised manuscript (Fig EV1E,F and Methods).

2) Fig1D. qRT-PCR results show relative gene expression, should be clarified that this is relative to WT infection presumably.

The data in Fig 1D show the expression relative to *GAPDH* as a housekeeping gene. No further normalisation was applied. We changed the figure legend to clarify.

3) Fig2 A and B. Figure B displays a heatmap with results from 3 independent experiments, are these the same 3 experiments represented in dot points for Fig 2A? The figure legend suggests this however, they don't seem to correlate with the heat map e.g. for ORF66 vs ORF4. Some clarification around this data would be helpful.

Reviewer 1 raised a similar point regarding the data in Fig 2A and B and we provide a detailed response above. In brief, Fig 2A shows a single experiment and data points represent technical replicates. The heatmap in Fig 2B shows the results of three independent biological repeats of the experiment shown in Fig 2A.

Reviewer #2 (Significance (Required)):

This work is a significant advancement in the VZV field, in both identifying a new immune evasion strategy of the virus (ORF9 as an antagonist of cGAS) as well as describing a new method to conduct cell-associated infection with VZV- a longstanding issue which has hampered progress in the field.
It builds on previous findings by Kim et al., (2017) who previously recognised the role of STING in the induction of type I IFN in response to VZV infection and adds cGAS as a sensor of VZV infection. Previous work by Soren Paludan and Trine Mogensen (Ogunjimi et al., 2017) identified RNA-pol III as a sensor of VZV infection in immune cells, and more recently Steain et al., 2020 (PLoS pathogens) shows that VZV ORF20 interacts with the nucleic acid sensor ZBP1 and that ZBP1 can inhibit VZV replication by inducing cell death.

The work would be of interest to others in the VZV field, as well as the broader herpesvirus community.

My area of work is in VZV-host interactions. I do not have sufficient expertise to evaluate the phase separation experiments.

Response to reviewer 3:

Reviewer #3 (Evidence, reproducibility and clarity (Required)):

This manuscript describes VZV ORF9 as antagonist of the DNA sensor cGAS. The authors show that the cGAS-STING-IRF3-interferon signalling axis is activated during VZV infection of THP1 monocytes, and identify ORF9 in a screen of viral ORFs that inhibit cGAS/STING induced activation of an irf3-dependent promoter. The authors show that ORF9 interacts with cGAS and with DNA, and this leads to the inhibition of cGAMP production in vitro.

Overall, the data are presented in a clear and logical fashion, and experiments are largely well controlled.

We thank the reviewer for the summary of our manuscript and their positive comments regarding the quality of our data.

Major points:

1) While human immortalised THP1 monocyte/macrophage-like cells are a well-studied model cell system for innate immune sensing, VZV usually replicates in skin cells, T cells and neurons. Thus, the physiological relevance of THP1 cells as model system is rather over-stated in the introduction/results, and only somewhat qualified in the discussion. To generalise their findings on the importance of the cGAS-STING pathway in VZV detection, they could perform a few key experiments in relevant human cell types (including primary cells) using CRISPR pools or RNAi.

We agree with the reviewer that studying the cell-intrinsic innate immune response in other cell types relevant to VZV replication would be informative. Our choice of THP1 cells was motivated by the fact that these cells are a well-established model system for cGAS-STING DNA sensing, as pointed out by the reviewer. To address the reviewer's point, we started by infecting HaCaT cells, a line of spontaneously immortalised keratinocytes. We chose these cells as we had previously generated HaCaT cGAS-KO cells (unpublished). Unfortunately, co-culture of HaCaT cells with VZV-infected MeWo cells did not result in induction of an IFN response compared to our control (HaCaT + uninfected MeWo cells) (Fig R3A). We then tested primary keratinocytes (Fig R3C) and primary fibroblasts (Fig R3E), with the intention of interrogating cGAS-dependency using a cGAS inhibitor. Much like the situation in HaCaT cells, however, VZV infection did not induce IFN responses in these cells (Fig R3C,E). In all three types of cells, we validated VZV infection by RT-qPCR (Fig R3B,D,F).
In summary, we have regrettably been unable to establish a cell system other than THP1 cells in which VZV induces an IFN response and could therefore not validate cGAS-dependency in another cell type. It is likely that VZV, much like other large DNA viruses, encodes multiple antagonists of innate immunity. Indeed, ORF47, ORF61, ORF62 and ORF63 are all known to block type I IFN induction or signalling (Sen *et al*, 2010; Vandevenne *et al*, 2011; Verweij *et al*, 2015; Zhu *et al*, 2011). We speculate that the exquisite sensitivity of THP1 cells to nucleic acid stimuli allows the induction of type I IFNs in VZV-infected cells despite viral expression of these antagonists. In less responsive cell types, viral antagonism likely outweighs innate signalling.
We hope the reviewer appreciates our efforts in this direction. Unfortunately, further experiments have not been possible with the time and resources available to us.

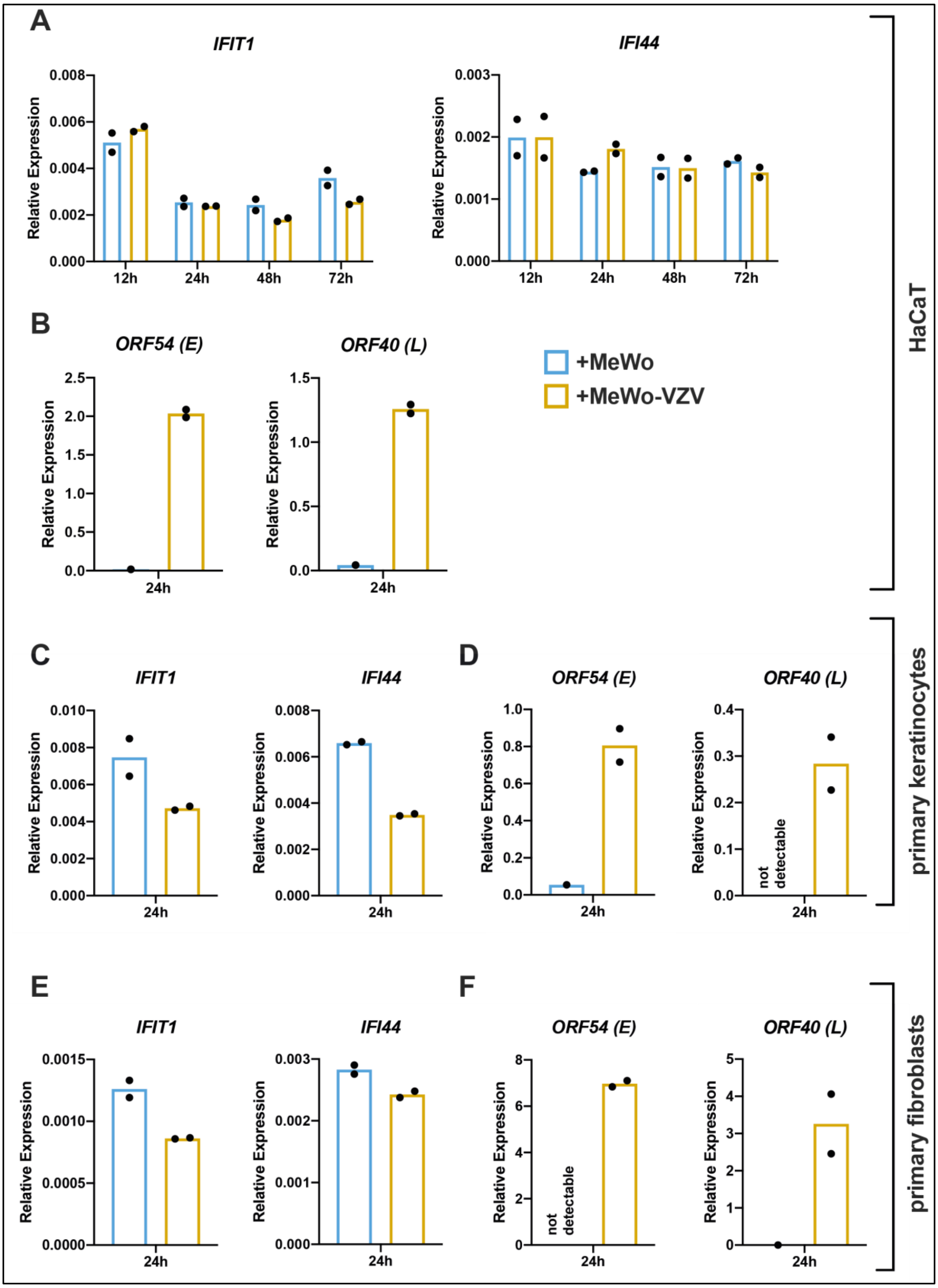

Fig R3: HaCaT cells, primary keratinocytes and primary fibroblasts do not detect VZV infection.
HaCaT cells **(A,B)**, primary keratinocytes **(C,D)** and primary fibroblasts **(E,F)** were infected with VZV by co-culture with VZV-infected MeWo cells or as control were co-cultured with uninfected MeWo cells. After 1h, inoculum MeWo cells were washed away. At the indicated time points, RNA was extracted for RT-qPCR analysis. **(A,C,E)** RT-qPCR analysis of *IFIT1* and *IFI44*. Data are shown relative to *GAPDH*. **(B,D,F)** RT-qPCR analysis of VZV ORF54 (early (E) gene) and ORF40 (late (L) gene) transcripts. Data are shown relative to *GAPDH*.

2) In order to rule out secondary effects (e.g. mediated by cytokines) during the co-culture or trans-well culture of infected MeWo cells with THP1 cells, the authors could show that THP1 responses to DNA or RNA are not altered in the presence of conditioned medium from infected MeWo cells.

We thank the reviewer for this suggestion. As requested, we stimulated THP1 cells by transfection with *E.coli* DNA or Neo[1-99] *in vitro* transcribed RNA (a RIG-I agonist) in the presence of medium from uninfected or VZV-infected MeWo cells. The transfection reagent lipofectamine and fresh medium served as controls. As shown in the new sup Fig EV3B, medium from VZV-infected MeWo cells had no appreciable impact on induction of *IFNB1*, *IFNL1* and ISG mRNAs. This result rules out an effect mediated by soluble factors.

3) In experiments such as those in Fig. 1D-E, uninfected or mock infected cells should be shown as control (this will provide additional information on the spread of data, and on the effects of e.g. cGAS or MAVS deletion on basal responses).

We added these data in the revised manuscript as requested in the updated Fig 1C, E, F and G. We did not add uninfected cells to Fig 1D as only viral transcripts were measured in this experiment.

4) It is notable that 4 of the IRF3 deletion clones show normal RNA-induced responses, and only 2 are impaired - while the response to DNA is abolished in all. The authors then pick one of the RNA- and DNA-sensing impaired clones as representative for further experiments to fit with the current paradigm of MAVS-IRF3-interferon induction. In my opinion, by pre-selecting the results the authors "want" to see, they may miss some interesting features on the requirement of IRF3 in RNA sensing - for instance have the authors checked for the presence of IRF7 or other IRFs in the closed which may compensate for RNA, but not DNA sensing at this relatively late time point, and which may be homologous enough for off-target effects? Or how else do the authors explain the difference observed between RNA vs DNA stimulation in the majority of their IRF3 deletion clones? Of note, this is not key for this manuscript in particular (which is concerned with DNA sensing and does not really touch on IRF3 per se) - but given the data is shown here, and possibly not in future manuscripts that make use of these cells, I think this is an important point. For this manuscript, the use of IRF3 deletion cells is not crucial.

We thank the reviewer for their observation that our IRF3-KO clones generated through CRISPR/Cas9 editing show different responses to RNA stimulation. We agree these data possibly hint at quite interesting biology in regard to downstream signalling from PRRs and type I IFN transcriptional activation. While we were intrigued by this phenomenon, we believe further investigations in this direction are beyond the scope of our study. Similarly, we did not have capacity to test protein levels of IRF7 or other IRFs as part of these experiments. For this manuscript, IRF3-deletion served as a positive control and contributed marginally to the conclusions drawn (as also pointed out by the reviewer).

5) The authors show only very few read-outs for innate immune induction by ORF9 (luciferase assays and IFN-beta and IFN-lambda mRNA). Data in Fig. 2 seem to be solely technical duplicates or triplicates - this is not really sufficient. The luciferase and qPCR data with ORF9 should be shown as biological triplicates for a wider range of genes, and the effect on protein secretion should be confirmed by e.g. CXCL10 or CCL5 ELISA or interferon bio-assays.

The data in Fig 2 show technical triplicates in (A), the results of three independent biological experiments in (B), technical triplicates in (C), and technical duplicates in (E). We would like to point out that IFN luciferase data for ORF9 after cGAS/STING overexpression shown in

(A), (B) and (C) represent a total of five biological repeats. Furthermore, *IFNB1* expression after DNA stimulation of ORF9- and GFP-THP1 cells is shown in Fig 2E (one experiment with technical duplicates) and Fig 6E (pooled results from two biological repeats). We believe that this represents sufficient biologically independent data. To address the reviewer's point regarding testing expression of a wider range of ISGs, we included qPCR probes for the ISGs *IFIT1* and *IFI44* in new experiments (Fig 2F-K).

6) Fig. 2C should be re-organised to be more intuitive to interpret: cGAS/STING-stimulated EV and ORF9 samples should be shown side-by-side, followed by RIG-I-CARD-stimulated samples (EV, ORF9). The effect of IRF9 on unstimulated cells should also be shown. If re-organised, it would be clear that ORF9 may also affect RIG-I signalling. This is fine, as many viral proteins are multi-functional, but the statement that ORF9 to "selectively block reporter activation 231 by cGAS/STING but not RIG-I-CARD when compared to empty vector" should be corrected.

We re-organised the data as suggested. The luciferase values in unstimulated cells are indistinguishable between cells transfected with ORF9 expression plasmid and empty vector. We included these data in the updated Fig 2C of the revised manuscript.

7) Given that ORF9 may affect both DNA and RNA sensing, it cannot be assumed that the reduction in basal and DNA-induced interferon-beta and -lambda mRNA levels (Fig. 2E) is solely due to an effect on cGAS. The THP1 cells stably expressing ORF9 should be used to show potential effects on RNA sensing, and on some of the steps in the DNA sensing pathway (e.g. the phosphorylation of STING, TBK1 and IRF3, or STING clustering). This would show more clearly that the main function of ORF9 is upstream of STING activation. The authors could also test the function of ORF9 upstream of downstream of STING activation using luciferase assays to compare the effect on STING over-expression in the presence or absence of cGAS.

We thank the reviewer for raising this point; a similar comment was made by reviewer 1. We performed additional experiments in which STING was stimulated directly with the agonist diABZi. STING activation was not affected by the presence of ORF9 (new Fig 2F). We believe this shows cGAS specificity.

8) In Fig. 3G, the immuno-precipitation of HA-cGAS pulls down both Flag-ORF9 and Flag-GFP. The authors should either make HA pull-down conditions more stringent, or remove this part of the experiment, as the cGAS-ORF9 interaction is already demonstrated in the Flag IP and other experiments in this manuscript.

Fig 3G shows immunoprecipitation of recombinant proteins in a cell-free context. ORF9 and cGAS were purified from *E.coli* and are thus free of other mammalian proteins. The fact that both proteins interact in this cell-free system demonstrates that the binding is independent of additional cellular proteins. This information is not provided by the other IP experiments in our manuscript. Immunoprecipitation of ORF9 and GFP via FLAG clearly shows that only ORF9 interacts with cGAS, not GFP. As pointed out by the reviewer, the reverse IP of cGAS via HA is less clear since both proteins are pulled down (although the interaction between ORF9 and cGAS is stronger). We believe that this is due to a propensity of cGAS to oligomerise and these oligomers might bind GFP non-specifically. Given that we have data from multiple experimental setups demonstrating the cGAS – ORF9 interaction, we believe that repeats of this particular experiment are not a high priority.

9) Given the DNA binding ability of ORF9, key IP experiments should be repeated in the presence of DNase or benzonase, to provide additional evidence for DNA-independent interaction.

We thank the reviewer for this important suggestion. In fact, we tried very hard to use specific nucleases during immunoprecipitation in order to test DNA/RNA dependency of the ORF9-cGAS interaction. Unfortunately, nuclease activities are difficult to control for in high-salt environments such as cell lysis buffers. As a result, we were unable to generate clean and reproducible data that would clearly determine DNA-dependency of the interaction. We hope to address this point as part of future mechanistic studies.

10) In Fig.4B the lanes with construct VIII look like they come from a different experiment with higher cGAS input, so in this case an EV control (or non-interacting fragment) should also be shown for this experiment, to show IP specificity.

The reviewer is correct in that Fig 4B was assembled from two separate experiments. The experiment in which we tested the interaction between cGAS and ORF9-VIII included both GFP and ORF9-WT. We included these data in our revised manuscript.

11) Fig. 5B (liquid droplet formation experiment) needs a control with an unrelated protein or ORF9-N. It seems that this experiment contains twice as much cGAS as ORF9 - could it be possible that a larger ORF9:cGAS ratio is needed for inhibition of droplet formation? Viral proteins may be expressed far in excess of host factors in cells.

We thank the reviewer for this suggestion and repeated our phase separation experiments using different ORF9:cGAS ratios and included ORF9-N as a negative control (see new Fig 5 and EV4). Interestingly, larger droplets were formed by cGAS and DNA when ORF9, but not ORF9-N, was added. This observation held true at three different ORF9:cGAS ratios. Of note, at a higher salt concentration, the intensity of the cGAS signal, but not of the DNA signal, was diminished by ORF9. These data hint at a complex relationship between cGAS, DNA and ORF9 that requires further future investigation.

12) The authors could try to measure inhibition of cGAMP production also in cells (e.g. THP1 cells stably expressing ORF9), using LC-MS or cGAMP ELISA. This would further show that the effect that the authors describe (mainly observed in vitro) indeed also happens in living cells, and that downstream consequences on e.g. the interferon response are indeed a direct result of this particularly molecular mechanism, and not an additional function of ORF9.

We thank the reviewer for this suggestion and agree that measuring cGAMP directly would be informative. In our experience, however, measurement of cGAMP from cell extracts is technically quite challenging using the cGAMP ELISA. Similarly, mass spectrometry measurements of cGAS require an established technical infrastructure that we do not have access to given the current COVID restrictions. The proposed experiments were therefore outside the scope of what we could do in regard to additional experiments for the revised manuscript.

13) While it will probably not be possible to construct deletion viruses to demonstrate the effect of ORF9 during infection, it might be conceivable to use ORF9 RNAi, in combination with the quantification of early responses (within the first hours of infection) prior to viral replication etc.

We agree that testing the effect of ORF9 in infected cells is an important part of describing ORF9 as a cGAS antagonist. As mentioned by the reviewer, it is not possible to construct an

ORF9-deletion virus since the gene is essential for viral replication. ORF9 is further a tegument protein and gets delivered into infected cells by the virion. Generating an ORF9-deletion virus in a complementing cell-line would therefore not be informative either since ORF9 protein would be part of the virion even if it was deleted genetically. To circumvent these issues, we have obtained a virus in which ORF9 is fused to a degron motif. We have used this system to test the effect of ORF9 during infection and found increased IFN responses when ORF9 was acutely degraded upon infection (new Fig 2H-K). A more detailed experimental description is provided in response to reviewer 1, major point 2.

Minor points:

1) Some of the early supplemental figures should go into the main results, e.g. Fig. S3B, S3D.

We thank the reviewer for this suggestion. A similar point was raised by reviewer 1 and we have outlined in our response above the reasoning for showing the data on THP1 infection by co-culture as an Expanded View figure.

2) Some of the results are described without referring to a figure number.

We apologise for this oversight and have added figure numbers in our revised manuscript.

3) Would it be possible to show the results of the ORF screen with the RIG-I-CARD stimulation? It may not be necessary to mention these results (as the effect of ORF9 on RNA sensing is shown) - but if mentioned, the data should be provided in my opinion.

We have removed the mention of these results, which we believe should form part of a separate study.

4) The immunoblot in Fig. 2D should be re-run - at the moment the blot is assembled from individual bands and is lacking an EV control.

The data presented in Fig 2D is derived from THP1 stably transduced with either ORF9 or GFP. As such, we cannot provide data on an empty vector control.

5) The sections on structural similarities between the proteins from different viruses and evolutionary conservation is a little vague and maybe not entirely relevant without additional data.

We thank the reviewer for their comment but politely disagree in terms of relevance to our findings. While experimental testing of related proteins from other viruses is outside the scope of our study, we believe that our model, even if somewhat speculative, is still conceptually interesting. The possibility that a mechanism of cGAS inhibition is evolutionarily old opens up the possibility that many other viruses relevant to human health antagonise cGAS in a similar way. We believe that our concept is of interest to many other scientists working on pathogen-host interaction.

Reviewer #3 (Significance (Required)):

This manuscript describes a novel viral inhibitor of cGAS activation, even though the precise mechanism of action and physiological relevance is not fully elucidated. This manuscript would be of interest to the virology and innate immunity fields.

My expertise: innate immune signalling, DNA sensing, viral immune evasion.

References

Sen N, Sommer M, Che X, White K, Ruyechan WT, Arvin AM (2010) Varicella-Zoster Virus Immediate-Early Protein 62 Blocks Interferon Regulatory Factor 3 (IRF3) Phosphorylation at Key Serine Residues: a Novel Mechanism of IRF3 Inhibition among Herpesviruses. *J Virol* 84: 9240-9253

Vandevenne P, Lebrun M, El Mjiyad N, Ote I, Di Valentin E, Habraken Y, Dortu E, Piette J, Sadzot-Delvaux C (2011) The varicella-zoster virus ORF47 kinase interferes with host innate immune response by inhibiting the activation of IRF3. *PloS one* 6: e16870

Verweij MC, Wellish M, Whitmer T, Malouli D, Lapel M, Jonjic S, Haas JG, DeFilippis VR, Mahalingam R, Fruh K (2015) Varicella Viruses Inhibit Interferon-Stimulated JAK-STAT Signaling through Multiple Mechanisms. *PLoS Pathog* 11: e1004901

Zhu H, Zheng C, Xing J, Wang S, Li S, Lin R, Mossman KL (2011) Varicella-Zoster Virus Immediate-Early Protein ORF61 Abrogates the IRF3-Mediated Innate Immune Response through Degradation of Activated IRF3. *J Virol* 85: 11079-11089

1st Revision - Editorial Decision 28th Mar 2022

Dear Jan,

Thank you for submitting your revised manuscript to The EMBO Journal. Your study has now been seen by referees # 1 and 2 and their comments are provided below. As you can see, both referees appreciate the introduced changes and support publication here. They have a few minor comments that I would like to ask in a last round of revision.

When you submit your revised manuscript will you also take care of the following editorial points:

- The COI section needs to be updated to "Disclosure statement and competing interests.

- Include the funding information in the acknowledgement section.

- Please check figure callouts to Fig EV2B, EV4A-D, Appendix Fig S1A-E, S2A-C.

- "Appendix Tab S#" should be corrected to "Appendix Table S#".

- We encourage the publication of source data, particularly for electrophoretic gels and blots, with the aim of making primary data more accessible and transparent to the reader. It would be great if you could provide me with a PDF file per figure that contains the original, uncropped and unprocessed scans of all or key gels used in the figure? The PDF files should be labeled with the appropriate figure/panel number, and should have molecular weight markers; further annotation could be useful but is not essential. The PDF files will be published online with the article as supplementary "Source Data" files.

- Summary" should be re-labeled as "Abstract".

- Please remove template instructions from the Reagent Table and make this table B&W.

- Our publisher has also done their pre-publication check on your manuscript. When you log into the manuscript submission system you will see the file "Data Edited Manuscript file". Please take a look at the word file and the comments regarding the figure legends and respond to the issues.

That should be all. Let me know if there is anything further to discuss

Best Karin

Karin Dumstrei, PhD
Senior Editor
The EMBO Journal

Instructions for preparing your revised manuscript:

Guide For Authors: https://www.embopress.org/page/journal/14602075/authorguide

We realize that it is difficult to revise to a specific deadline. In the interest of protecting the conceptual advance provided by the work, we recommend a revision within 3 months (26th Jun 2022). Please discuss the revision progress ahead of this time with the editor if you require more time to complete the revisions. Use the link below to submit your revision:

https://emboj.msubmit.net/cgi-bin/main.plex

Referee #1:

The authors have made some important improvements to this manuscript, and added additional experimental evidence to support their discovery that the VZV protein ORF9 acts as DNA sensing inhibitor by interacting with cGAS. The authors have made an effort to address many of the reviewers' comments, and their addition of further control experiments, an improved co-culture protocol and experiments with a STING agonist have, in my opinion, made the study solid and convincing.
While several viral inhibitors of cGAS have been described, and the mechanism of action (via direct cGAS binding) is not defined in great mechanistic detail, I believe that the findings are interesting for readers of the innate immunity and virology fields, and the conclusions are well supported by the experimental evidence.

I am largely satisfied with the manuscript as it stands, and only have some minor comments:

- In the results section, the authors propose that it is possible that ORF9 replaces cGAS from the DNA (line 426-428) based on observations from phase separation experiments and another published manuscript (Xu et al, 2021). However, the remainder of the data presented here does not support that model, with no competition in oligonucleotide pull-down experiments, and no difference in the inhibition of cGAMP production and interferon induction between ORF9 and a mutant with reduced DNA binding capacity. While this is later clarified in the discussion, care should be taken not to over-interpret the observations from the phase separation experiments at that point.
- It is still not always clear what the shown data represents (even though this aspect is now much improved from the last version) - are individual experiments each performed as triplicates, and the data point shown in the aggregate graph represents the averages (and hence the use of SEM), or do experiments only have one data point each? How data points are compared in Fig. 1J is particularly unclear.
- Some of the graphs are visually a little unclear - this could be improved by increasing the separation between experimental groups, e.g. 24h and 48h, or + and - VZV in Fig. 1C for instance, and adding labels to the X axis where missing (e.g. 2F, G).
- The text size of the P values in the figure is too small.
- Fig 1G only has two data points per sample and is not convincing, given the low fold induction and comparatively high variability between the two data points. Either add additional repeats, or move to the supplemental data section.

Referee #2:

The new data shown in Fig 2F-K adds direct evidence for a key role of ORF9 in the repression of cGAS mediated antiviral response. The authors have appropriately addressed my other comments and the paper is now suitable for publication.

Manuscript EMBOJ-2021-109217R1

Point-by-point response

Referee #1:

The authors have made some important improvements to this manuscript, and added additional experimental evidence to support their discovery that the VZV protein ORF9 acts as DNA sensing inhibitor by interacting with cGAS. The authors have made an effort to address many of the reviewers' comments, and their addition of further control experiments, an improved co-culture protocol and experiments with a STING agonist have, in my opinion, made the study solid and convincing.
While several viral inhibitors of cGAS have been described, and the mechanism of action (via direct cGAS binding) is not defined in great mechanistic detail, I believe that the findings are interesting for readers of the innate immunity and virology fields, and the conclusions are well supported by the experimental evidence.

We welcome that the reviewer appreciates the changes we have made and are grateful for the positive comments. Text changed or added during the second round of revisions is shown in green, with earlier revisions in red.

I am largely satisfied with the manuscript as it stands, and only have some minor comments:

- In the results section, the authors propose that it is possible that ORF9 replaces cGAS from the DNA (line 426-428) based on observations from phase separation experiments and another published manuscript (Xu et al, 2021). However, the remainder of the data presented here does not support that model, with no competition in oligonucleotide pull-down experiments, and no difference in the inhibition of cGAMP production and interferon induction between ORF9 and a mutant with reduced DNA binding capacity. While this is later clarified in the discussion, care should be taken not to over-interpret the observations from the phase separation experiments at that point.

We believe the reviewer refers to line 416-418. We have changed the wording here: Instead of "It is therefore possible that ORF9 displaces cGAS from DNA" the sentence now reads "One of multiple possible interpretations is that ORF9 displaces cGAS from DNA".

- It is still not always clear what the shown data represents (even though this aspect is now much improved from the last version) - are individual experiments each performed as triplicates, and the data point shown in the aggregate graph represents the averages (and hence the use of SEM), or do experiments only have one data point each? How data points are compared in Fig. 1J is particularly unclear.

We have added further clarifications to the figure legends. If the panel is representative of multiple experiments, data points represent technical replicates from one independent biological experiment. If a panel shows pooled data, then each data point represents an independent biological experiment.

- Some of the graphs are visually a little unclear - this could be improved by increasing the separation between experimental groups, e.g. 24h and 48h, or + and - VZV in Fig. 1C for instance, and adding labels to the X axis where missing (e.g. 2F, G).

We appreciate the suggestion from the reviewer and have changed Figures 1C,E,F, and G. We have further added the x-axes legends to Figures 2F and G (the latter is now Fig EV3B).

- The text size of the P values in the figure is too small.

We have increased the font size as requested.

- Fig 1G only has two data points per sample and is not convincing, given the low fold induction and comparatively high variability between the two data points. Either add additional repeats, or move to the supplemental data section.

We believe the reviewer refers to Fig 2G here. As suggested by the reviewer, we have moved panel 2G to the supplement (now: panel EV3B).

Referee #2:

The new data shown in Fig 2F-K adds direct evidence for a key role of ORF9 in the repression of cGAS mediated antiviral response. The authors have appropriately addressed my other comments and the paper is now suitable for publication.

We welcome that the reviewer appreciates the changes made and are grateful that she/he recommends publication.

Accepted **9th May 2022**

Hi Jan,

Thank you for submitting your revised manuscript to The EMBO Journal. I have now had a chance to look at everything and all looks good.

I am therefore very pleased to accept the manuscript for publication here.

Congratulations about a nice study!

With best wishes

Karin

Karin Dumstrei, PhD
Senior Editor
The EMBO Journal

Please note that it is EMBO Journal policy for the transcript of the editorial process (containing referee reports and your response letter) to be published as an online supplement to each paper. If you do NOT want this, you will need to inform the Editorial Office via email immediately. More information is available here: https://www.embopress.org/page/journal/14602075/authorguide#transparentprocess

Your manuscript will be processed for publication in the journal by EMBO Press. Manuscripts in the PDF and electronic editions of The EMBO Journal will be copy edited, and you will be provided with page proofs prior to publication. Please note that supplementary information is not included in the proofs.

Please note that you will be contacted by Wiley Author Services to complete licensing and payment information. The required 'Page Charges Authorization Form' is available here: https://www.embopress.org/pb-assets/embo-site/tej_apc.pdf

Should you be planning a Press Release on your article, please get in contact with embojournal@wiley.com as early as possible, in order to coordinate publication and release dates.

If you have any questions, please do not hesitate to call or email the Editorial Office. Thank you for your contribution to The EMBO Journal.

** Click here to be directed to your login page: https://emboj.msubmit.net

EMBO Press Author Checklist

Corresponding Author Name: Jan Rehwinkel
Journal Submitted to: The EMBO Journal
Manuscript Number: EMBOJ-2021-109217R1 | [RC-2021-00768] [REV]

USEFUL LINKS FOR COMPLETING THIS FORM
The EMBO Journal - Author Guidelines
EMBO Reports - Author Guidelines
Molecular Systems Biology - Author Guidelines
EMBO Molecular Medicine - Author Guidelines

Reporting Checklist for Life Science Articles (updated January 2022)
This checklist is adapted from Materials Design Analysis Reporting (MDAR) Checklist for Authors. MDAR establishes a minimum set of requirements in transparent reporting in the life sciences (see Statement of Task: 10.31222/osf.io/9sm4x). Please follow the journal's guidelines in preparing your manuscript.
Please note that a copy of this checklist will be published alongside your article.

Abridged guidelines for figures
1. Data
The data shown in figures should satisfy the following conditions:
- the data were obtained and processed according to the field's best practice and are presented to reflect the results of the experiments in an accurate and unbiased manner.
- ideally, figure panels should include only measurements that are directly comparable to each other and obtained with the same assay.
- plots include clearly labeled error bars for independent experiments and sample sizes. Unless justified, error bars should not be shown for technical replicates.
- if n<5, the individual data points from each experiment should be plotted. Any statistical test employed should be justified.
- Source Data should be included to report the data underlying figures according to the guidelines set out in the authorship guidelines on Data Presentation.

2. Captions
Each figure caption should contain the following information, for each panel where they are relevant:
- a specification of the experimental system investigated (eg cell line, species name).
- the assay(s) and method(s) used to carry out the reported observations and measurements.
- an explicit mention of the biological and chemical entity(ies) that are being measured.
- an explicit mention of the biological and chemical entity(ies) that are altered/varied/perturbed in a controlled manner.
- the exact sample size (n) for each experimental group/condition, given as a number, not a range;
- a description of the sample collection allowing the reader to understand whether the samples represent technical or biological replicates (including how many animals, litters, cultures, etc.).
- a statement of how many times the experiment shown was independently replicated in the laboratory.
- definitions of statistical methods and measures:
  - common tests, such as t-test (please specify whether paired vs. unpaired), simple χ2 tests, Wilcoxon and Mann-Whitney tests, can be unambiguously identified by name only, but more complex techniques should be described in the methods section;
  - are tests one-sided or two-sided?
  - are there adjustments for multiple comparisons?
  - exact statistical test results, e.g., P values = x but not P values < x;
  - definition of 'center values' as median or average;
  - definition of error bars as s.d. or s.e.m.

Please complete ALL of the questions below.
Select "Not Applicable" only when the requested information is not relevant for your study.

Materials

**Newly Created Materials**	**Information included in the manuscript?**	**In which section is the information available?** (Reagents and Tools Table, Materials and Methods, Figures, Data Availability Section)
New materials and reagents need to be available; do any restrictions apply?	Yes	Reagents and Tools Table, Materials and Methods

**Antibodies**	**Information included in the manuscript?**	**In which section is the information available?** (Reagents and Tools Table, Materials and Methods, Figures, Data Availability Section)
For **antibodies** provide the following information:<br>- Commercial antibodies: RRID (if possible) or supplier name, catalogue number and or/clone number<br>- Non-commercial: RRID or citation	Yes	Reagents and Tools Table

**DNA and RNA sequences**	**Information included in the manuscript?**	**In which section is the information available?** (Reagents and Tools Table, Materials and Methods, Figures, Data Availability Section)
**Short novel DNA or RNA including primers, probes**: provide the sequences.	Yes	Reagents and Tools Table, Appendix Tables 1-3

**Cell materials**	**Information included in the manuscript?**	**In which section is the information available?** (Reagents and Tools Table, Materials and Methods, Figures, Data Availability Section)
**Cell lines:** Provide species information, strain. Provide accession number in repository **OR** supplier name, catalog number, clone number, and/**OR** RRID.	Yes	Reagents and Tools Table, Materials and Methods
**Primary cultures:** Provide species, strain, sex of origin, genetic modification status.	Not Applicable	
Report if the cell lines were recently **authenticated** (e.g., by STR profiling) and tested for mycoplasma contamination.	Yes	Materials and Methods

**Experimental animals**	**Information included in the manuscript?**	**In which section is the information available?** (Reagents and Tools Table, Materials and Methods, Figures, Data Availability Section)
**Laboratory animals or Model organisms:** Provide species, strain, sex, age, genetic modification status. Provide accession number in repository **OR** supplier name, catalog number, clone number, **OR** RRID.	Not Applicable	
**Animal observed in or captured from the field:** Provide species, sex, and age where possible.	Not Applicable	
Please detail **housing and husbandry conditions**.	Not Applicable	

**Plants and microbes**	**Information included in the manuscript?**	**In which section is the information available?** (Reagents and Tools Table, Materials and Methods, Figures, Data Availability Section)
**Plants:** provide species and strain, ecotype and cultivar where relevant, unique accession number if available, and source (including location for collected wild specimens).	Not Applicable	
**Microbes:** provide species and strain, unique accession number if available, and source.	Yes	Materials and Methods

**Human research participants**	**Information included in the manuscript?**	**In which section is the information available?** (Reagents and Tools Table, Materials and Methods, Figures, Data Availability Section)
If collected and within the bounds of privacy constraints report on age, sex and gender or ethnicity for all study participants.	Not Applicable	

**Core facilities**	**Information included in the manuscript?**	**In which section is the information available?** (Reagents and Tools Table, Materials and Methods, Figures, Data Availability Section)
If your work benefited from core facilities, was their service mentioned in the acknowledgments section?	Yes	Acknowledgements and Funding

Design

Study protocol	Information included in the manuscript?	In which section is the information available? (Reagents and Tools Table, Materials and Methods, Figures, Data Availability Section)
If study protocol has been **pre-registered, provide DOI in the manuscript**. For clinical trials, provide the trial registration number **OR** cite DOI.	Not Applicable	
Report the **clinical trial registration number** (at ClinicalTrials.gov or equivalent), where applicable.	Not Applicable	

Laboratory protocol	Information included in the manuscript?	In which section is the information available? (Reagents and Tools Table, Materials and Methods, Figures, Data Availability Section)
Provide DOI OR other citation details if **external detailed step-by-step protocols** are available.	Not Applicable	

Experimental study design and statistics	Information included in the manuscript?	In which section is the information available? (Reagents and Tools Table, Materials and Methods, Figures, Data Availability Section)
Include a statement about **sample size** estimate even if no statistical methods were used.	Yes	Materials and Methods
Were any steps taken to minimize the effects of subjective bias when allocating animals/samples to treatment (e.g. **randomization procedure**)? If yes, have they been described?	Yes	Materials and Methods
Include a statement about **blinding** even if no blinding was done.	Yes	Materials and Methods
Describe **inclusion/exclusion criteria** if samples or animals were excluded from the analysis. Were the criteria pre-established?<br><br>If sample or data points were omitted from analysis, report if this was due to attrition or intentional exclusion and provide justification.	Not Applicable	
For every figure, are **statistical tests** justified as appropriate? Do the data meet the assumptions of the tests (e.g., normal distribution)? Describe any methods used to assess it. Is there an estimate of variation within each group of data? Is the variance similar between the groups that are being statistically compared?	Yes	Materials and Methods

Sample definition and in-laboratory replication	Information included in the manuscript?	In which section is the information available? (Reagents and Tools Table, Materials and Methods, Figures, Data Availability Section)
In the figure legends: state number of times the experiment was **replicated** in laboratory.	Yes	Figure legends
In the figure legends: define whether data describe **technical or biological replicates**.	Yes	Figure legends

Ethics

Ethics	Information included in the manuscript?	In which section is the information available? (Reagents and Tools Table, Materials and Methods, Figures, Data Availability Section)
Studies involving **human participants**: State details of **authority granting ethics approval** (IRB or equivalent committee(s), provide reference number for approval.	Not Applicable	
Studies involving **human participants**: Include a statement confirming that **informed consent** was obtained from all subjects and that the experiments conformed to the principles set out in the WMA Declaration of Helsinki and the Department of Health and Human Services Belmont Report.	Not Applicable	
Studies involving **human participants:** For publication of **patient photos**, include a statement confirming that consent to publish was obtained.	Not Applicable	
Studies involving experimental **animals**: State details of **authority granting ethics approval** (IRB or equivalent committee(s), provide reference number for approval. Include a statement of compliance with ethical regulations.	Not Applicable	
Studies involving **specimen and field samples:** State if relevant **permits** obtained, provide details of authority approving study; if none were required, explain why.	Not Applicable	

Dual Use Research of Concern (DURC)	Information included in the manuscript?	In which section is the information available? (Reagents and Tools Table, Materials and Methods, Figures, Data Availability Section)
Could your study fall under dual use research restrictions? Please check biosecurity documents and list of **select agents and toxins** (CDC): https://www.selectagents.gov/sat/list.htm	Not Applicable	
If you used a select agent, is the security level of the lab appropriate and reported in the manuscript?	Not Applicable	
If a study is subject to dual use research of concern regulations, is the name of the **authority granting approval and reference number** for the regulatory approval provided in the manuscript?	Not Applicable	

Reporting

The MDAR framework recommends adoption of discipline-specific guidelines, established and endorsed through community initiatives. Journals have their own policy about requiring specific guidelines and recommendations to complement MDAR.

Adherence to community standards	Information included in the manuscript?	In which section is the information available? (Reagents and Tools Table, Materials and Methods, Figures, Data Availability Section)
State if relevant guidelines or checklists (e.g., **ICMJE, MIBBI, ARRIVE, PRISMA**) have been followed or provided.	Not Applicable	
For **tumor marker prognostic studies,** we recommend that you follow the **REMARK** reporting guidelines (see link list at top right). See author guidelines, under 'Reporting Guidelines'. Please confirm you have followed these guidelines.	Not Applicable	
For **phase II and III randomized controlled trials**, please refer to the **CONSORT** flow diagram (see link list at top right) and submit the CONSORT checklist (see link list at top right) with your submission. See author guidelines, under 'Reporting Guidelines'. Please confirm you have submitted this list.	Not Applicable	

Data Availability

Data availability	Information included in the manuscript?	In which section is the information available? (Reagents and Tools Table, Materials and Methods, Figures, Data Availability Section)
Have **primary datasets** been deposited according to the journal's guidelines (see 'Data Deposition' section) and the respective accession numbers provided in the Data Availability Section?	Not Applicable	
Were **human clinical and genomic datasets** deposited in a public access-controlled repository in accordance to ethical obligations to the patients and to the applicable consent agreement?	Not Applicable	
Are **computational models** that are central and integral to a study available without restrictions in a machine-readable form? Were the relevant accession numbers or links provided?	Not Applicable	
If publicly available data were reused, provide the respective **data citations in the reference list**.	Not Applicable	
